# Amesilide, a New Bicyclic Polyketide from the Marine Fungus *Amesia nigricolor* MUT6601

**DOI:** 10.3390/molecules30153169

**Published:** 2025-07-29

**Authors:** Giang Nam Pham, Matteo Florio Furno, Juan A. Garcia-Sanchez, Patrick Munro, Fatouma Mohamed Abdoul-Latif, Laurent Boyer, Giovanna Cristina Varese, Mohamed Mehiri

**Affiliations:** 1 Marine Natural Products Team, Institut de Chimie de Nice, Université Côte d’Azur, CNRS UMR 7272, 06108 Nice, France; nam.phamgiang@phenikaa-uni.edu.vn; 2 Department of Life Sciences and Systems Biology, University of Torino, Mycotheca Universitatis Taurinensis (MUT), Viale Mattioli 25, 10125 Torino, Italy; 3 INSERM U1065, Centre Méditerranéen de Médecine Moléculaire (C3M), Bâtiment Universitaire ARCHIMED, 151 Route de Saint Antoine de Ginestière BP, 23194 Nice, France; 4 Medicinal Research Institute, Center for Studies and Research of Djibouti, IRM-CERD, Route de l’Aéroport, Haramous, Djibouti City P.O. Box 486, Djibouti; fatouma_abdoulatif@yahoo.fr

**Keywords:** *Amesia nigricolor*, cytochalasan, polyketide, *bis*-naphtho-*γ*-pyrone, antimicrobial, cytotoxicity

## Abstract

A new bicyclic polyketide, amesilide (**1**), along with the previously reported metabolites, chamisides A (**2**), B (**3**), and E (**4**), chaetoconvosins B (**5**) and C (**6**), and chaetochromins A (**7**) and B (**8**), were isolated from the marine fungus *Amesia nigricolor* MUT6601. The structures of the compounds were determined by extensive spectrometric (HRMS) and spectroscopic (1D and 2D NMR) analyses, as well as specific rotation. Absolute configurations of the stereogenic centers of amesilide (**1**) were determined by a comparison of its experimental circular dichroism (CD) spectrum with its time-dependent density functional theory (TD-DFT) electronic circular dichroism (ECD) spectra. Among them, chaetochromins A (**7**) and B (**8**) showed strong antibacterial activity against *Staphylococcus aureus* S25 (MBC values of 12.50 µM and MIC values of 6.25 µM) and a moderate cytotoxicity against monocytes (THP-1) and peripheral blood cells (PBMC) (IC_50_ values of 33.65–40.01 µM).

## 1. Introduction

Marine fungi, which usually produce metabolites different from their terrestrial counterparts, represent important sources of bioactive metabolites for drug discovery [1]. Indeed, reports of new natural products from marine-derived fungi have significantly increased over the last few decades [2]. *Chaetomium* and *Chaetomium*-like species (Chaetomiaceae) have been proven to be rich sources of specialized metabolites [3,4,5]. The taxonomy of this genus has recently been revised based on phylogenetic analyses and morpho-physiological characteristics [6]. Currently, the family Chaetomiaceae comprises 10 genera which include five newly established genera, including the genus *Amesia* [6].

*Amesia nigricolor* (L.M. Ames) X. Wei Wang and Samson (synonym *Chaetomium nigricolor*) has latterly attracted the attention of several researchers. Several metabolites recently isolated from *A. nigricolor* have demonstrated anti-inflammatory or cholesterol-lowering activities [4,7,8,9]. Kim et al. identified twelve metabolites from *A. nigricolor* isolated from soil [7], finding a *bis*-naphtho-γ-pyrone derivative ((aS)-asperpyrone A) as an important anti-inflammatory component. In another study, Dhayanithy et al. reported that the extract of the endophytic fungus *A. nigricolor*, which was isolated from *Catharanthus roseus*, exhibited potent cytotoxicity and antioxidant properties [10]. Chamiside A, a cytochalasin with a tricyclic core skeleton, was identified by Wang et al. and found to display a moderate antibacterial activity against *Staphylococcus aureus* [8]. Recently, Gu et al. continued the chemical investigation of the same strain of *A. nigricolor* F5 of the latter study, discovering five novel chytochalasans, Chamisides B-F [10]. Interestingly, Chamiside F showed a promising bioactivity, with inhibitory activity against the cholesterol transporter Niemann-Pick C1-like 1 (NPC1L1) protein, reducing the cholesterol absorption.

In this context, we investigated the metabolites produced by the marine fungus *A. nigricolor* MUT6601. Herein, we report the isolation, structural elucidation, and antimicrobial activities of a new bicyclic polyketide, amesilide (**1**), along with the previously reported metabolites, chamisides A (**2**), B (**3**), and E (**4**), chaetoconvosins B (**5**) and C (**6**), and chaetochromins A (**7**) and B (**8**) from the marine fungus *A. nigricolor* MUT6601. All of the isolated compounds were evaluated for their antimicrobial properties and cytotoxicity.

## 2. Results and Discussion

**Figure 1:**
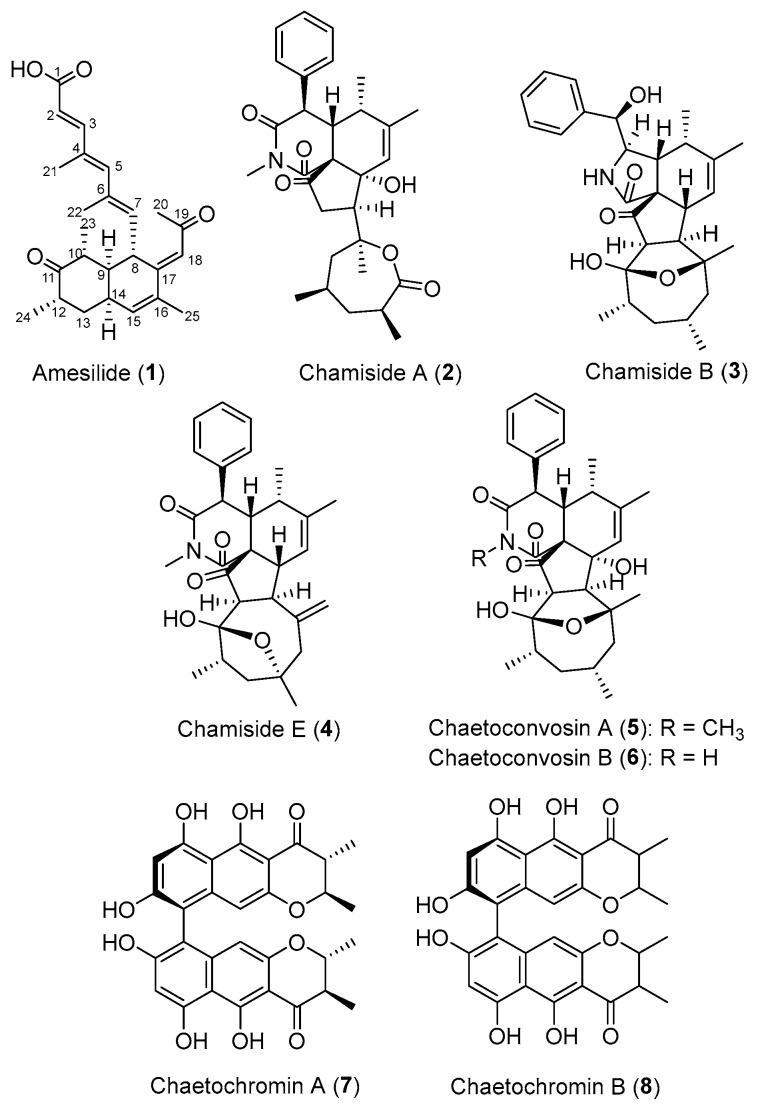
Structures of compounds **1**–**8** isolated from the fungus *A. nigricolor* MUT6601.

The EtOAc extract of *A. nigricolor* MUT6601 was fractionated and purified by a combination of C-18 solid-phase extraction, silica gel column chromatography, and semi-preparative HPLC to yield compounds **1**–**8** (Figure 1).

**Figure 2:**
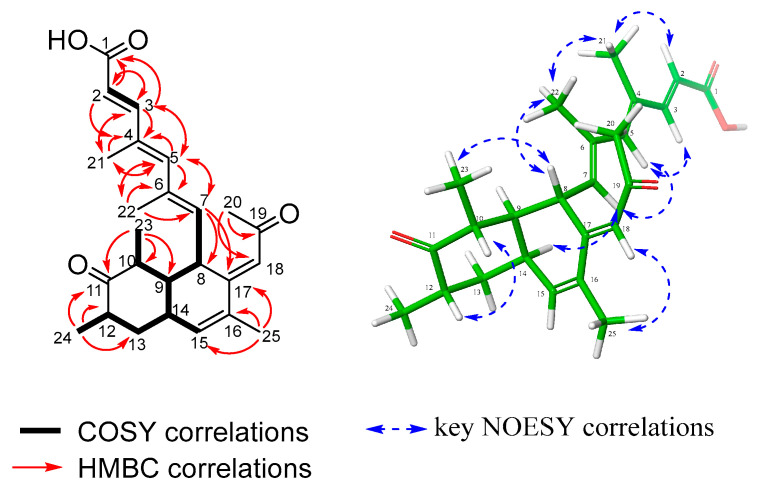
Key COSY, HMBC, and NOESY correlations of amesilide (**1**).

Amesilide (**1**) was isolated as a yellow powder. Its molecular formula, C_25_H_32_O_4_, was deduced from the HRESI(+)MS analysis which showed a pseudo-molecular ion peak at *m/z* 397.2369 [M+H]^+^ (calcd for C_25_H_33_O_4_^+^ 397.2373, Δ = 1.01 ppm, 10 degrees of unsaturation). The ^1^H, ^13^C, and ^1^H-^13^C HSQC NMR spectra indicated the presence of six methyl groups (*δ*c 32.7, 20.1, 16.8, 14.6, 13.9, and 11.5), one sp^3^ methylene (*δ*c 41.3), five sp^3^ methines (*δ*c 50.9, 44.5, 41.2, 37.5, and 33.5), six sp^2^ methines (*δ*c 152.9, 144.7, 139.3, 134.0, 122.9, and 115.6), and seven quaternary sp^2^ carbons (*δ*c 213.8, 198.8, 172.0, 151.1, 135.4, 133.4, and 131.9), including one ketone (*δ*c 213.8), one *α*,*β*-unsaturated ketone (*δ*c 198.8), and one *α*,*β*-unsaturated carboxylic acid (*δ*c 172.0) (Table 1). The assembly of the ^1^H-^13^C HMBC correlations and ^1^H-^1^H COSY spin systems revealed that **1** has a bicyclic decalin system. The structure of compound **1** was established based on key ^1^H-^13^C HMBC correlations, including H-23 to C-9, C-10, and C-11; H-24 to C-11, C-12, and C-13; H-25 to C-15, C-16, and C-17; H-7 to C-8, C-9, and C-17; and H-18 to C-8, C-16, and C-17. The COSY spectrum showed continuous spin systems (H-7 to H-23 and H-24 to H-15), further supporting this framework. The locations of a ketone group at C-11, three methyl groups attached to C-10, C-12, and C-16, and two substituted chains attached to C-8 and C-17 were also determined through the above correlations. One substituent was determined as 4,6-dimethylhepta-2,4,6-trienoic acid by the COSY correlation between H-2 and H-3, and the HMBC correlations from H_3_-21 to C-3, C-4, and C-5 and from H_3_-22 to C-5, C-6, and C-7. The other substituent was determined by the HMBC correlations from the methyl group H-20 to C-18 and C-19 and from H-18 to C-19 and C-20 (Table 1). The planar structure of **1** was then established as shown in Figure 2.

The relative configuration of **1** was determined by the analyses of vicinal coupling constants and the NOESY correlations. The geometry of the double bond at C-2/C-3 was determined as *E*, by the large vicinal coupling constant between H-2 and H-3 (*J*_2,3_ = 15.5 Hz), and the ^1^H-^1^H NOESY correlation of H-2/H-21. Then, the NOESY correlations of H-21/H-22, H-3/H-5, and H-5/H-7 indicated both double bonds C-4/C-5 and C-6/C-7 were *E*. The geometry of the olefin C-17/C18 was established as *E* by the key NOESY correlation of H-18/H-25. The NOESY correlations of H-8/H-23 and H-7/H-14 indicated a *cis*-fused ring junction. A small coupling constant of less than 1 Hz between H-9 and H-14 indicated that the angle between them was near 90°, providing stronger confirmation for the *cis*-fused decalin system. The NOESY correlation of H-10/H-12 suggested they were both axial and co-facial.

The most unusually distinctive feature of the NMR spectral dataset of **1** is the downfield shift of H-8 at *δ*_H_ 5.15, which is directly connected to a carbon at *δ*_C_ 37.5. However, this phenomenon has also been reported in polyketides with structures closely related to **1**, isolated from a yew-associated *Penicillium* species by Stierle et al. [11]. This may be due to the anisotropy effect generated by the C-19 ketone group, which deshields H-8. The authors attempted to reduce the C-19 carbonyl group using NaBH_4_, yielding two products in which the chemical shifts of H-8 decreased to approximately *δ*_H_ 3.82–3.86, confirming the effect of the carbonyl group. However, preussilide C, another polyketide from the endophytic fungus *Preussia similis*, has an almost identical structure to **1**, including a carbonyl group at C-19 but differing in the arrangement of its double bonds [12]. Despite this similarity, it exhibits an H-8 shift of only *δ*_H_ 3.26. This suggests that the C-15 to C-19 double bond system also plays a role in causing H-8 to shift toward the downfield region. Hence, the distinctive downfield signal of H-8 is a very coincidental effect of many factors, in which H-8 is both the allylic proton of the two conjugated double bond systems from C-1 to C-7 and C-15 to C-19, and is also influenced by the anisotropic effect from the carbonyl group at C-19. To provide further solid evidence for this phenomenon, the ^1^H and ^13^C chemical shifts of **1** were also calculated for comparison.

**Figure 3:**
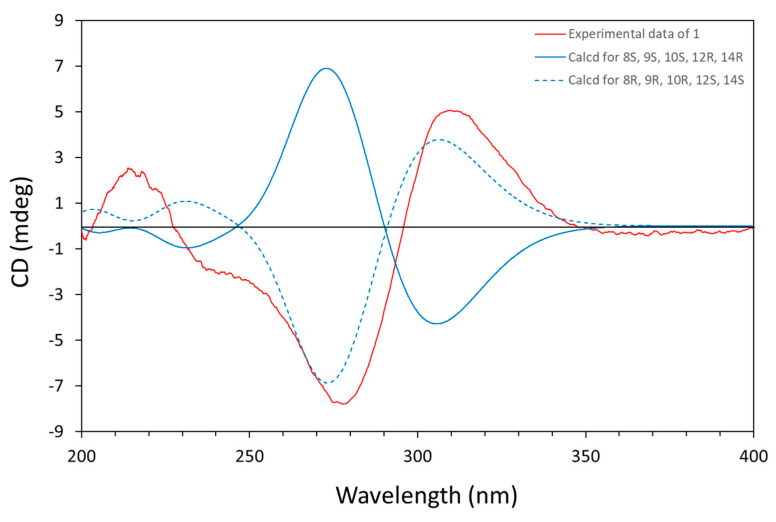
Experimental CD spectrum for amesilide (**1**) in CH_3_CN and calculated ECD spectra for **1a** (8*R*, 9*R*, 10*R*, 12*S*, and 14*S*) and **1b** (8*S*, 9*S*, 10*S*, 12*R*, and 14*R*).

The experimental CD spectrum of **1** exhibited a negative Cotton effect (CE) at *λ*_max_ = 278 nm and one positive CE at *λ*_max_ = 310 nm (Figure 3). The Boltzmann-averaged TD-DFT calculated ECD spectrum for the most stable conformers of the enantiomer **1a** (8*R*, 9*R*, 10*R*, 12*S*, and 14*S*), performed at the CAM-B3LYP/6–31G(d,p) level of theory (IEFPCM, acetonitrile), also showed one negative CE at *λ*_max_ = 275 nm and one positive CE at *λ*_max_ = 305 nm, which reproduced the signs and differences in amplitude of the experimental CEs. Thus, the absolute configuration of **1** was determined as 8*R*, 9*R*, 10*R*, 12*S*, and 14*S* (Figure 3, Tables S1–S25).

Seven already reported compounds were identified as chamisides A (**2**), B (**3**), and E (**4**) [8,9], chaetoconvosins B (**5**) [13] and C (**6**) [9], and chaetochromins A (**7**) and B (**8**) [14] by comparing their HRESIMS, NMR data, and specific rotations with those reported in the literature. The relative stereochemistry of chaetochromin B (**8**) remained unclarified.

All isolated compounds were evaluated for their antimicrobial activities against a panel of microorganisms, including *Staphylococcus aureus* S25, *Escherichia coli* UTI89, *Candida krusei* CK01, *Candida albicans* CA01, and *Leishmania infantum* (LI01). Only chaetochromins A (**7**) and B (**8**) exhibited strong activity against *S. aureus* S25, with MIC values of 6.25 µM (inhibiting bacterial growth) and MBC values of 12.50 µM (killing 99.9% of bacteria). The strong antibacterial activity of chaetochromin A against *S. aureus* is also consistent with previous reports [15]. More interestingly, despite showing strong antibacterial activity, these compounds showed no cytotoxicity against human erythrocytes (RBC), and moderate activities against human monocytes (THP-1) and peripheral blood mononuclear cells (PBMC) (IC_50_ values of 33.65–40.01 µM, Table S1). These results encourage further investigation into the potential clinical applications of compounds **7** and **8**.

## 3. Materials and Methods

### 3.1. General Experimental Procedure

Optical rotations were determined using an Anton Paar MCP 150 polarimeter (Anton Paar, Graz, Austria). Fourier transform infrared (FT-IR) spectra were recorded on a Nicolet iS50 FT-IR spectrometer (Thermo Scientific, Waltham, MA, USA). Semi-preparative chromatography and high-performance liquid chromatography (HPLC) analyses were performed using a Waters Alliance e2695 HPLC system (Waters Corporation, Milford, MA, USA), equipped with a bifunctional Macherey-Nagel NUCLEODUR Sphynx RP column (250 × 4.6 mm or 250 × 10.0 mm, 5 µm) connected to both a Waters 2424 evaporative light scattering (ELS) detector and a Waters 2998 photodiode array (PDA) detector. HPLC-grade solvents were purchased from Sigma-Aldrich (Merck KGaA, Saint-Louis, MO, USA). NMR spectra were recorded on a 400 MHz Bruker Avance NMR spectrometer (Bruker Corporation, Billerica, MA, USA). Circular dichroism (CD) spectra were measured using a JASCO J-810 spectropolarimeter (JASCO International Co., Ltd., Tokyo, Japan). High-resolution mass spectra (HRMS) were acquired using a Thermo Q-Exactive Focus Orbitrap UPLC-HRMS system (Thermo Fisher Scientific, Waltham, MA, USA), and the data were processed with Thermo Xcalibur software version 2.2.44.

### 3.2. Microorganism Isolation and Identification

*Amesia nigricolor* MUT6601 was isolated from the sediments of the Harbor of Livorno, in the Tyrrhenian Sea in Tuscany (IT; 43°33′43.92″ N 10°17′42″ E). The harbor of Livorno is an area affected by several petrochemical and industrial activities, primarily due to contamination from metals and hydrocarbons [16].

As previously described [16], the dilution plate method was used to isolate fungi: the sediment suspensions were plated on Corn Meal Agar (CMA: 2 gL^−1^ corn meal infusion, 15 g L^−1^ agar) with antibiotics (Gentamicin 80 mgL^−1^ and Tazobactam 100 mgL^−1^) and 2% *w*/*v* of sea salts. All of the chemicals were purchased from Sigma-Aldrich. The identification at species level was carried out through morphological and molecular analyses. The internal transcribed spacer region (ITS) gene sequence obtained for *A. nigricolor* MUT6601 is available at GenBank NCBI under the accession number OP161804.

The strain is deposited at Mycotheca Universitatis Taurinensis (MUT, www.mut.unito.it) of the Department of Life Sciences and Systems Biology, University of Torino, Torino (Italy).

### 3.3. Fungal Solid State Fermentation

The fermentation was carried out following an adaptation of the method previously described by Yue et al. [17]. Briefly, *A. nigricolor* MUT6601 was fermented in solid rice media (RM), in 100 mL bottles containing 20 g of rice and 20 mL of mineral medium (MM: 10 mL/L mineral solution—MS and 1 mL/L trace metal solution—TMS. MS (100 mL): KCl 5 g, MgSO_4_•7H_2_O 5 g, and FeSO_4_•7H_2_O 0.1 g. TMS (100 mL): ZnSO_4_•7H_2_O 1 g and CuSO_4_ 0.5 g.

Prior to the fermentation *A. nigricolor,* MUT6601 was cultured on CMA plates for 7–14 days at 24 °C. A mycelium homogenate was prepared as inoculum: the fungal biomass was collected from CMA plates with a sterilized scalpel and blended using distilled sterilized water (1 cm^2^/1 mL) using an Ultra-turrax^®^ homogenizer (IKA) (IKA, Staufen, Germany) [18].

The solid-state fermentation was carried out in 80 bottles of RM in which 3 mL of mycelium homogenate was inoculated. The fermentation lasted 2 weeks.

### 3.4. Extraction and Purification

The colonized rice was extracted with ethyl acetate, and the resulting solution was concentrated in vacuo to yield 8.96 g of crude organic extract. The total extract was then fractionated by C-18 solid-phase extraction, eluting with water/methanol (100:0, 80:20, 60:40, 40:60, 20:80, 10:90, and 0:100, *v*/*v*), and then methanol/dichloromethane (50:50, *v*/*v*), to give 8 fractions (AN1 → AN8). Fraction AN4 (593.4 mg) was fractionated by silica gel column chromatography, eluting with cyclohexane/ethyl acetate (3:1 → 0:1, *v*/*v*) to yield seven sub-fractions (AN4.1 → AN4.7). Sub-fraction AN4.2 (181.2 mg) was purified by analytical HPLC using acetonitrile/water (55:45, 1 mL/min) containing 0.1% formic acid, to yield **3** (4.0 mg, t_R_ = 8.9 min), **1** (6.4 mg, t_R_ = 13.1 min), **5** (39.4 mg, t_R_ = 14.7 min), and **2** (25.5 mg, t_R_ = 16.2 min). Sub-fraction AN4.3 (43.9 mg) was purified by semi-preparative HPLC using the same mobile phase with a flow rate of 3 mL/min, to yield **6** (2.6 mg, t_R_ = 12.5 min). Fraction AN5 (707.9 mg) was separated by silica gel column chromatography and eluting with cyclohexane/ethyl acetate (9:1 → 0:1, *v*/*v*) to yield **4** (38.2 mg) and eleven other sub-fractions (AN5.1, AN5.3 → AN5.12). Sub-fraction AN5.9 (27.5 mg) was purified by semi-preparative HPLC using acetonitrile/water (65:35, 3 mL/min) containing 0.1% formic acid, to yield **7** (2.0 mg, t_R_ = 14.5 min) and **8** (3.6 mg, t_R_ = 12.0 min).

Amesilide (**1**): yellow powder. [α]_D_^20^ -12.0 (*c* 0.6, CHCl_3_). Molecular formula: C_25_H_32_O_4_. HRESIMS *m*/*z* 397.2369 [M+H]^+^ (calcd for C_25_H_33_O_4_^+^ 397.2373). IR *υ*_max_: 2928.3, 1706.5, 1618.2, 1582.3, 1452.9, 1376.7, 1183.4, and 1024.1. CD (acetonitrile) λ (Δε): 214 (+6.11), 277 (−18.76), and 310 (+12.21). ^1^H and ^13^C NMR data see Table 1.

### 3.5. Computational Details

The conformational searches of each possible isomer were performed by employing MAESTRO software (Schrodinger LLC, New York, NY, USA), using an energy window of 5 kcal/mol, applying 10,000 steps of the Monte Carlo multiple minimum method with PRCG energy minimization using the Merck Molecular Force Field (MMFF) in gas phase, yielding 42 conformers of the isomer **1a** (8*S*, 9*S*, 10*S*, 12*R*, and 14*R*) and 43 conformers of the enantiomer **1b** (8*R*, 9*R*,10*R*, 12*S*, and 14*S*). Those occurring conformers were then subjected to geometrical optimization and vibrational frequencies calculation using the DFT/B3LYP/6-31G(d,p) (IEFPCM, acetonitrile) level with the Gaussian 16 A.03 package (Gaussian Inc., Wallingford, CT, USA) [19]. The low-energy conformers over 0.5% Boltzmann population were chosen for ECD calculation at TD-DFT/CAM-B3LYP/6-31G(d,p) (IEFPCM, acetonitrile) level. ECD curves were Boltzmann averaged and extracted by SpecDis v.1.7 software with a half-band of 0.3 eV.

For NMR calculation, all 43 conformers of **1b** (8R, 9R,10R, 12S, and 14S) were further geometrically optimized using the DFT/B3LYP/6-31G(d,p) (IEFPCM, chloroform) level. They were then calculated for NMR shielding tensors at the IEFPCM/mpw1pw91/6-311G(d,p) level with chloroform as a solvent. The calculated NMR shielding tensors were averaged based on the Boltzmann populations, and the chemical shift values were calculated by the equation below (Equation (1)) where *δ*^x^ is the calculated NMR shift for nucleus x, and *σ*^0^ is the shielding tensor for the proton or carbon nuclei in tetramethylsilane calculated at the same condition.

*δ*^x^ = (*σ*^0^ − *σ*^x^)/(1 − *σ*^0^/10^6^) (1)

### 3.6. Antimicrobial Assay

Stock solutions of all compounds were prepared in DMSO. Two bacterial (*E. coli* UTI89 and *S. aureus* S25) [20,21] and two yeast strains (*C. krusei* CK1 and *C. albicans* CA01) have been used for this study. For antibacterial assays, the two strains *E. coli* UTI89 and *S. aureus* S25 were inoculated in LB liquid medium and incubated at 37 °C with agitation until the culture had an optical density (OD_600nm_) of 1.2. Each culture was diluted 12 times to have a final OD value of 0.1. Then, 100 µL from the diluted (OD of 0.1) bacterial culture and 0.5 μL of each stock solution was added to the corresponding wells (in triplicate) in a 96-well plate to a final concentration of 50 μM. For the antifungal assay, the two strains *C. krusei* CK1 and *C. albicans* CA01 were spread on Petri plates with YPD (yeast-extract peptone dextrose) agar. Later, an isolated colony was collected in a tube containing 5mL of RGM (RPMI 1640^®^ (21875, Invitrogen GIBCO^®^) + 34.56g/L MOPS) to have an OD of 0.05. The fungal culture and stock solutions of compounds were diluted and added to the wells as indicated for the antibacterial activity assay. To determine the potential inhibitory effect of extracts on fungal growth, absorbance at t = 0 h, t = 20 h (for antibacterial assay), and t = 48 h (for antifungal assay) were measured with the Biotek Synergy 2 Plate Reader at a wavelength of 600 nm and analyzed with the software Gen5 v3.08. Then, the differential OD was calculated (ODT48/20-ODT0) and compared to solvent control. A two-fold dilution concentration series of each compound that showed no growth (at 50 μM) was prepared to repeat the experiment to determine MIC/MBC. Ampicillin was used as positive control (MIC of 0.5 µg/mL for all bacteria and 1.0 µg/mL for all fungi).

### 3.7. Hemolytic and Cytotoxicity Assay

Each compound which showed antimicrobial activity at a concentration of 50 μM was selected for the cytotoxicity assay. Human erythrocytes (RBC), human monocytes (THP-1), and peripheral blood mononuclear cells (PBMC) were used for this study. THP-1 cells (TIB-202) were obtained from ATCC (https://www.atcc.org (accessed on 26 January 2025)) and activated with PMA (phorbol myristate acetate) (100 ng/mL) for 48 h. Cells were grown in RPMI 1640 medium supplemented with fetal bovine serum (FBS) 10% (10270-098, GIBCO^®^) and penicillin/streptomycin (1%) (15140-122 GIBCO^®^) at 37 °C under 5% CO_2_ atmosphere.

For the hemolytic assay, freshly obtained human erythrocytes (RBCs) from patients were washed twice with PBS (pH 7.4). This assay was performed at 10% hematocrit with two-fold serial dilutions from 200 μM of test compounds in a final volume of 100 μL, followed by incubation at 37 °C under 5% CO_2_ atmosphere for 30 min. Later, the plates were centrifugated at 3000*g* for 5 min and supernatant was transferred onto a new 96-well plate. Finally, RBC lysis was determined by the absorbance of the supernatant at 540 nm. Results were calculated with the following formula (Equation (2)):

(2) $$\%\;\mathrm{hemolysis}\;=\;100\;\times \;\frac{\mathrm{ODt}\;-\;\mathrm{OD}100}{\mathrm{OD}0\;-\;\mathrm{OD}100\;}$$

where ODt = the absorbance value of test compounds; OD100 = the absorbance value of the medium; and OD0 = the absorbance value of the wells treated with 1% Triton-X.

For the cytotoxicity assay, THP-1 and PBMC medium cultures were added into 96-well plates with 5 × 10^3^ cells per well. A two-fold dilution concentration series of each compound was added to the plates to final concentrations of 200 − 6.25 μM. AlamarBlue (DAL1025, ThermoFisher, Waltham, MA, USA), a ready-to-use resazurin-based solution, was used to measure cell viability after treatment with the extracts. A total of 10 µL was added to each well and the plate was incubated for 6–8 h at 37 °C. Fluorescence was then measured with the Biotek Synergy 2 Plate Reader using excitation at 530 nm and emission at 590 nm. The percentage of cell death was then calculated using the following formula (Equation (3)):

(3) $$\%\;\mathrm{Cellular}\;\mathrm{Death}\;=\;100\;\times \;\frac{\mathrm{ODt}\;-\;\mathrm{OD}100}{\mathrm{OD}0\;-\;\mathrm{OD}100}$$

where ODt = the fluorescence value of the test compound, OD100 = the fluorescence value for the culture medium, and OD0 = the fluorescence value for Triton 0.12%.

## 4. Conclusions

The marine fungus *Amesia nigricolor* MUT6601 has proven to be a promising source of bioactive compounds. In our study, a new bicyclic polyketide, amesilide (**1**), along with the previously reported metabolites, chamisides A (**2**), B (**3**), and E (**4**), chaetoconvosins B (**5**) and C (**6**), and chaetochromins A (**7**) and B (**8**), were isolated from the marine fungus *Amesia nigricolor* MUT6601. Notably, chaetochromins A (**7**) and B (**8**) exhibited significant antibacterial activity against *Staphylococcus aureus*, with minimal cytotoxicity, suggesting their potential for further development as therapeutic agents. These results highlight the potential of marine fungi as prolific sources of novel bioactive compounds, and lay the groundwork for future pharmacological development and optimization.

## Figures and Tables

**Table 1:** ^1^H (400 MHz) and ^13^C NMR (100 MHz) data (in CDCl_3_) of amesilide (**1**) and its calculated chemical shifts.

Position	1		Calculated Shifts	
	^1^H [*δ*, mult. (*J* in Hz)]	^13^C (*δ*)	^1^H (*δ*)	^13^C (*δ*)
1	-	172.0	-	172.6
2	5.81 d (15.5)	115.6	5.82	118.5
3	7.37 d (15.5)	152.9	7.68	161.2
4	-	131.9	-	140.4
5	6.27 s	144.7	6.68	154.8
6	-	135.4	-	144.9
7	5.40 d (9.0)	134.0	5.73	144.0
8	5.15 dd (9.0, 2.6)	37.5	6.05	41.9
9	1.81 br d (11.8)	50.9	1.83	56.8
10	2.28 m	44.5	2.27	49.0
11	-	213.8	-	223.4
12	2.42 dq (12.1, 6.1)	41.2	2.40	46.0
13	2.10 ddd (13.5, 5.1, 2.2); 1.60 td (13.5, 5.1)	41.3	2.09 1.59	44.2
14	2.83 br s	33.5	2.91	38.9
15	6.04 s	139.3	6.64	153.1
16	-	133.4	-	142.5
17	-	151.1	-	163.5
18	6.27 s	122.9	6.47	125.3
19	-	198.8	-	206.2
20	2.25 s	32.7	2.27	36.1
21	1.90 br s	13.9	1.83	15.5
22	2.00 br s	16.8	2.07	19.7
23	1.07 d (6.4)	11.5	1.00	13.5
24	0.99 d (6.4)	14.6	0.89	16.5
25	1.96 br s	20.1	2.01	22.4

## Data Availability

The original data presented in the study are included in the article/Supplementary Materials; further inquiries can be directed to the corresponding author.

## Supplementary Materials

The following supporting information can be downloaded at https://www.mdpi.com/article/10.3390/molecules30153169/s1, Figure S1. HRESIMS of **1**; Figure S2. IR spectrum of **1**; Figure S3. ^1^H NMR (400 MHz) spectrum of **1** in CDCl_3_; Figure S4. ^13^C NMR (100 MHz) spectrum of **1** in CDCl_3_; Figure S5. ^1^H-^1^H COSY spectrum of **1** in CDCl_3_; Figure S6. ^1^H-^13^C HSQC spectrum of **1** in CDCl_3_; Figure S7. ^1^H-^13^C HMBC spectrum of **1** in CDCl_3_; Figure S8. ^1^H-^1^H NOESY spectrum of **1** in CDCl_3_; Figure S9. HRESIMS of **2**; Figure S10. ^1^H NMR (400 MHz) spectrum of **2** in CDCl_3_; Figure S11. ^13^C NMR (100 MHz) spectrum of **2** in CDCl_3_; Figure S12. ^1^H-^1^H COSY spectrum of **2** in CDCl_3_; Figure S13. ^1^H-^13^C HSQC spectrum of **2** in CDCl_3_; Figure S14. ^1^H-^13^C HMBC spectrum of **2** in CDCl_3_; Figure S15. ^1^H-^1^H NOESY spectrum of **2** in CDCl_3_; Figure S16. HRESIMS of **3**; Figure S17. ^1^H NMR (400 MHz) spectrum of **3** in CD_3_OD; Figure S18. ^13^C NMR (100 MHz) spectrum of **3** in CD_3_OD; Figure S19. ^1^H-^1^H COSY spectrum of **3** in CD_3_OD; Figure S20. ^1^H-^13^C HSQC spectrum of **3** in CD_3_OD; Figure S21. ^1^H-^13^C HMBC spectrum of **3** in CD_3_OD; Figure S22. ^1^H-^1^H NOESY spectrum of **3** in CD_3_OD; Figure S23. HRESIMS of **4**; Figure S24. ^1^H NMR (400 MHz) spectrum of **4** in CDCl_3_; Figure S25. ^13^C NMR (100 MHz) spectrum of **4** in CDCl_3_; Figure S26. ^1^H-^1^H COSY spectrum of **4** in CDCl_3_; Figure S27. ^1^H-^13^C HSQC spectrum of **4** in CDCl_3_; Figure S28. ^1^H-^13^C HMBC spectrum of **4** in CDCl_3_; Figure S29. ^1^H-^1^H NOESY spectrum of **4** in CDCl_3_; Figure S30. HRESIMS of **5**; Figure S31. ^1^H NMR (400 MHz) spectrum of **5** in CDCl_3_; Figure S32. ^13^C NMR (100 MHz) spectrum of **5** in CDCl_3_; Figure S33. ^1^H-^1^H COSY spectrum of **5** in CDCl_3_; Figure S34. ^1^H-^13^C HSQC spectrum of **5** in CDCl_3_; Figure S35. ^1^H-^13^C HMBC spectrum of **5** in CDCl_3_; Figure S36. ^1^H-^1^H NOESY spectrum of **5** in CDCl_3_; Figure S37. HRESIMS of **6**; Figure S38. ^1^H NMR (400 MHz) spectrum of **6** in CDCl_3_; Figure S39. ^13^C NMR (100 MHz) spectrum of **6** in CDCl_3_; Figure S40. ^1^H-^1^H COSY spectrum of **6** in CDCl_3_; Figure S41. ^1^H-^13^C HSQC spectrum of **6** in CDCl_3_; Figure S42. ^1^H-^13^C HMBC spectrum of **6** in CDCl_3_; Figure S43. ^1^H-^1^H NOESY spectrum of **6** in CDCl_3_; Figure S44. HRESIMS of **7**; Figure S45. ^1^H NMR (400 MHz) spectrum of **7** in CDCl_3_; Figure S46. ^13^C NMR (100 MHz) spectrum of **7** in CDCl_3_; Figure S47. ^1^H-^1^H COSY spectrum of **7** in CDCl_3_; Figure S48. ^1^H-^13^C HSQC spectrum of **7** in CDCl_3_; Figure S49. ^1^H-^13^C HMBC spectrum of **7** in CDCl_3_; Figure S50. ^1^H-^1^H NOESY spectrum of **6** in CDCl_3_; Figure S51. HRESIMS of **8**; Figure S52. ^1^H NMR (400 MHz) spectrum of **8** in CDCl_3_; Figure S53. ^13^C NMR (100 MHz) spectrum of **8** in CDCl_3_; Figure S54. ^1^H-^13^C HSQC spectrum of **8** in CDCl_3_; Figure S55. ^1^H-^13^C HMBC spectrum of **8** in CDCl_3_; Figure S56. ^1^H-^1^H COSY spectrum of **8** in CDCl_3_; Figure S57. ^1^H-^1^H NOESY spectrum of **8** in CDCl_3_; Table S1: Cytotoxic activities of compounds **7**–**8** against RBC, THP-1, and PBMC cells. Table S2: Gibbs free energy and Boltzmann population of conformers of isomer **1a**—8*R*, 9*R*, 10*R*, 12*S*, and 14*S*; Table S3. Gibbs free energy and Boltzmann population of conformers of isomer **1b**—8*S*, 9*S*, 10*S,* 12*R*, and 14*R*; Table S4. Coordinates (Ångstroms) for conformer **1a-1**; Table S5. Coordinates (Ångstroms) for conformer **1a-2**; Table S6. Coordinates (Ångstroms) for conformer **1a-3**; Table S7. Coordinates (Ångstroms) for conformer **1a-4**; Table S8. Coordinates (Ångstroms) for conformer **1a-5**; Table S9. Coordinates (Ångstroms) for conformer **1a-6**; Table S10. Coordinates (Ångstroms) for conformer **1a-7**; Table S11. Coordinates (Ångstroms) for conformer **1a-8**; Table S12. Coordinates (Ångstroms) for conformer **1a-17**; Table S13. Coordinates (Ångstroms) for conformer **1a-37**; Table S14. Coordinates (Ångstroms) for conformer **1a-39**; Table S15. Coordinates (Ångstroms) for conformer **1b-1**; Table S16. Coordinates (Ångstroms) for conformer **1b-2**; Table S17. Coordinates (Ångstroms) for conformer **1b-3**; Table S18. Coordinates (Ångstroms) for conformer **1b-4**; Table S19. Coordinates (Ångstroms) for conformer **1b-5**; Table S20. Coordinates (Ångstroms) for conformer **1b-6**; Table S21. Coordinates (Ångstroms) for conformer **1b-7**; Table S22. Coordinates (Ångstroms) for conformer **1b-8**; Table S23. Coordinates (Ångstroms) for conformer **1b-17**; Table S24. Coordinates (Ångstroms) for conformer **1b-25**; Table S25. Coordinates (Ångstroms) for conformer **1b-39.**
