# Supplementary material for: Amesilide, a New Bicyclic Polyketide from the Marine Fungus Amesia nigricolor MUT6601
Source: Molecules. 2025 Jul 29;30(15):3169. doi: 10.3390/molecules30153169 (PMC12348691; doi:10.3390/molecules30153169)
Supplement: Supplementary file 1 [file molecules-30-03169-s001.zip › molecules-3387807-supplementary.pdf]

Supporting Information

Amesilide, a new bicyclic polyketide from the marine fungus *Amesia nigricolor* MUT6601

Giang Nam Pham [1, †], Matteo Florio Furno [2], Juan A. Garcia-Sanchez [3], Patrick Munro [3], Fatouma Mohamed Abdoul-Latif [4], Laurent Boyer [3], Giovanna Cristina Varese [2], and Mohamed Mehiri [1,*]

Contents

Figure S1. HRESIMS of 1 5
Figure S2. IR spectrum of 1 6
Figure S3. ^{1}H NMR (400 MHz) spectrum of 1 in $CDCl_3$ 7
Figure S4. ^{13}C NMR (100 MHz) spectrum of 1 in $CDCl_3$ 8
Figure S5. ^{1}H-^{1}H COSY spectrum of 1 in $CDCl_3$ 9
Figure S6. ^{1}H-^{13}C HSQC spectrum of 1 in $CDCl_3$ 10
Figure S7. ^{1}H-^{13}C HMBC spectrum of 1 in $CDCl_3$ 11
Figure S8. ^{1}H-^{1}H NOESY spectrum of 1 in $CDCl_3$ 12
Figure S9. HRESIMS of 2 15
Figure S10. ^{1}H NMR (400 MHz) spectrum of 2 in $CDCl_3$ 16
Figure S11. ^{13}C NMR (100 MHz) spectrum of 2 in $CDCl_3$ 17
Figure S12. ^{1}H-^{1}H COSY spectrum of 2 in $CDCl_3$ 18
Figure S13. ^{1}H-^{13}C HSQC spectrum of 2 in $CDCl_3$ 19
Figure S14. ^{1}H-^{13}C HMBC spectrum of 2 in $CDCl_3$ 20
Figure S15. ^{1}H-^{1}H NOESY spectrum of 2 in $CDCl_3$ 21
Figure S16. HRESIMS of 3 22
Figure S17. ^{1}H NMR (400 MHz) spectrum of 3 in CD_3OD 23
Figure S18. ^{13}C NMR (100 MHz) spectrum of 3 in CD_3OD 24
Figure S19. ^{1}H-^{1}H COSY spectrum of 3 in CD_3OD 25
Figure S20. ^{1}H-^{13}C HSQC spectrum of 3 in CD_3OD 26
Figure S21. ^{1}H-^{13}C HMBC spectrum of 3 in CD_3OD 27
Figure S22. ^{1}H-^{1}H NOESY spectrum of 3 in CD_3OD 28
Figure S23. HRESIMS of 4 29

Figure S24. ^{1}H NMR (400 MHz) spectrum of 4 in $CDCl_3$ 30
Figure S25. ^{13}C NMR (100 MHz) spectrum of 4 in $CDCl_3$ 31
Figure S26. ^{1}H-^{1}H COSY spectrum of 4 in $CDCl_3$ 32
Figure S27. ^{1}H-^{13}C HSQC spectrum of 4 in $CDCl_3$ 33
Figure S28. ^{1}H-^{13}C HMBC spectrum of 4 in $CDCl_3$ 34
Figure S29. ^{1}H-^{1}H NOESY spectrum of 4 in $CDCl_3$ 35
Figure S30. HRESIMS of 5 36
Figure S31. ^{1}H NMR (400 MHz) spectrum of 5 in $CDCl_3$ 37
Figure S32. ^{13}C NMR (100 MHz) spectrum of 5 in $CDCl_3$ 38
Figure S33. ^{1}H-^{1}H COSY spectrum of 5 in $CDCl_3$ 39
Figure S34. ^{1}H-^{13}C HSQC spectrum of 5 in $CDCl_3$ 40
Figure S35. ^{1}H-^{13}C HMBC spectrum of 5 in $CDCl_3$ 41
Figure S36. ^{1}H-^{1}H NOESY spectrum of 5 in $CDCl_3$ 42
Figure S37. HRESIMS of 6 43
Figure S38. ^{1}H NMR (400 MHz) spectrum of 6 in $CDCl_3$ 44
Figure S39. ^{13}C NMR (100 MHz) spectrum of 6 in $CDCl_3$ 45
Figure S40. ^{1}H-^{1}H COSY spectrum of 6 in $CDCl_3$ 46
Figure S41. ^{1}H-^{13}C HSQC spectrum of 6 in $CDCl_3$ 47
Figure S42. ^{1}H-^{13}C HMBC spectrum of 6 in $CDCl_3$ 48
Figure S43. ^{1}H-^{1}H NOESY spectrum of 6 in $CDCl_3$ 49
Figure S44. HRESIMS of 7 50
Figure S45. ^{1}H NMR (400 MHz) spectrum of 7 in $CDCl_3$ 51
Figure S46. ^{13}C NMR (100 MHz) spectrum of 7 in $CDCl_3$ 52
Figure S47. ^{1}H-^{1}H COSY spectrum of 7 in $CDCl_3$ 53
Figure S48. ^{1}H-^{13}C HSQC spectrum of 7 in $CDCl_3$ 54
Figure S49. ^{1}H-^{13}C HMBC spectrum of 7 in $CDCl_3$ 55
Figure S50. ^{1}H-^{1}H NOESY spectrum of 7 in $CDCl_3$ 56
Figure S51. HRESIMS of 8 57
Figure S52. ^{1}H NMR (400 MHz) spectrum of 8 in $CDCl_3$ 58
Figure S53. ^{13}C NMR (100 MHz) spectrum of 8 in $CDCl_3$ 59

Figure S54. ^{1}H-^{1}H COSY spectrum of 8 in $CDCl_3$.. 60
Figure S55. ^{1}H-^{13}C HSQC spectrum of 8 in $CDCl_3$.. 61
Figure S56. ^{1}H-^{13}C HMBC spectrum of 8 in $CDCl_3$ 62
Figure S57. ^{1}H-^{1}H NOESY spectrum of 8 in $CDCl_3$.. 63
Table S1. Antimicrobial and cytotoxic activities of compounds 7-8... 64
Table S2. Gibbs Free Energy and Boltzmann Population of occurring conformers of isomer 1a – 8*R*, 9*R*, 10*R*, 12*S*, 14*S* 65
Table S3. Gibbs Free Energy and Boltzmann Population of occurring conformers of isomer 1b – 8*S*, 9*S*, 10*S*, 12*R*, 14*R*... 67
Table S4. Coordinates (Ångstroms) for conformer 1a-1 69
Table S5. Coordinates (Ångstroms) for conformer 1a-2 71
Table S6. Coordinates (Ångstroms) for conformer 1a-3 73
Table S7. Coordinates (Ångstroms) for conformer 1a-4 75
Table S8. Coordinates (Ångstroms) for conformer 1a-5 77
Table S9. Coordinates (Ångstroms) for conformer 1a-6 79
Table S10. Coordinates (Ångstroms) for conformer 1a-7 81
Table S11. Coordinates (Ångstroms) for conformer 1a-8 83
Table S12. Coordinates (Ångstroms) for conformer 1a-17 85
Table S13. Coordinates (Ångstroms) for conformer 1a-37 87
Table S14. Coordinates (Ångstroms) for conformer 1a-39 89
Table S15. Coordinates (Ångstroms) for conformer 1b-1 91
Table S16. Coordinates (Ångstroms) for conformer 1b-2 93
Table S17. Coordinates (Ångstroms) for conformer 1b-3 95
Table S18. Coordinates (Ångstroms) for conformer 1b-4 97
Table S19. Coordinates (Ångstroms) for conformer 1b-5 99
Table S20. Coordinates (Ångstroms) for conformer 1b-6 101
Table S21. Coordinates (Ångstroms) for conformer 1b-7 103
Table S22. Coordinates (Ångstroms) for conformer 1b-8 105
Table S23. Coordinates (Ångstroms) for conformer 1b-17 107
Table S24. Coordinates (Ångstroms) for conformer 1b-25 109
Table S25. Coordinates (Ångstroms) for conformer 1b-39 111

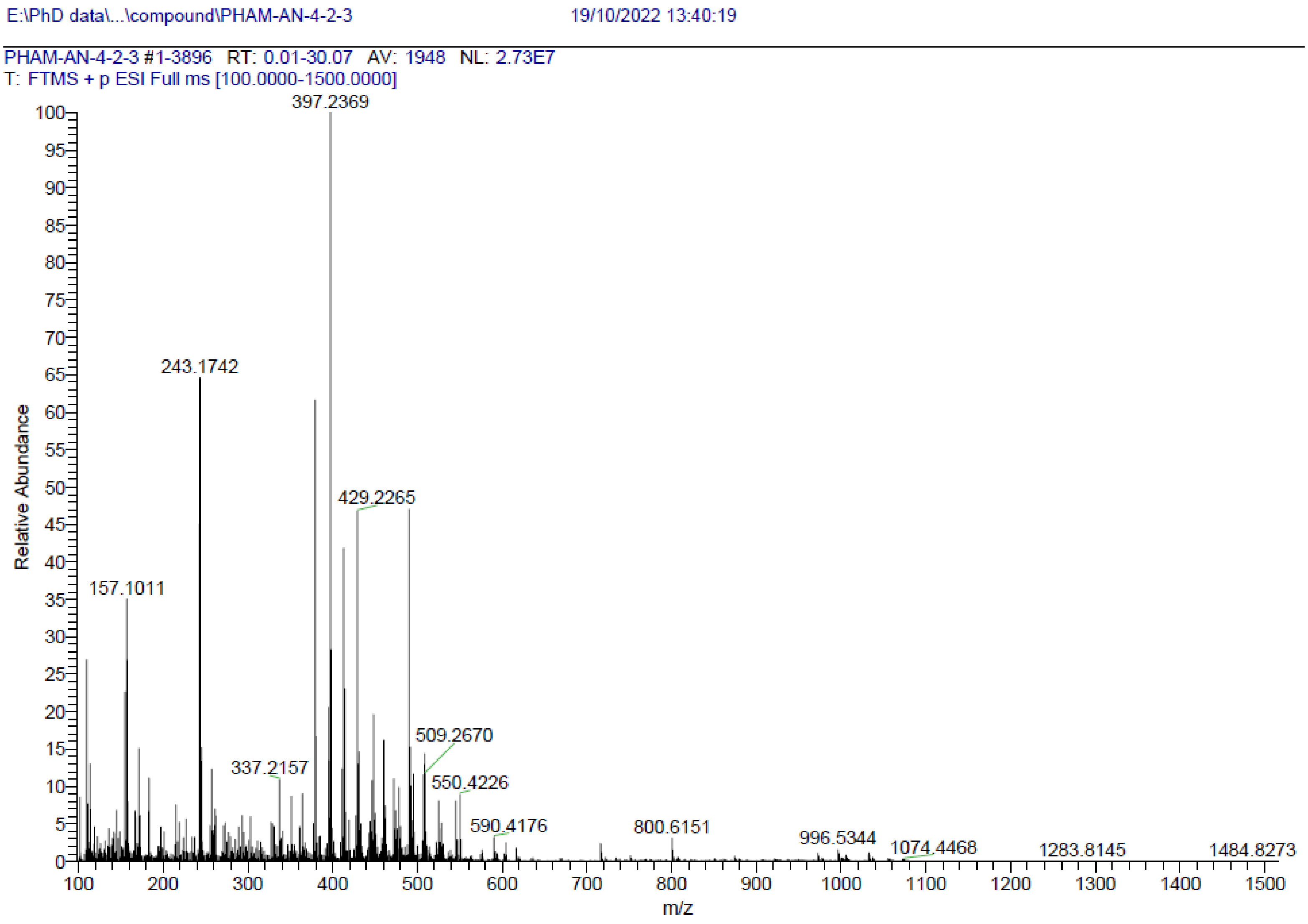

Figure S1. HRESIMS of **1**

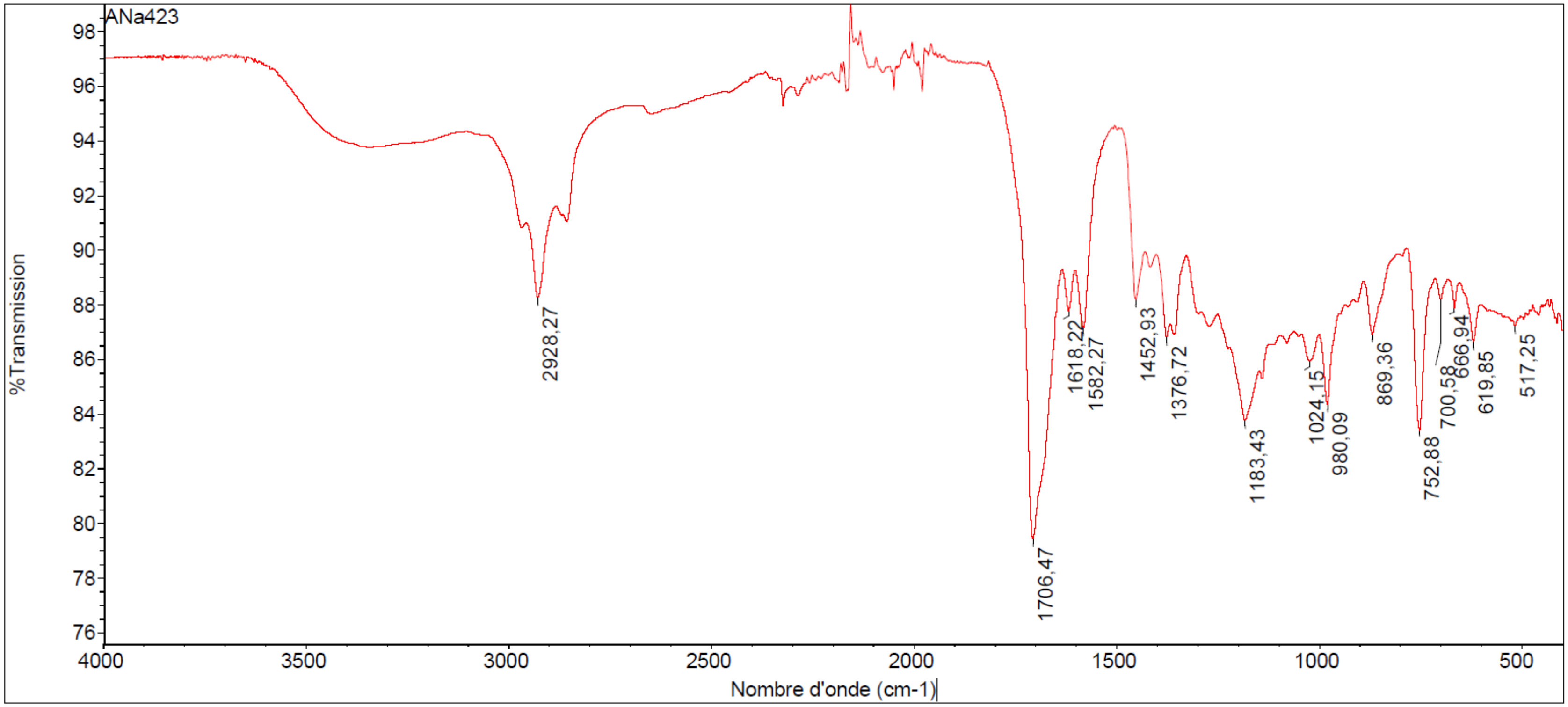

Figure S2. IR spectrum of **1**

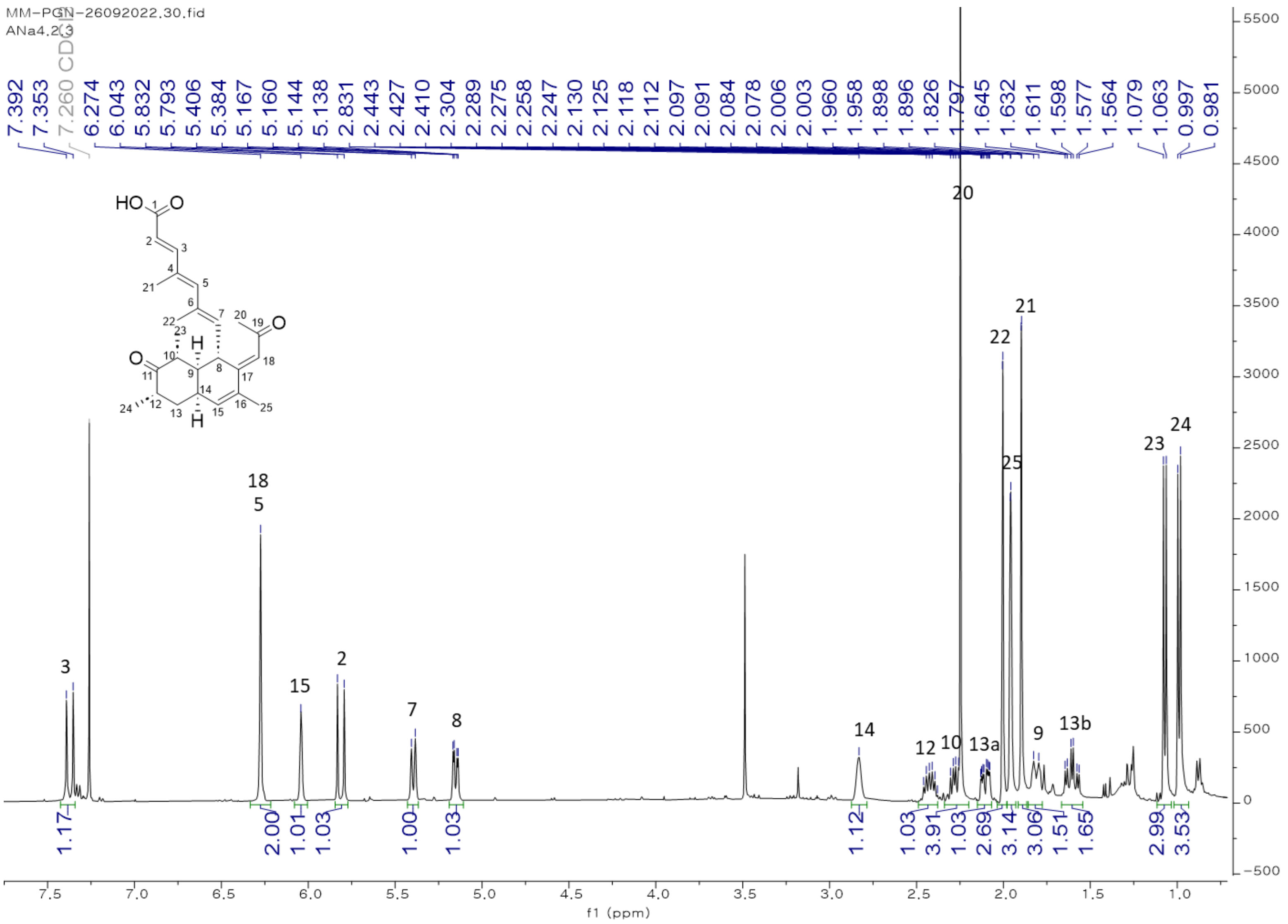

Figure S3. ^{1}H NMR (400 MHz) spectrum of **1** in $CDCl_3$

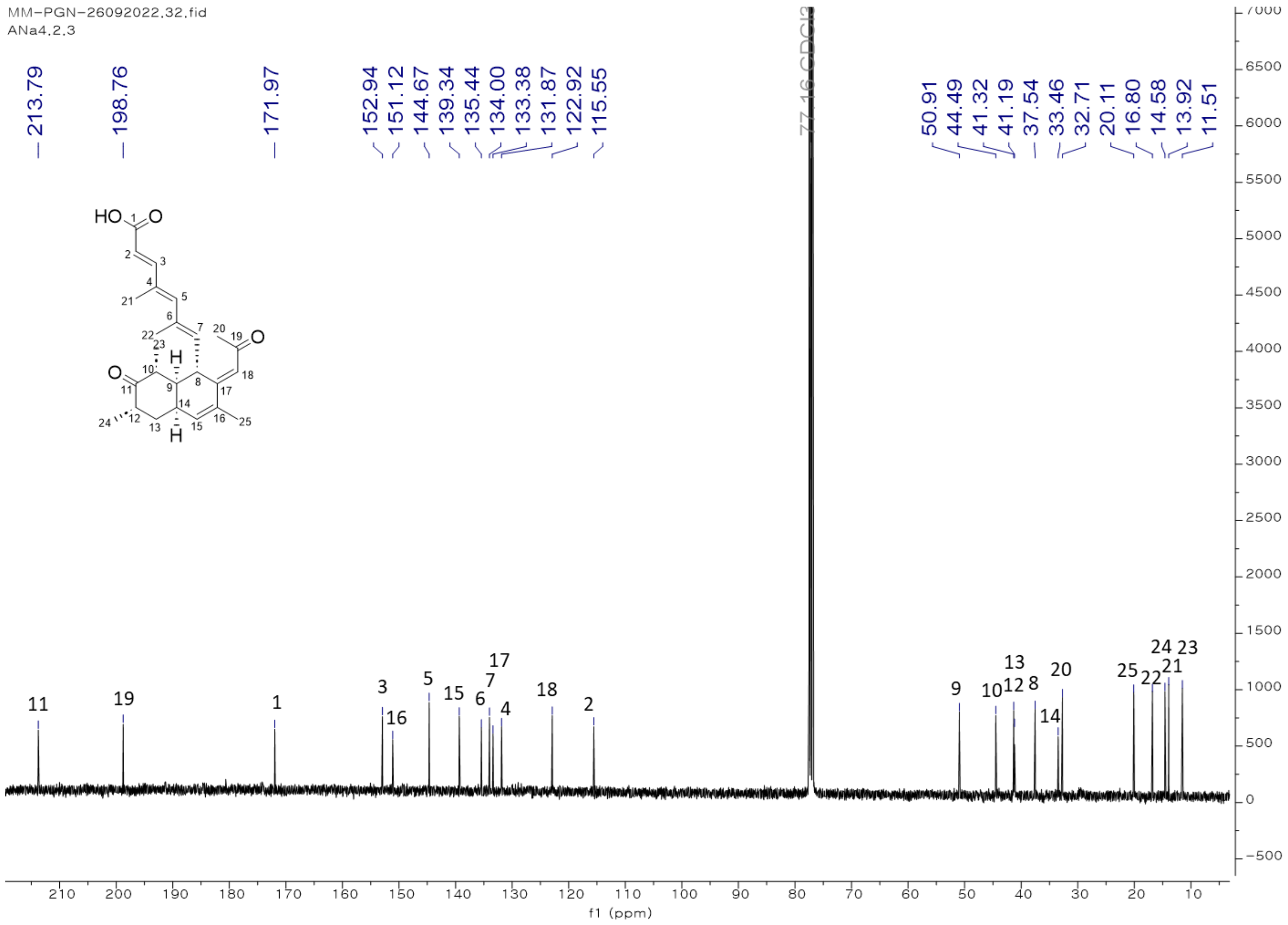

Figure S4. ^{13}C NMR (100 MHz) spectrum of **1** in $CDCl_3$

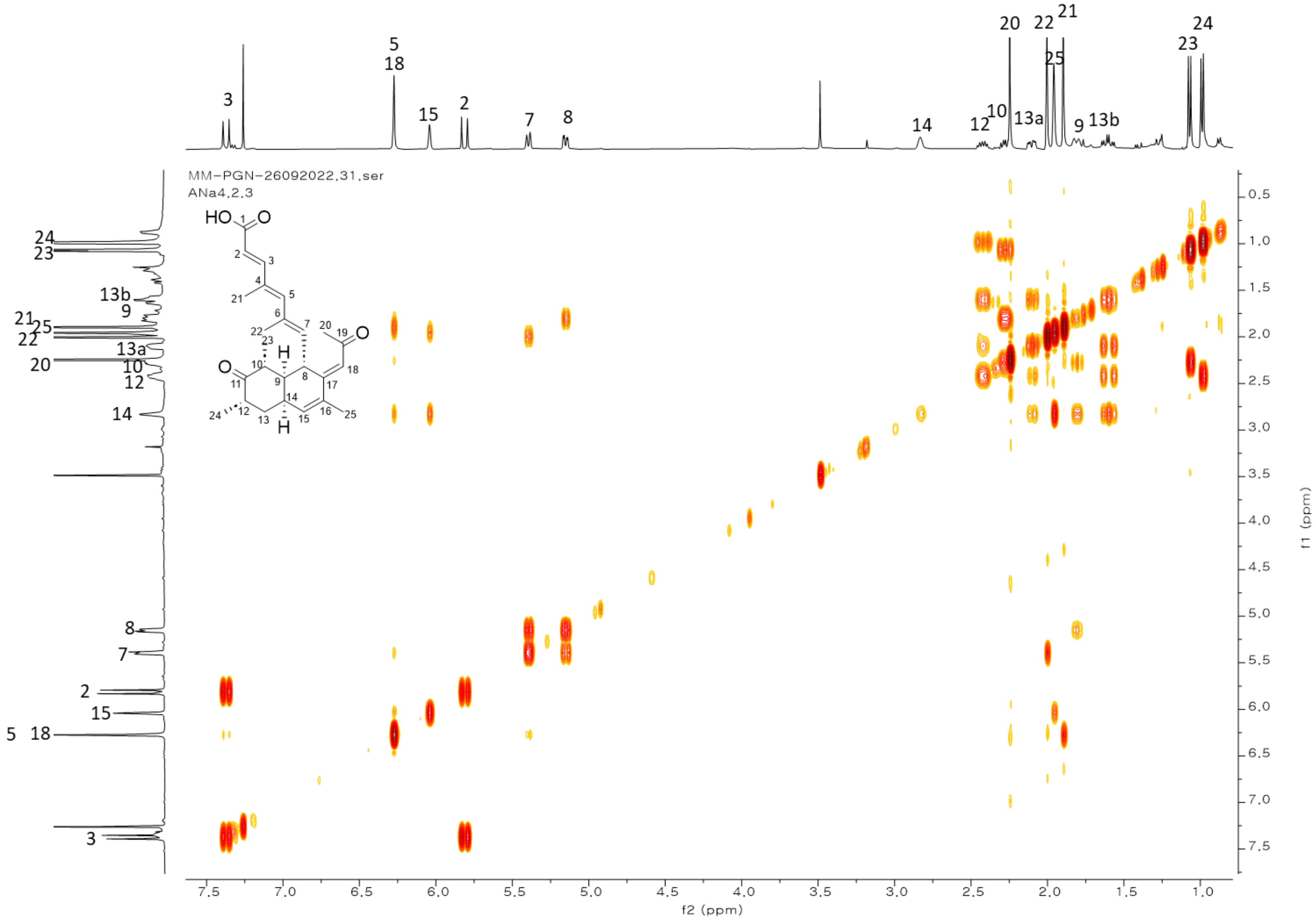

Figure S5. ^{1}H-^{1}H COSY spectrum of **1** in $CDCl_3$

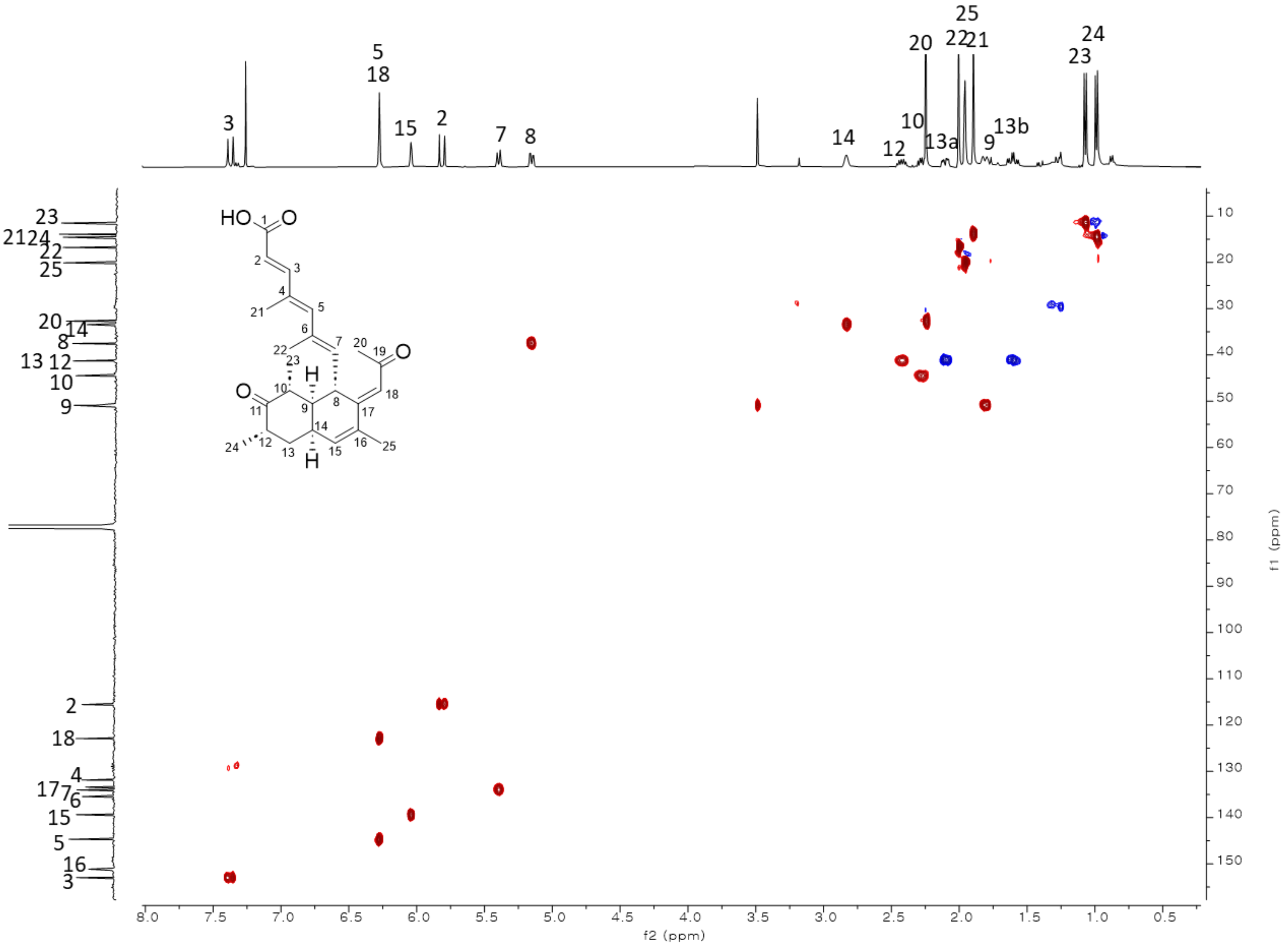

Figure S6. ^{1}H-^{13}C HSQC spectrum of **1** in $CDCl_3$

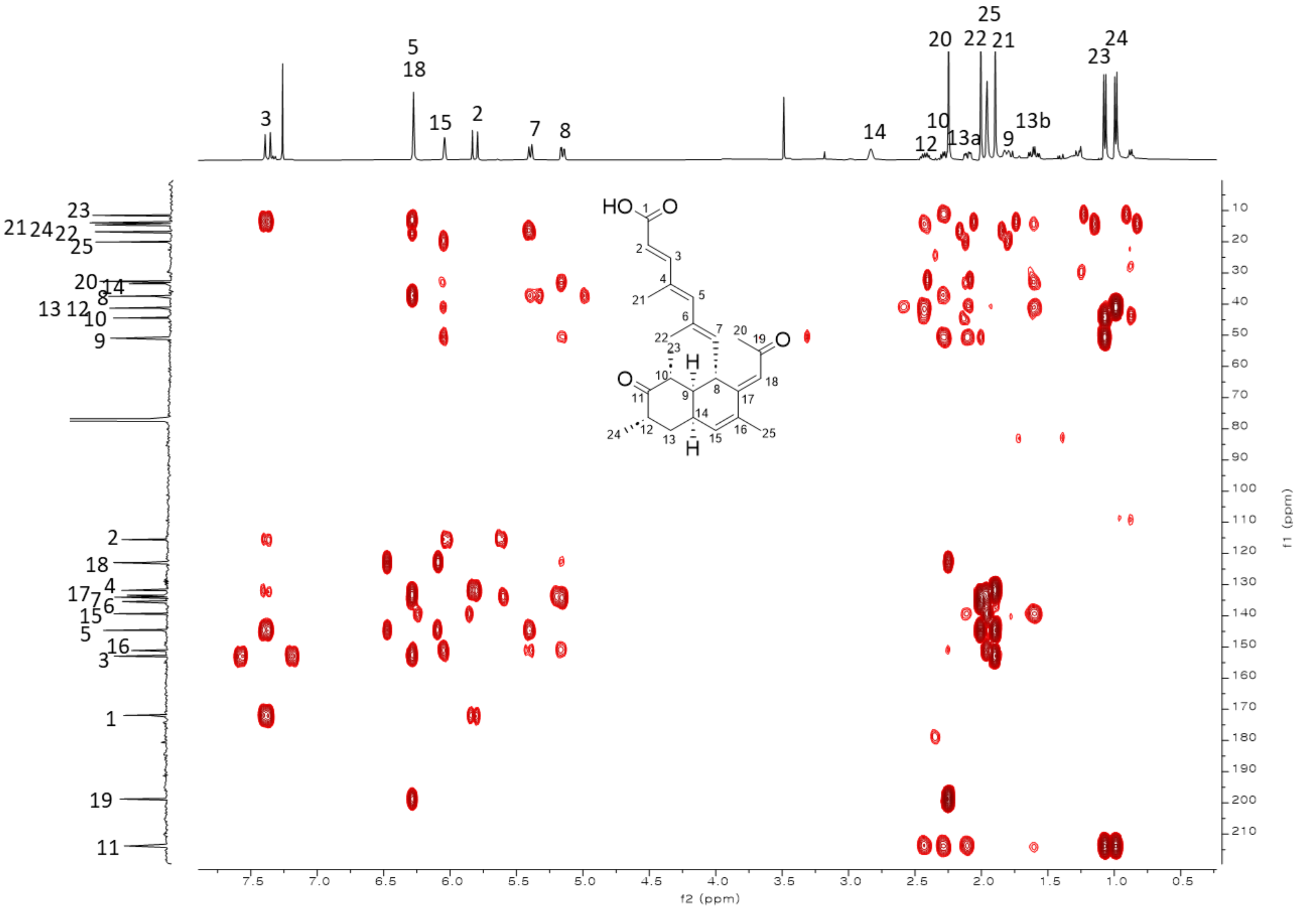

Figure S7. ^{1}H-^{13}C HMBC spectrum of **1** in $CDCl_3$

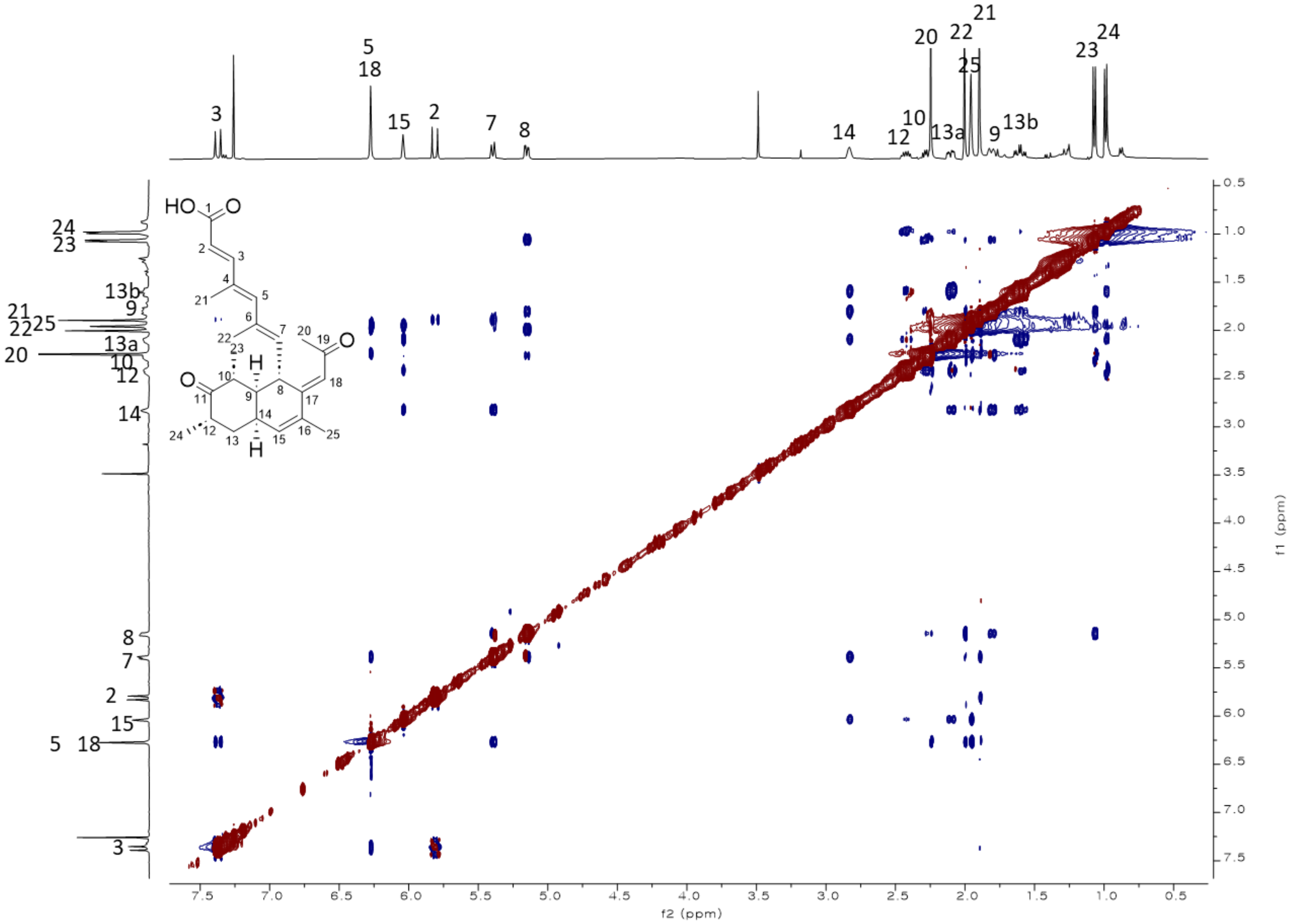

Figure S8. ^{1}H-^{1}H NOESY spectrum of **1** in $CDCl_3$

Chamiside A (**2**): Amorphous powder. $[\alpha]_D^{20}$ +67.7 (*c* 0.6, $CHCl_3$). Molecular formula: $C_{30}H_{37}NO_6$. ^{1}H NMR (400 MHz, $CDCl_3$) δ_H 7.32-7.24 (3H, overlap, H-3′-H-5′), 7.22-7.19 (2H, overlap, H-2′, H-6′), 5.94 (1H, br s, H-8), 4.01 (1H, d, *J* = 11.9 Hz, H-4), 3.19 (1H, dd, *J* = 11.9, 4.3 Hz, H-5), 3.10 (3H, s, H-25), 3.02 (1H, dd, *J* = 13.4, 8.0 Hz, H-13), 2.71 (1H, m, H-20), 2.69 (1H, dd, *J* = 18.7, 7.9 Hz, H-14a), 2.32 (1H, dd, *J* = 18.7, 13.5 Hz, H-14b), 2.28 (1H, m, H-6), 2.15 (1H, m, H-18), 1.74 (3H, s, H-22), 1.73 (3H, s, H-12), 1.70-1.63 (3H, overlap, H-17, H-19a), 1.24 (1H, m, H-19b), 1.22 (3H, d, *J* = 6.6 Hz, H-24), 0.96 (3H, d, *J* = 6.5 Hz, H-23), 0.44 (3H, d, *J* = 7.6 Hz, H-11). ^{13}C NMR (100 MHz, $CDCl_3$) δ_C 210.9 (C-15), 175.8 (C-21), 174.5 (C-3), 172.2 (C-1), 140.8 (C-1′), 138.3 (C-7), 128.9 (C-2′, C-3′, C5′, C-6′), 127.7 (C-4′), 123.9 (C-8), 84.0 (C-16), 78.4 (C-9), 66.2 (C-10), 56.4 (C-13), 47.9 (C-4), 45.1 (C-17), 40.7 (C-19), 40.2 (C-14), 39.8 (C-20), 38.4 (C-5), 33.3 (C-6), 29.9 (C-18), 27.5 (C-25), 24.4 (C-22), 23.7 (C-23), 21.5 (C-12), 19.2 (C-24), 16.7 (C-11). HRESIMS *m/z* 506.2547 [M - H]$^-$ (calcd for $C_{30}H_{36}NO_6^-$ 506.2548).

Chamiside B (**3**): Amorphous powder. $[\alpha]_D^{20}$ +11.0 (*c* 0.4, $CHCl_3$). Molecular formula: $C_{29}H_{37}NO_5$. ^{1}H NMR (400 MHz, CD_3OD) δ_H 7.36-7.26 (5H, overlap, H-2′-H-6′), 5.82 (1H, s, H-7), 4.69 (1H, d, *J* = 7.7 Hz, H-10), 3.45 (1H, br d, *J* = 7.7 Hz, H-3), 3.43 (1H, dd, *J* = 11.8, 7.7 Hz, H-13), 3.26 (1H, d, *J* = 7.8 Hz, H-20), 2.91-2.86 (2H, overlap, H-4, H-8), 2.53 (1H, m, H-5), 1.96-1.83 (3H, overlap, H-15a, H-16, H-18), 1.75 (3H, s, H-12), 1.62 (1H, m, H-17a), 1.30 (1H, m, H-15b), 1.27 (3H, s, H-22), 1.12 (3H, d, *J* = 7.3 Hz, H-11), 1.00 (3H, d, *J* = 6.7 Hz, H-24), 0.89 (3H, d, *J* = 6.8 Hz, H-23), 0.88 (1H, m, H-17b). ^{13}C NMR (100 MHz, CD_3OD) δ_C 213.0 (C-21), 176.5 (C-1), 143.2 (C-1′), 141.6 (C-6), 129.5 (C-3′, C-5′), 128.9 (C-4′), 128.2 (C-2′, C-6′), 126.0 (C-7), 111.0 (C-19), 81.9 (C-14), 77.9 (C-10), 72.1 (C-9), 59.7 (C-3), 58.8 (C-20), 55.4 (C-13), 53.6 (C-15), 45.3 (C-4), 45.2 (C-17), 44.6 (C-18), 44.4 (C-8), 37.0 (C-5), 30.2 (C-16), 25.9 (C-22), 24.5 (C-23), 19.9 (C-12), 16.9 (C-24), 14.0 (C-11). HRESIMS *m/z* 480.2741 [M + H]+ (calcd for $C_{29}H_{38}NO_5^+$ 480.2745).

Chamiside E (**4**): Amorphous powder. $[\alpha]_D^{20}$ +47.0 (*c* 0.1, $CHCl_3$). Molecular formula: $C_{30}H_{35}NO_5$. ^{1}H NMR (400 MHz, $CDCl_3$) δ_H 7.29-7.23 (3H, overlap, H-3′-H-5′), 6.96 (2H, br d, *J* = 7.1 Hz, H-2′, H-6′), 5.47 (1H, br s, H-8), 5.24 (1H, d, *J* = 2.1 Hz, H-22a), 4.93 (1H, br s, H-22b), 4.23 (1H, br s, H-4), 3.68 (1H, dd, *J* = 12.5, 10.6 Hz, H-13), 3.39 (1H, d, *J* = 10.5 Hz, H-20), 3.14 (3H, s, H-25), 3.01 (1H, dd, *J* = 8.7, 2.1 Hz, H-5), 2.94 (1H, br d, *J* = 12.7 Hz, H-9), 2.76 (1H, m, H-6), 2.47 (1H, m, H-18), 2.36 (1H, d, *J* = 13.7 Hz, H-15a), 2.03 (1H, dd, *J* = 13.0, 12.6 Hz, H-17a), 1.97 (1H, d, *J* = 13.7 Hz, H-15b), 1.73 (3H, s, H-12), 1.51 (1H, dd, *J* = 12.6, 6.2 Hz, H-17b), 1.36 (3H, d, *J* = 7.5 Hz, H-11), 1.34 (3H, s, H-23), 1.05 (3H, d, *J* = 7.5 Hz, H-24). ^{13}C NMR (100 MHz, $CDCl_3$) δ_C 213.2 (C-21), 172.3 (C-3), 171.4 (C-1), 144.8 (C-14), 143.7 (C-7), 137.7 (C-1′), 128.9 (C-3′, C-5′), 127.5 (C-4′), 127.4 (C-2′, C-6′), 121.1 (C-8), 120.3 (C-22), 111.0 (C-19), 82.8 (C-16), 63.1 (C-10), 56.3 (C-20), 50.4 (C-9), 48.4 (C-15), 47.6 (C-4), 45.9 (C-18), 44.8 (C-13), 42.1 (C-5), 40.7 (C-17), 30.9 (C-6), 28.8 (C-23), 27.4 (C-25), 19.4 (C-12), 15.4 (C-24), 14.5 (C-11). HRESIMS *m/z* 490.2580 [M + H]+ (calcd for $C_{30}H_{36}NO_5^+$ 490.2588).

Chaetoconvosin B (**5**): Amorphous powder. $[\alpha]_D^{20}$ +22.0 (*c* 0.3, $CHCl_3$). Molecular formula: $C_{30}H_{37}NO_6$. ^{1}H NMR (400 MHz, $CDCl_3$) δ_H 7.32-7.24 (5H, overlap, H-2′-H-6′), 6.25 (1H, s, H-8), 3.92 (1H, d, , *J* = 11.5 Hz, H-4), 3.41 (1H, d, *J* = 9.2 Hz, H-20), 3.24 (1H, dd, *J* = 11.4, 5.2 Hz, H-5), 3.08 (3H, s, H-25), 2.85 (1H, d, *J* = 9.2 Hz, H-13), 2.44 (1H, m, H-6), 1.92-1.82 (2H, overlap, H-16, H-18), 1.76 (1H, m, H-15a), 1.72 (3H, s, H-12), 1.65 (1H, m, H-17a), 1.62 (3H, s, H-22), 1.20 (1H, dd, *J* = 14.2, 11.9 Hz, H-15b), 1.01 (3H, d, *J* = 6.7 Hz, H-24), 0.85 (3H, d, *J* = 6.8 Hz, H-23), 0.81 (1H, m, H-17b), 0.53 (1H, d, *J* = 7.6 Hz, H-11). ^{13}C NMR (100 MHz, $CDCl_3$) δ_C 205.8 (C-21), 174.7 (C-3), 172.2 (C-1), 143.2 (C-7), 141.3 (C-1′), 129.1 (C-2′, C-3′, C5′, C-6′), 127.6 (C-4′), 123.5 (C-8), 109.3 (C-19), 79.9 (C-14), 77.2 (C-9), 67.2 (C-10), 63.3 (C-13), 56.1 (C-20), 55.1 (C-15), 48.9 (C-4), 44.3 (C-17), 42.9 (C-18), 36.7 (C-5), 33.0 (C-6), 28.4 (C-16), 27.9 (C-25), 24.5 (C-22), 24.1 (C-23), 22.4 (C-12), 16.7 (C-11), 16.2 (C-24). HRESIMS *m/z* 508.2684 [M + H]+ (calcd for $C_{30}H_{38}NO_6^+$ 508.2694).

Chaetoconvosin C (**6**): Amorphous powder. $[\alpha]_D^{20}$ +78.0 (*c* 0.3, $CHCl_3$). Molecular formula: $C_{29}H_{35}NO_6$. ^{1}H NMR (400 MHz, $CDCl_3$) δ_H 7.36-7.26 (5H, overlap, H-2′-H-6′), 6.28 (1H, s, H-8), 3.91 (1H, d, *J* = 11.7 Hz, H-4), 3.42 (1H, d, *J* = 9.2 Hz, H-20), 3.32 (1H, dd, *J* = 11.6, 5.1 Hz, H-5), 2.95 (1H, d, *J* = 9.2 Hz, H-13), 2.49 (1H, m, H-6), 1.91-1.76 (4H, overlap, H-15a, H-16, H-17a, H-18), 1.74 (3H, s, H-12), 1.67 (3H, s, H-22), 1.21 (1H, m, H-15b), 1.01 (3H, d, *J* = 6.7 Hz, H-24), 0.87 (3H, d, *J* = 6.8 Hz, H-23), 0.84 (1H, m, H-17b), 0.51 (1H, d, *J* = 7.6 Hz, H-11). ^{13}C NMR (100 MHz, $CDCl_3$) δ_C 205.4

(C-21), 173.7 (C-3), 171.6 (C-1), 143.3 (C-7), 140.2 (C-1′), 129.1 (C-2′, C-3′, C5′, C-6′), 128.0 (C-4′), 123.3 (C-8), 109.4 (C-19), 79.9 (C-14), 77.2 (C-9), 67.8 (C-10), 63.5 (C-13), 56.5 (C-20), 55.1 (C-15), 48.4 (C-4), 44.3 (C-17), 43.0 (C-18), 38.1 (C-5), 33.0 (C-6), 28.4 (C-16), 24.6 (C-22), 24.1 (C-23), 22.4 (C-12), 16.8 (C-11), 16.2 (C-24). HRESIMS *m/z* 492.2388 [M - H]$^-$ (calcd for $C_{29}H_{34}NO_6^-$ 492.2391).

Chaetochromin A (**7**): Yellow powder. $[\alpha]_D^{20}$ +490.5 (*c* 0.4, $CHCl_3$). Molecular formula: $C_{30}H_{26}O_{10}$. ^{1}H NMR (400 MHz, $CDCl_3$) δ_H 6.52 (2H, s, H-7, H-7′), 5.9 4 (2H, s, H-10, H-10′), 4.14 (2H, m, H-2, H-2′), 2.63 (2H, dq, *J* = 10.8, 7.0 Hz, H-3, H-3′), 1.42 (6H, d, *J* = 6.2 Hz, H-11, H-11′), 1.24 (6H, d, *J* = 6.7 Hz, H-12, H-12′). ^{13}C NMR (100 MHz, $CDCl_3$) δ_C 200.9 (C-4, C-4′), 164.7 (C-5, C-5′), 161.2 (C-6, C-6′), 159.9 (C-8, C-8′), 156.4 (C-10, C-10a′), 142.1 (C-9a, C-9a′), 102.2 (C-4a, C-4a′), 101.7 (C-9, C-9′), 99.7 (C-7, C-7′), 99.4 (C-10, C-10′), 78.5 (C-2, C-2′), 46.3 (C-3, C-3′), 19.8 (C-11, C-11′), 10.1 (C-12, C-12′). HRESIMS *m/z* 547.1591 [M + H]+ (calcd for $C_{30}H_{27}O_{10}^+$ 547.1599).

Chaetochromin B (**8**): Yellow powder. $[\alpha]_D^{20}$ +160.0 (*c* 0.2, $CHCl_3$). Molecular formula: $C_{30}H_{26}O_{10}$. ^{1}H NMR (400 MHz, $CDCl_3$) δ_H ^{1}H NMR (400 MHz, $CDCl_3$) δ_H 6.54 , 6.53 (each 1H, s, H-7, H-7′), 5.96, 5.95 (each 1H, s, H-10, H-10′), 4.52 (1H, m, H-2′), 4.16 (1H, dq, *J* = 12.6, 6.3 Hz H-2), 2.67-2.61 (2H, overlap, H-3, H-3′), 1.43 (3H, d, , *J* = 6.2 Hz, H-11′), 1.32 (3H, d, , *J* = 6.5 Hz, H-11), 1.25 (3H, d, *J* = 6.9 Hz, H-12′), 1.19 (3H, d, *J* = 7.3 Hz, H-12). ^{13}C NMR (100 MHz, $CDCl_3$) δ_C 202.9 (C-4′), 200.9 (C-4), 165.5 (C-5′) 164.7 (C-5), 161.3 (C-6, C-6′), 160.0 (C-8, C-8′), 156.5 (C-10a′), 156.2 (C-10), 142.1 (C-9a, C-9a′), 105.8 (C-5a, C-5a′), 101.8 (C-4a, C-4a′), 101.7 (C-9, C-9′), 99.7 (C-7, C-7′), 99.4 (C-10, C-10′), 78.6 (C-2), 75.7 (C-2′), 46.3 (C-3), 44.6 (C-3′), 19.8 (C-11), 16.8 (C-11′), 10.1 (C-12), 9.8 (C-12′). HRESIMS *m/z* 547.1591 [M + H]+ (calcd for $C_{30}H_{27}O_{10}^+$ 547.1599).

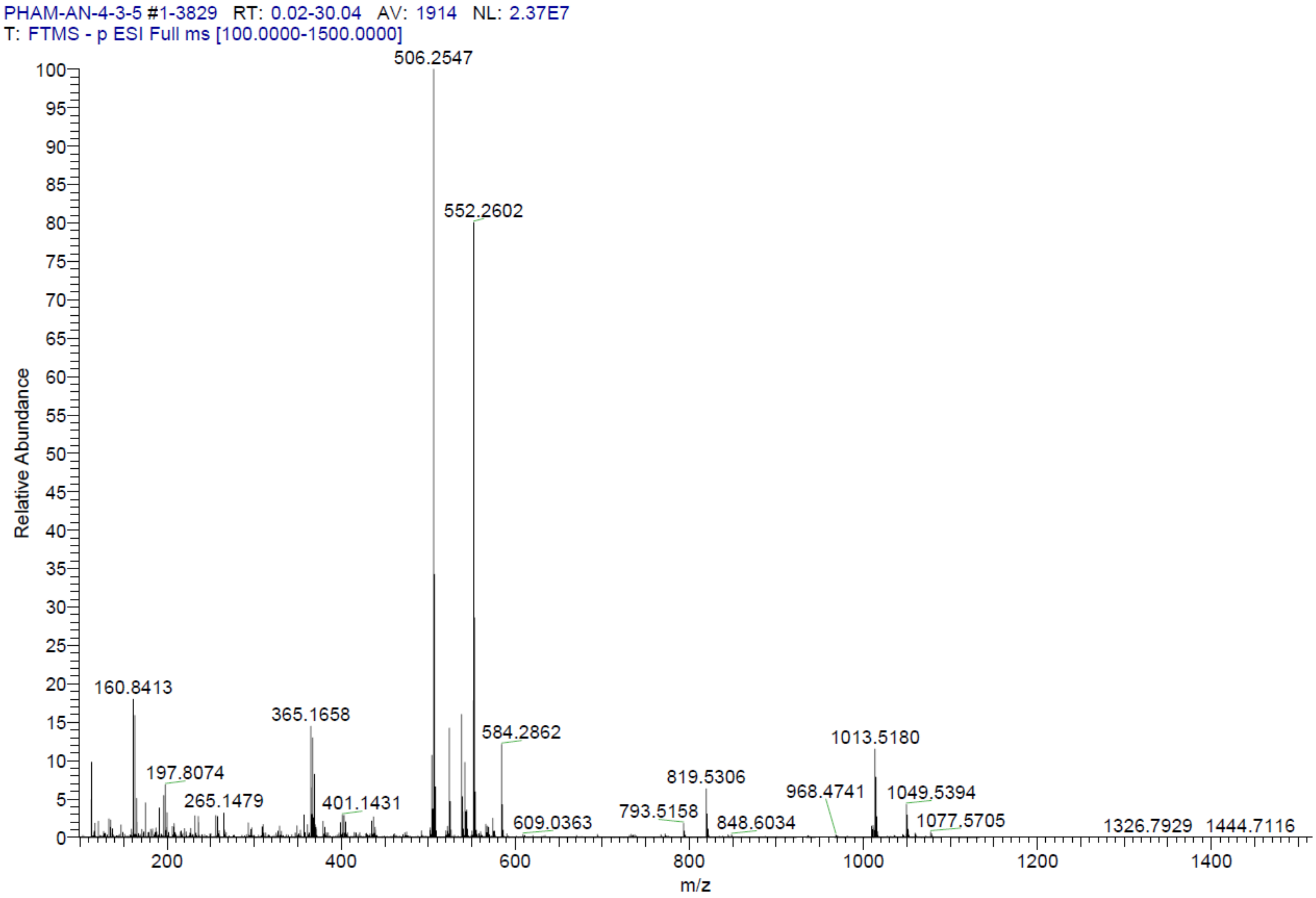

Figure S9. HRESIMS of **2**

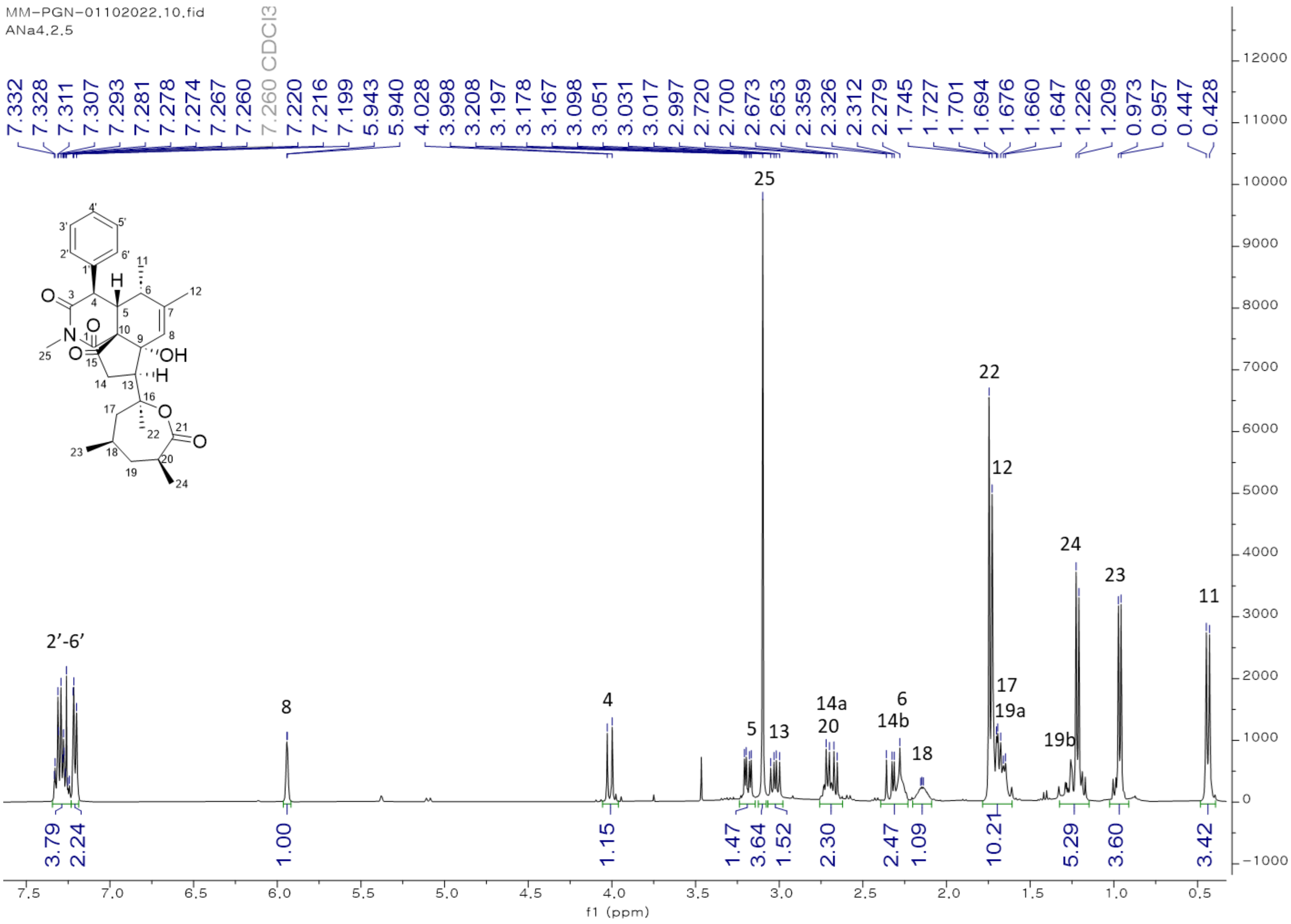

Figure S10. ^{1}H NMR (400 MHz) spectrum of **2** in $CDCl_3$

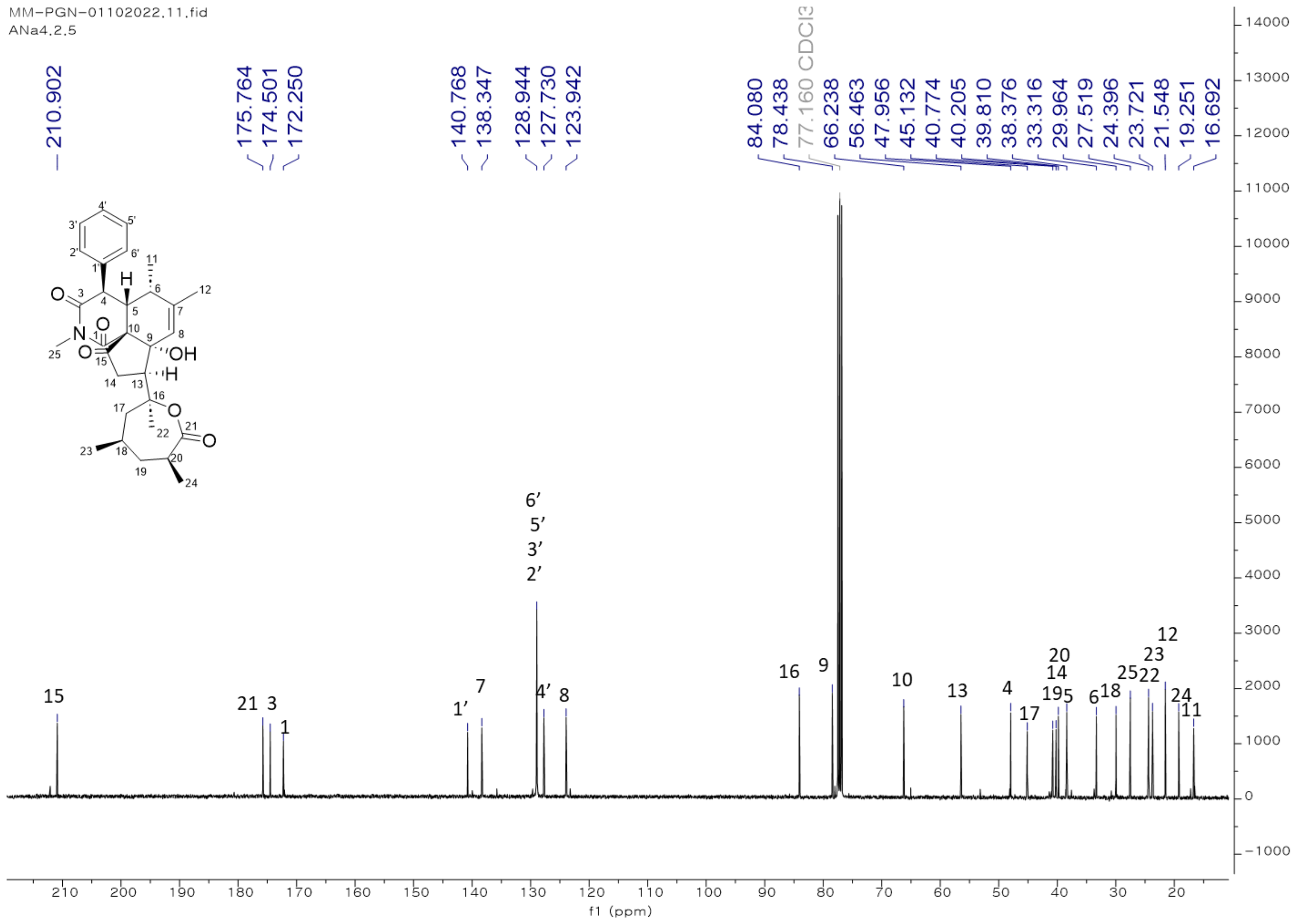

Figure S11. ^{13}C NMR (100 MHz) spectrum of **2** in $CDCl_3$

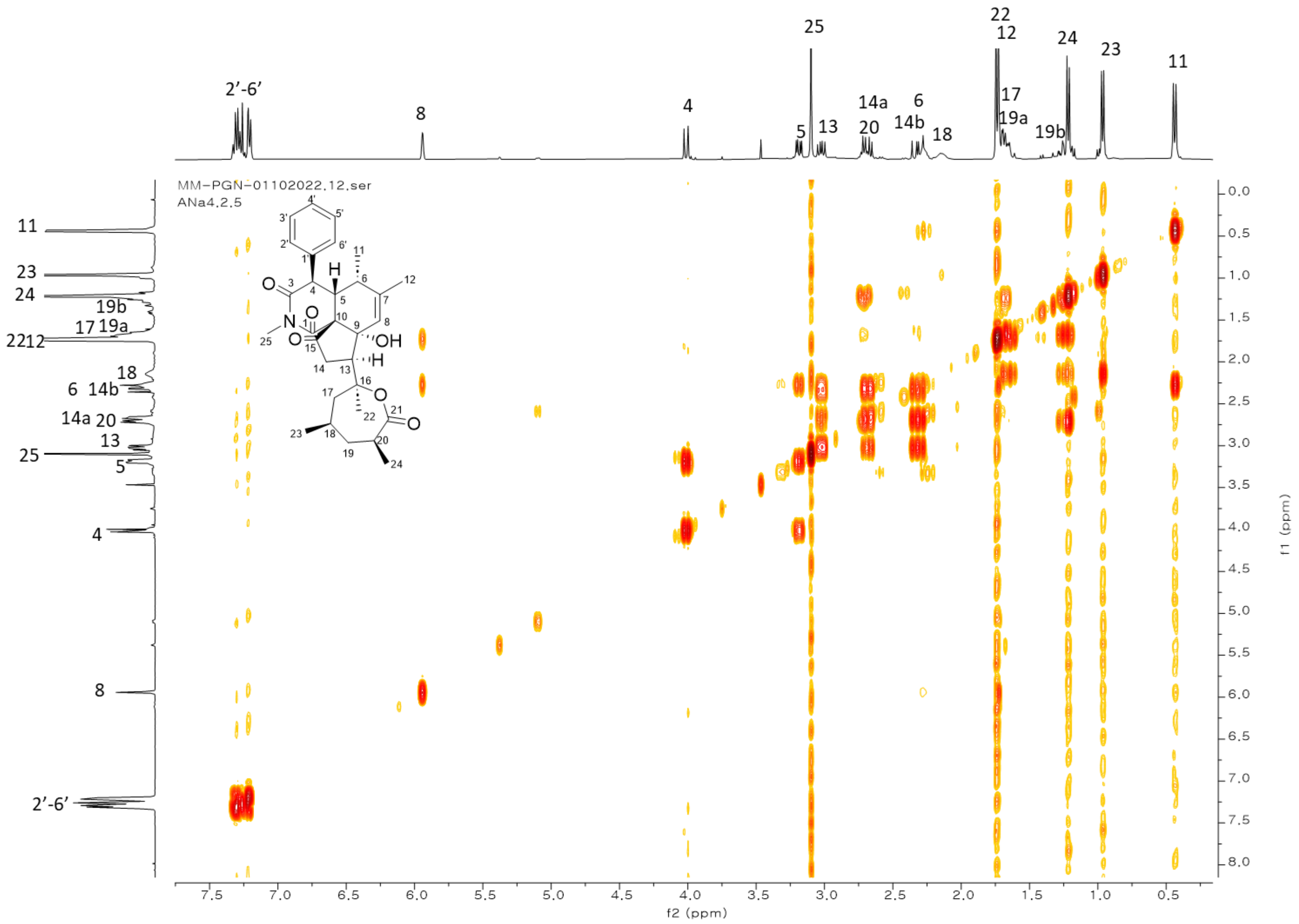

Figure S12. ^1H-^1H COSY spectrum of **2** in $CDCl_3$

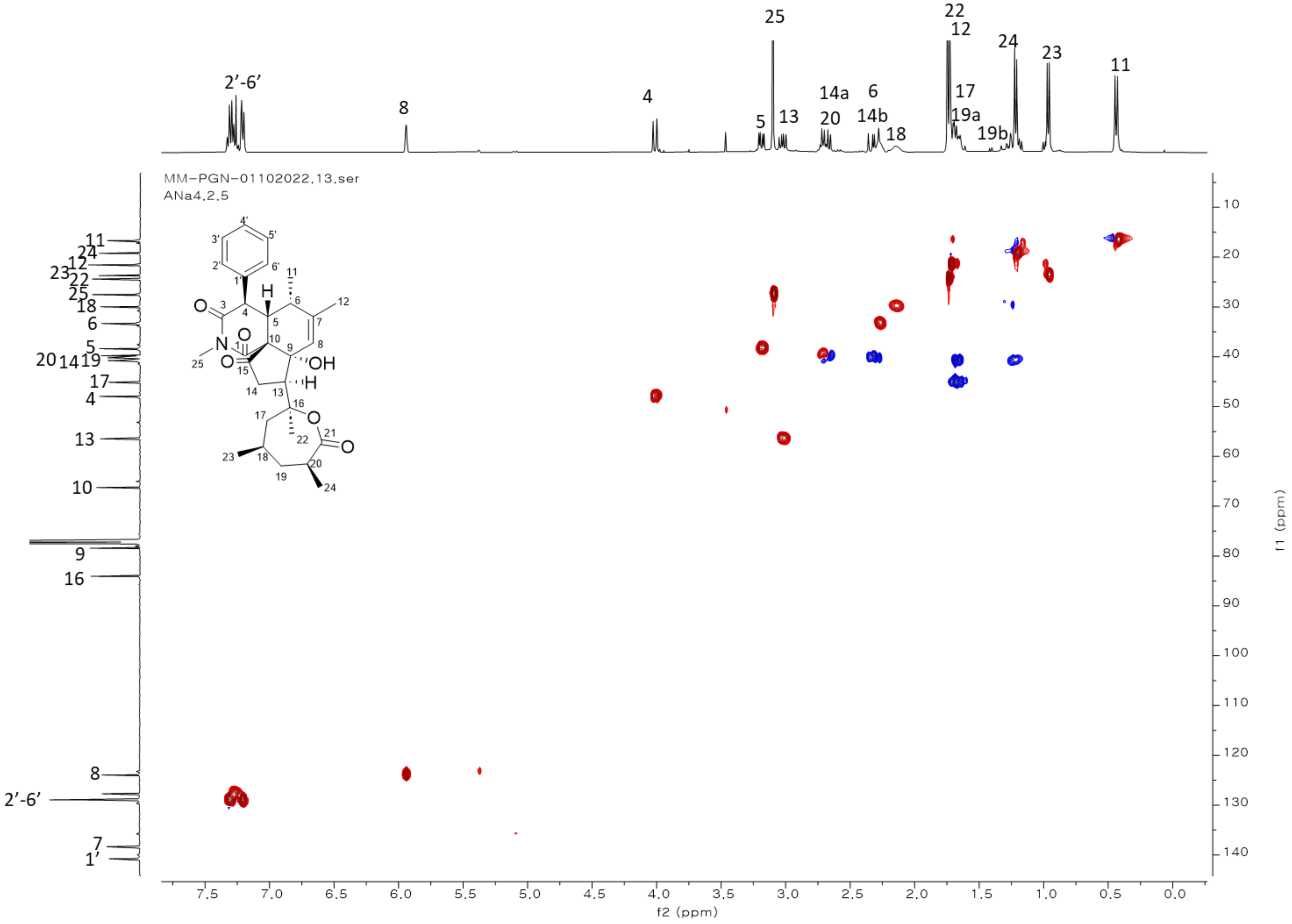

Figure S13. ^{1}H-^{13}C HSQC spectrum of **2** in $CDCl_3$

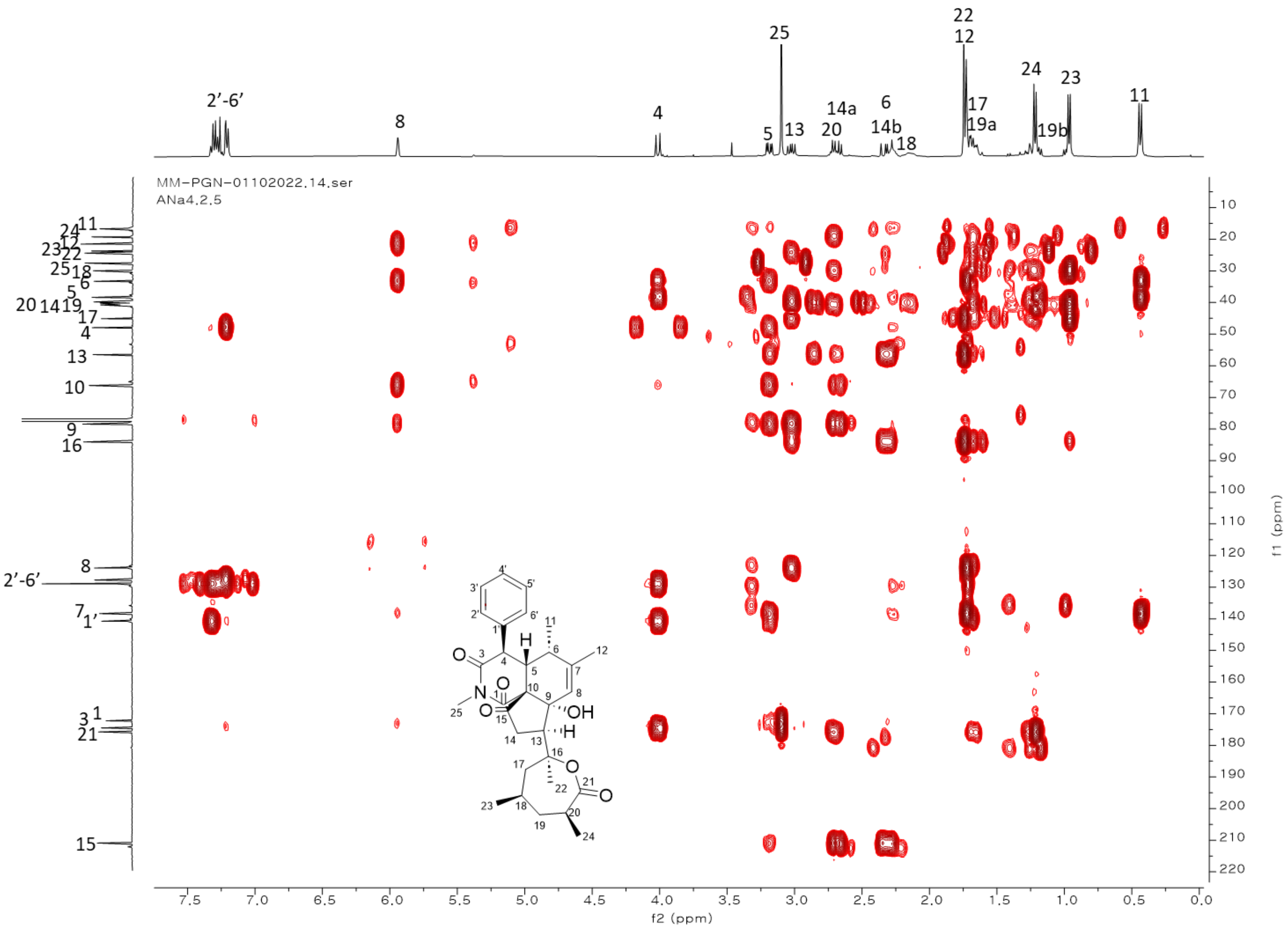

Figure S14. ^1H-^{13}C HMBC spectrum of **2** in $CDCl_3$

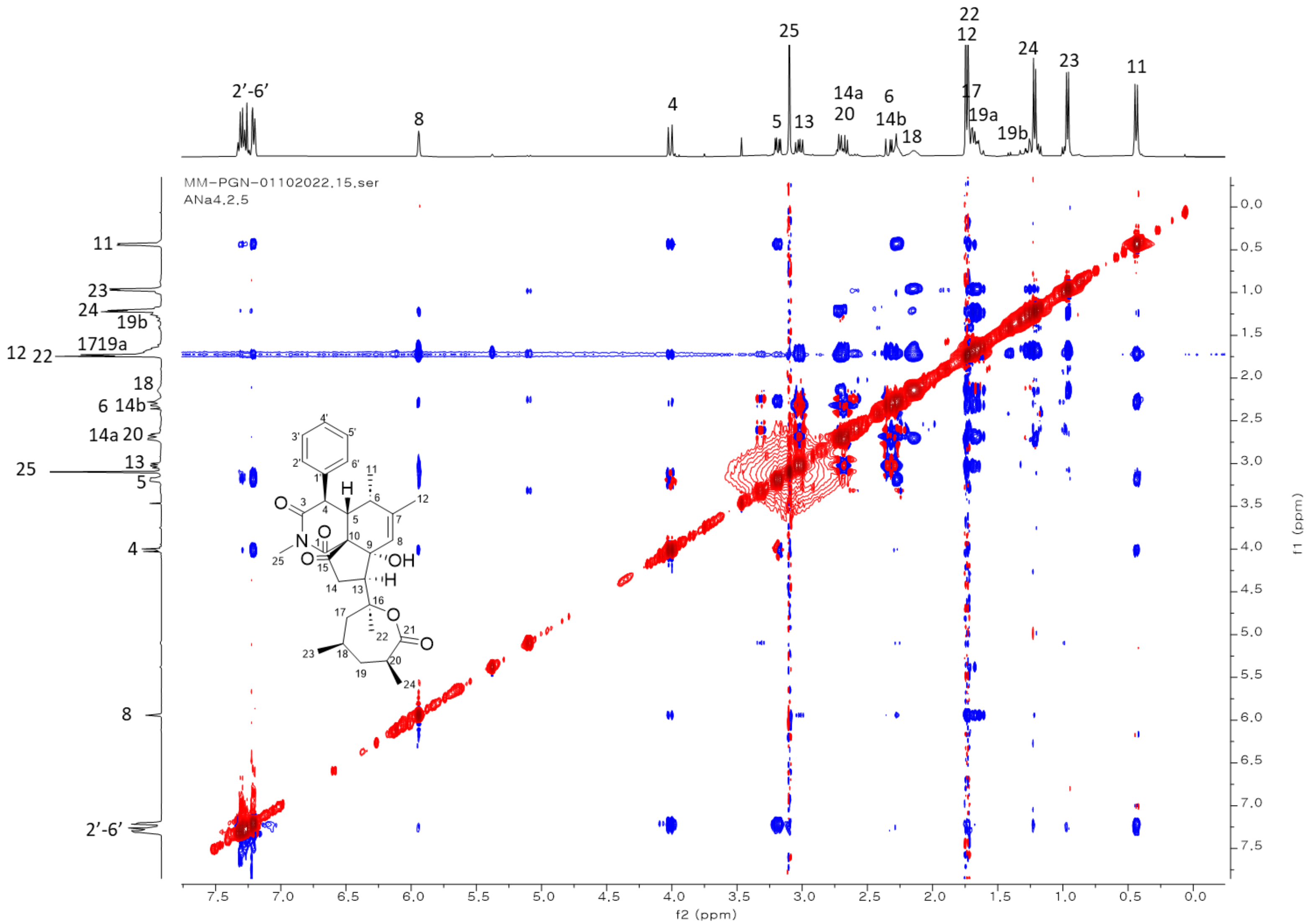

Figure S15. ^{1}H-^{1}H NOESY spectrum of **2** in $CDCl_3$

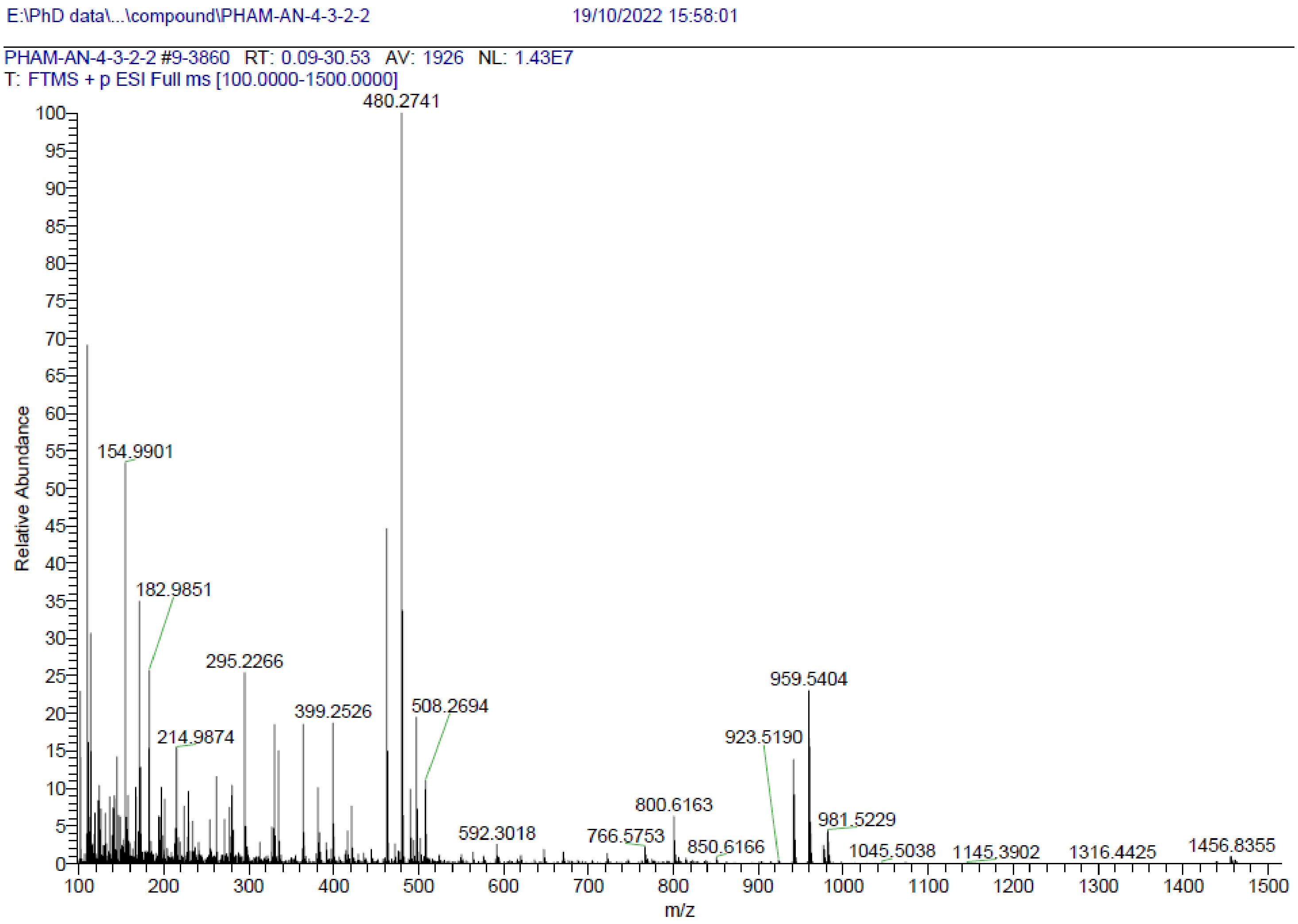

Figure S16. HRESIMS of **3**

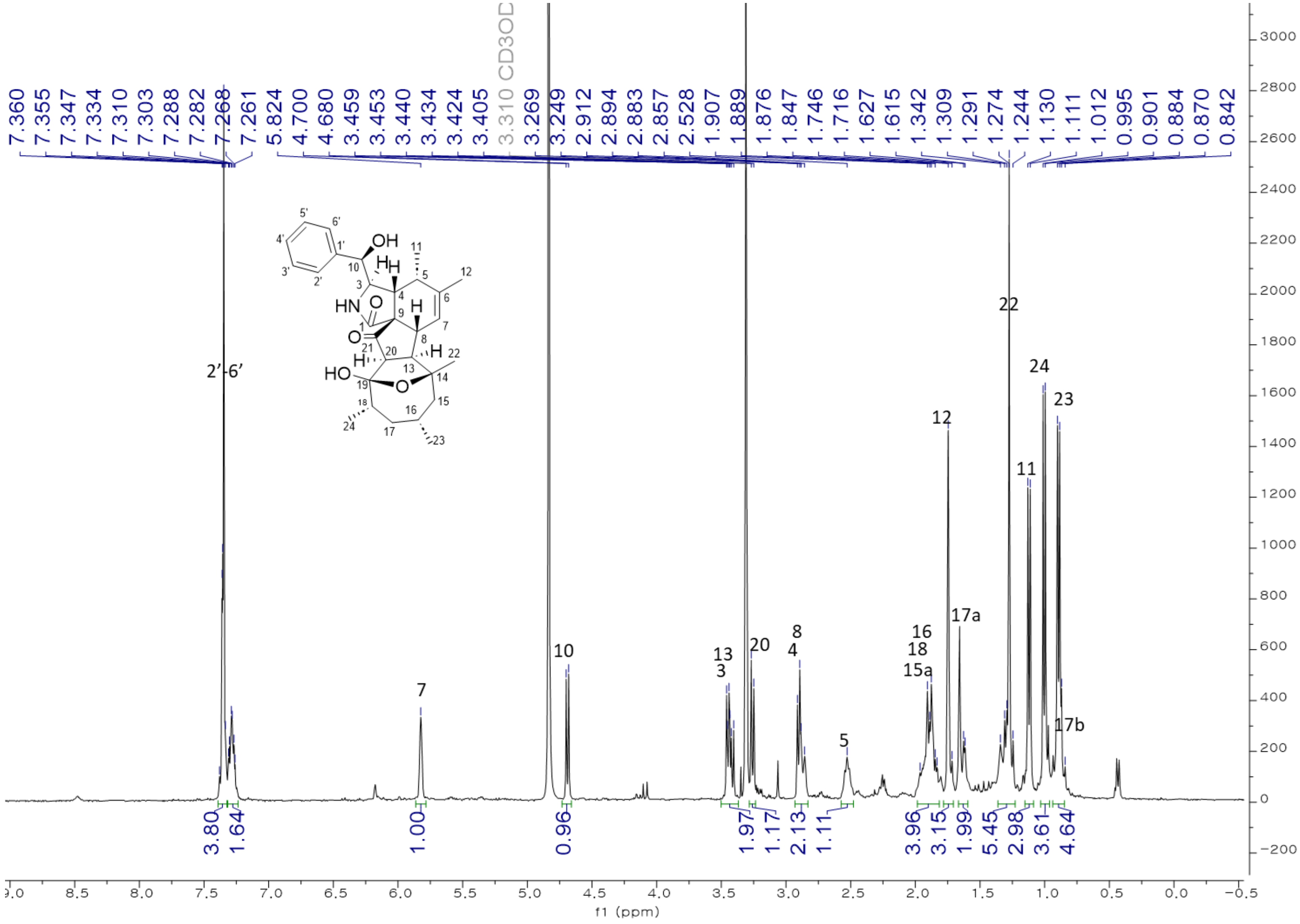

Figure S17. ^{1}H NMR (400 MHz) spectrum of **3** in CD_3OD

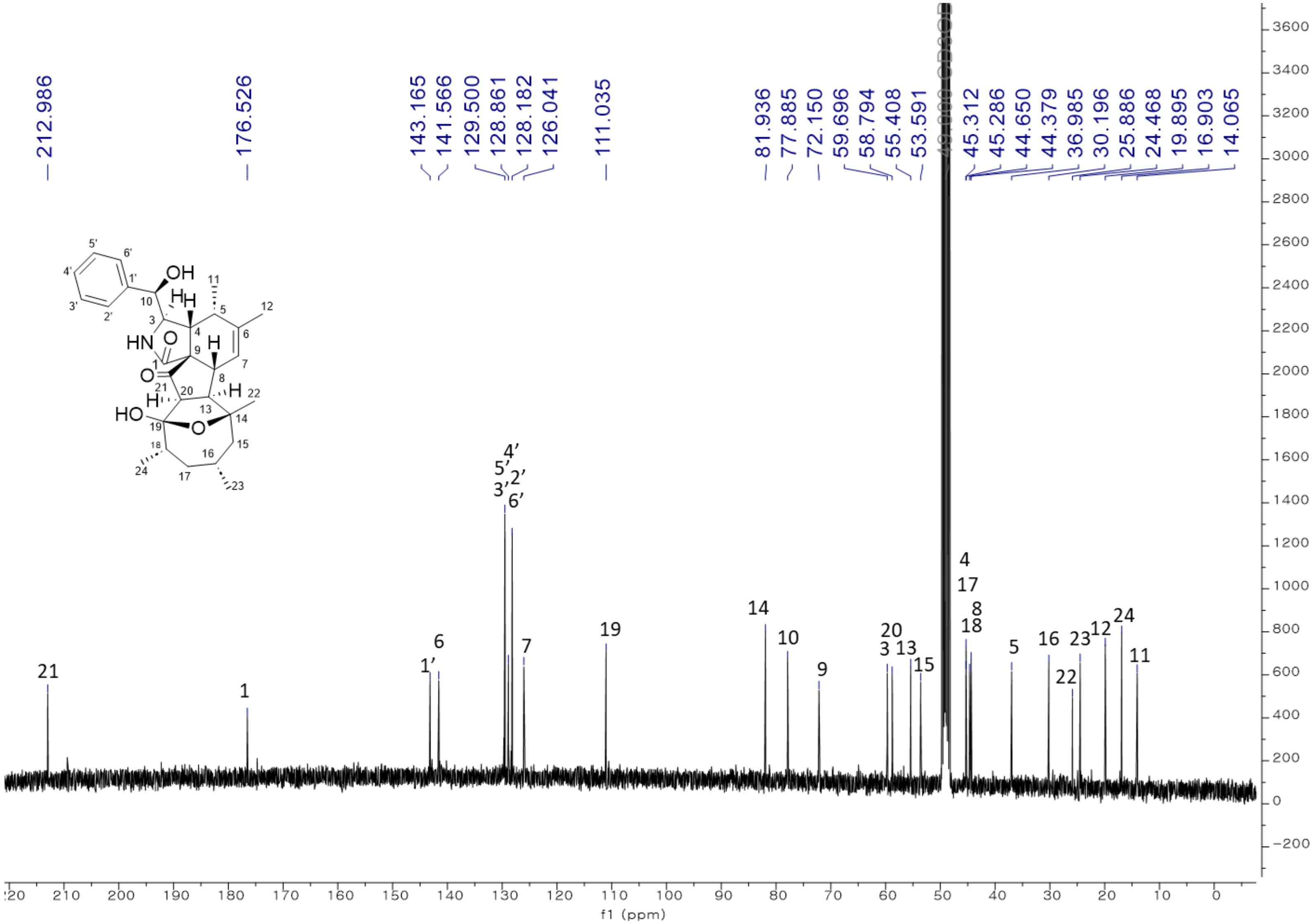

Figure S18. ^{13}C NMR (100 MHz) spectrum of **3** in CD_3OD

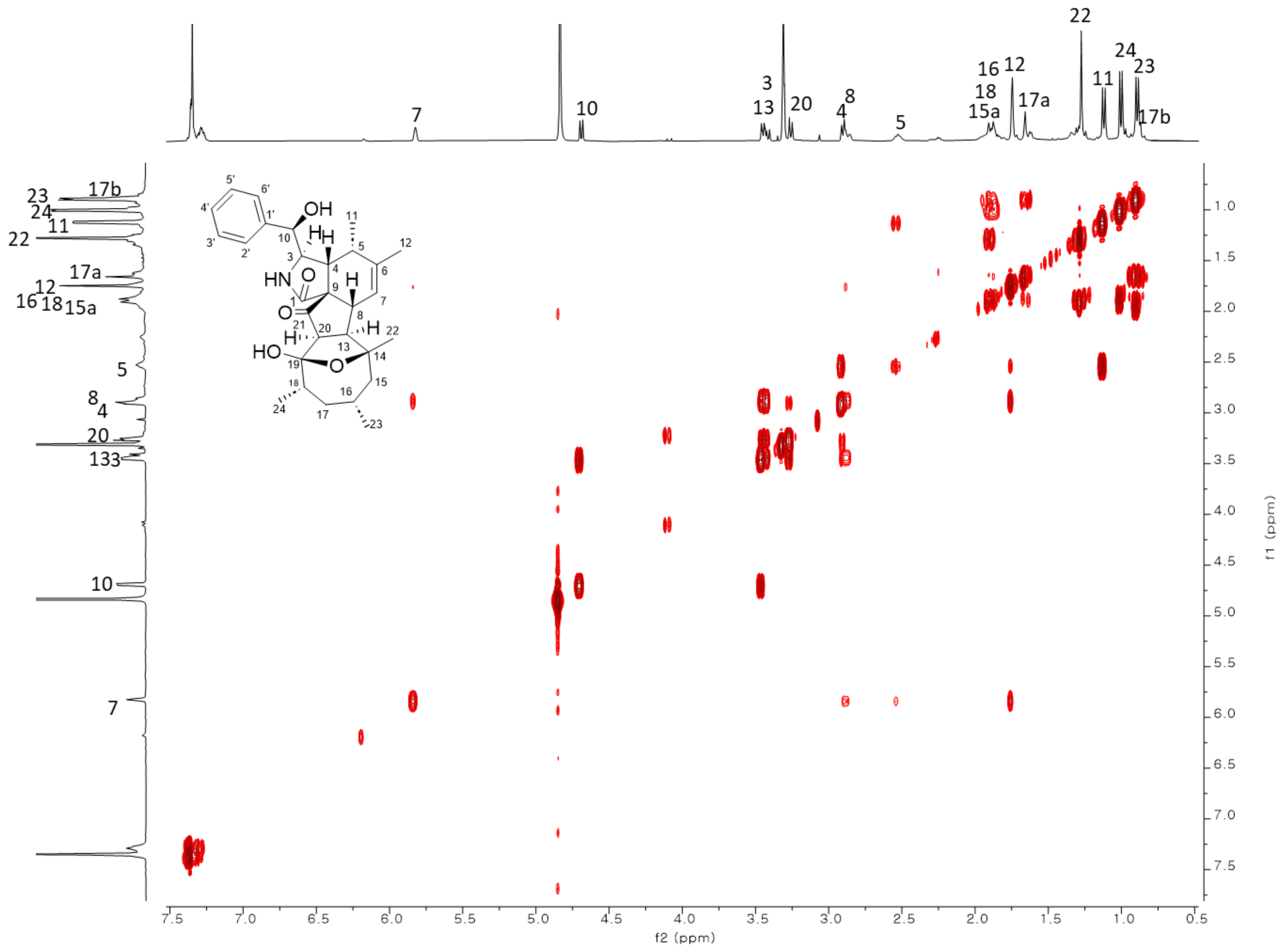

Figure S19. ^{1}H-^{1}H COSY spectrum of **3** in CD_3OD

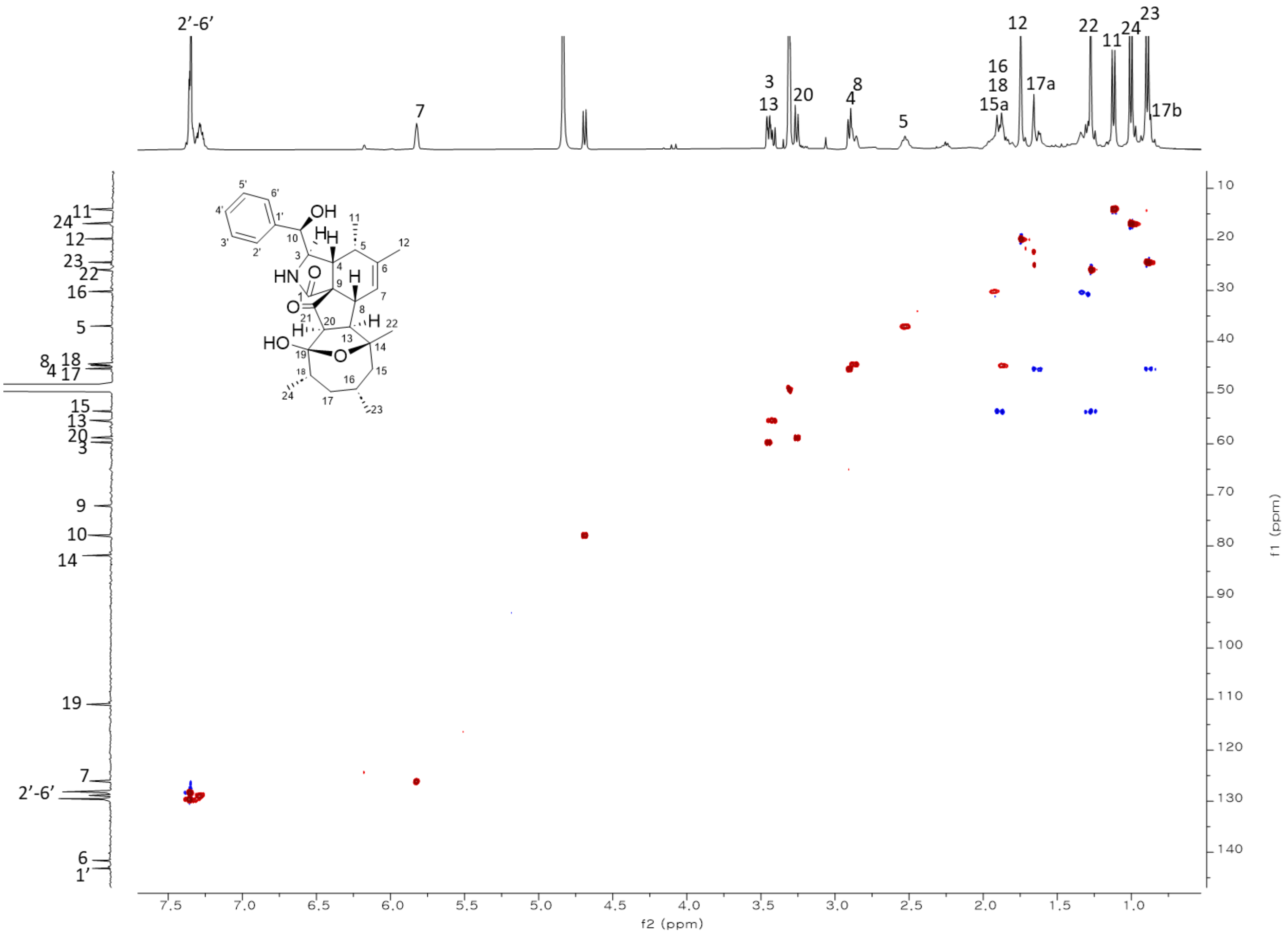

Figure S20. ^{1}H-^{13}C HSQC spectrum of **3** in CD_3OD

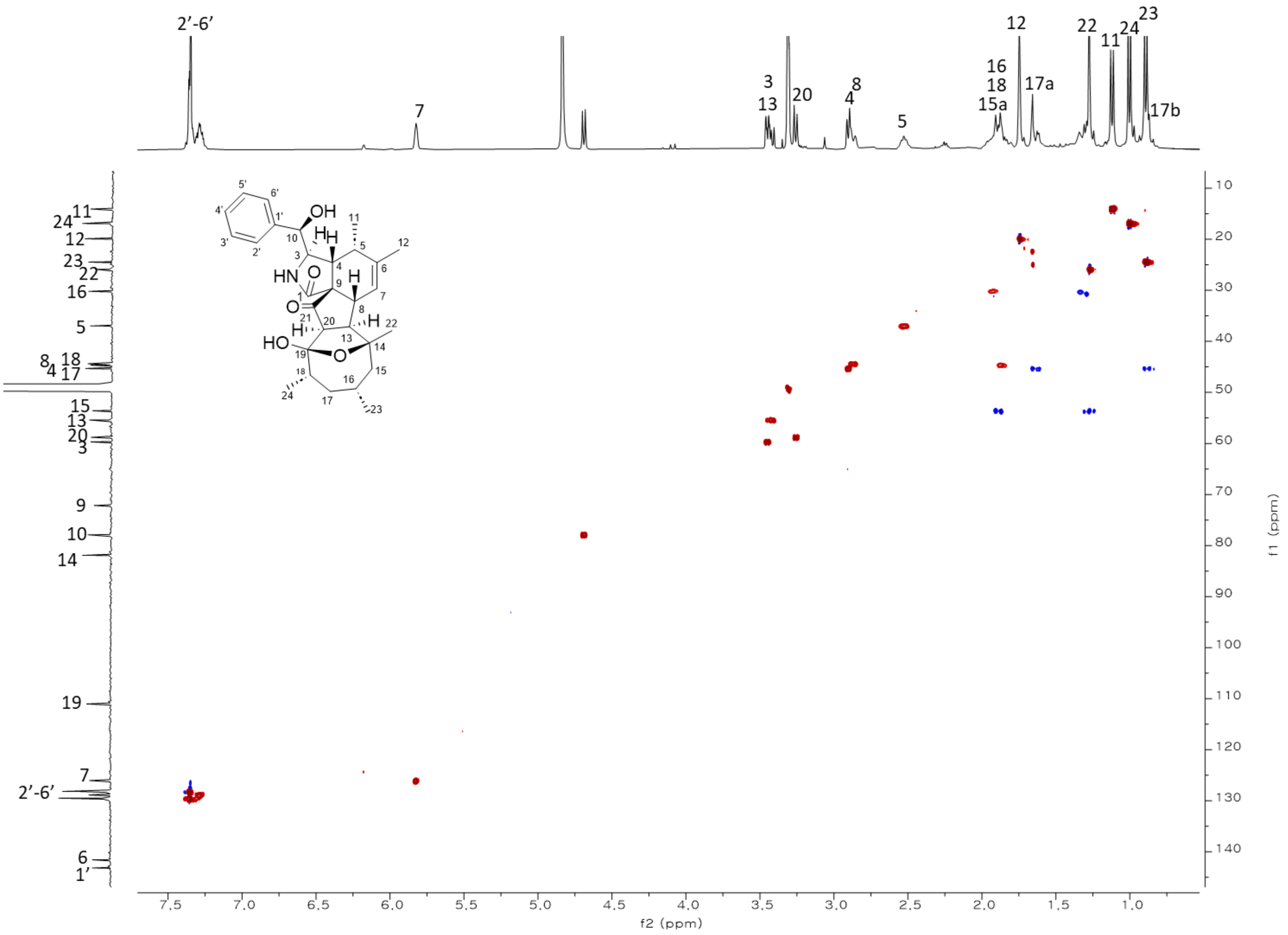

Figure S21. ^{1}H-^{13}C HMBC spectrum of **3** in CD_3OD

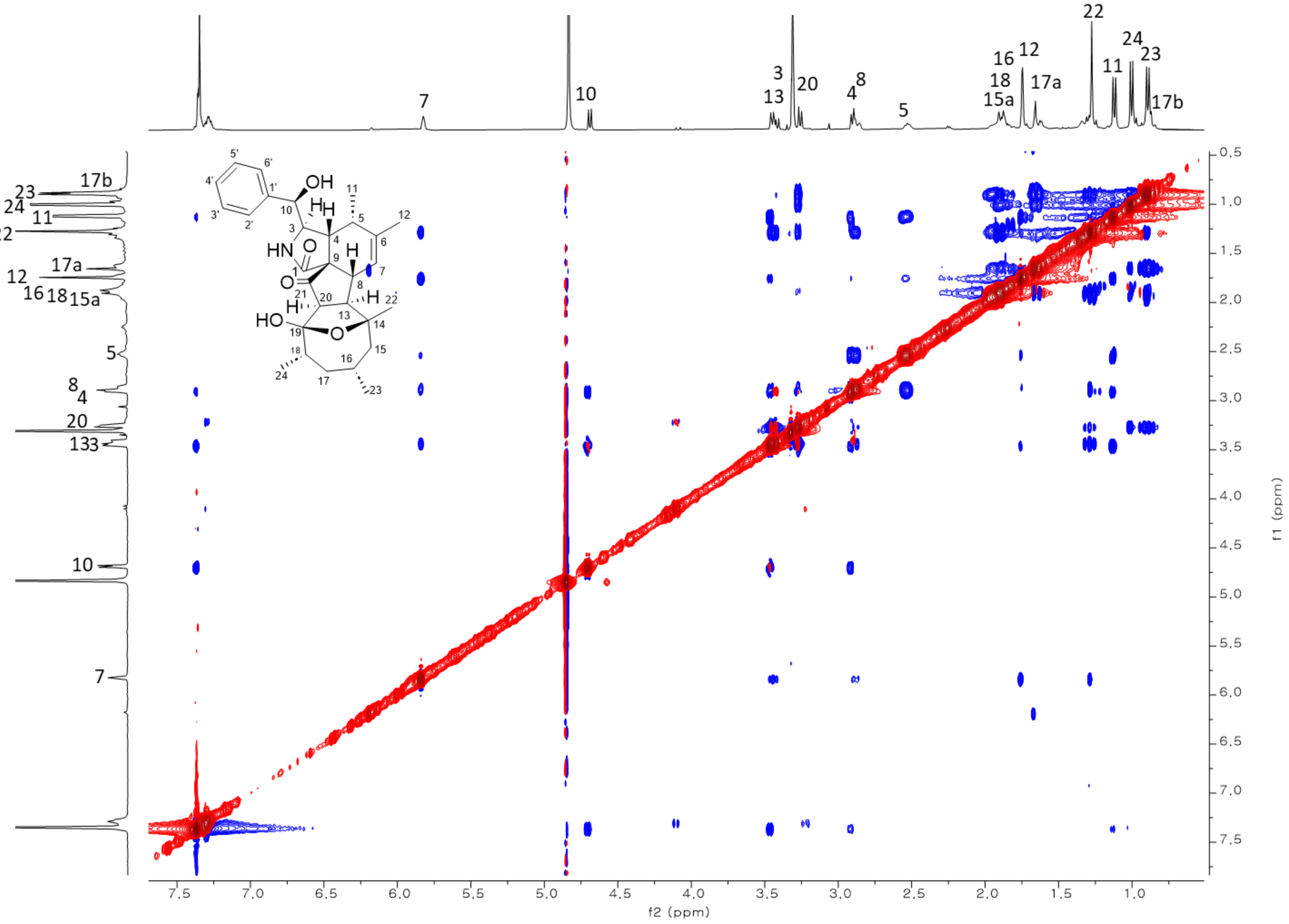

Figure S22. ^{1}H-^{1}H NOESY spectrum of **3** in CD_3OD

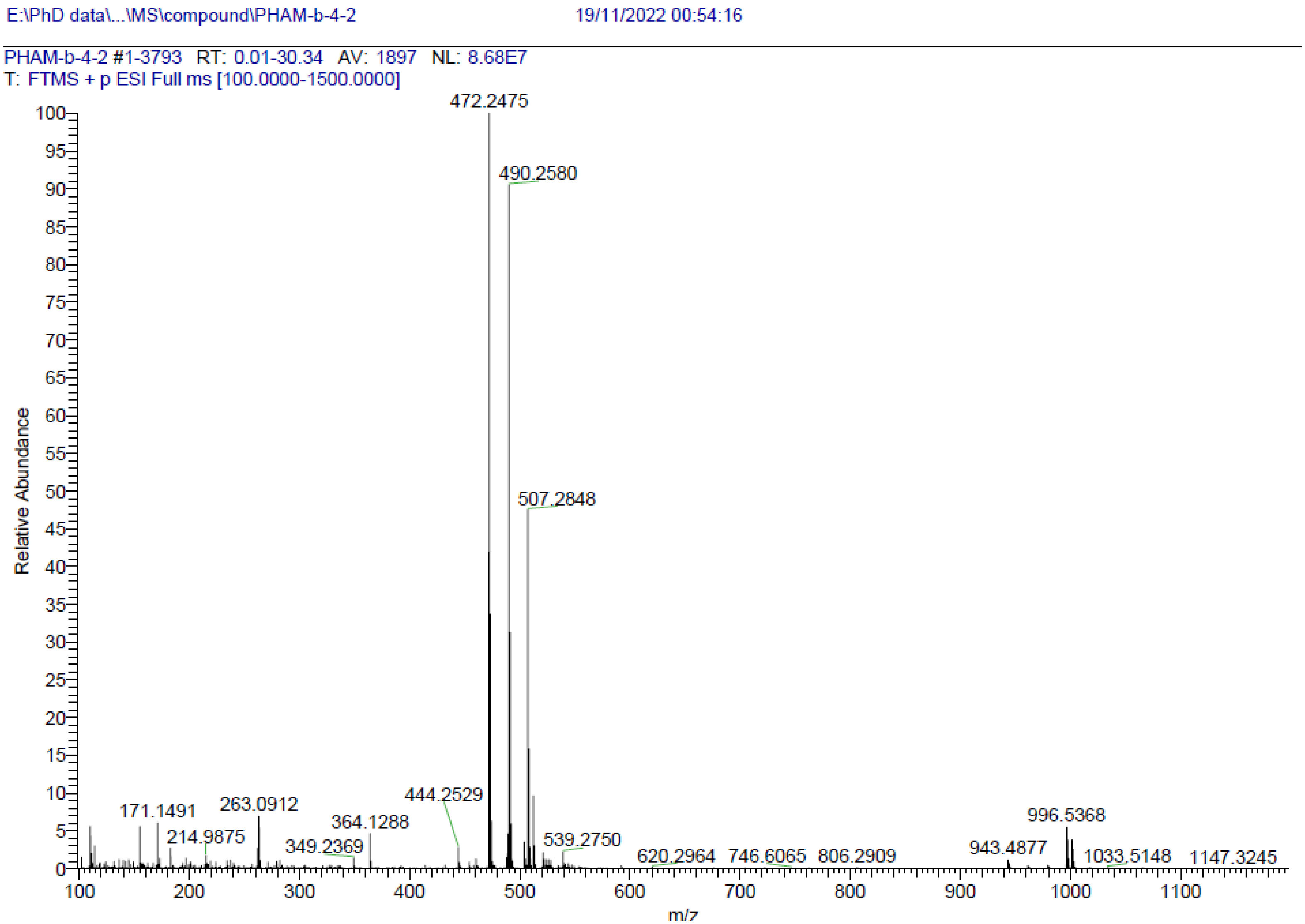

Figure S23. HRESIMS of **4**

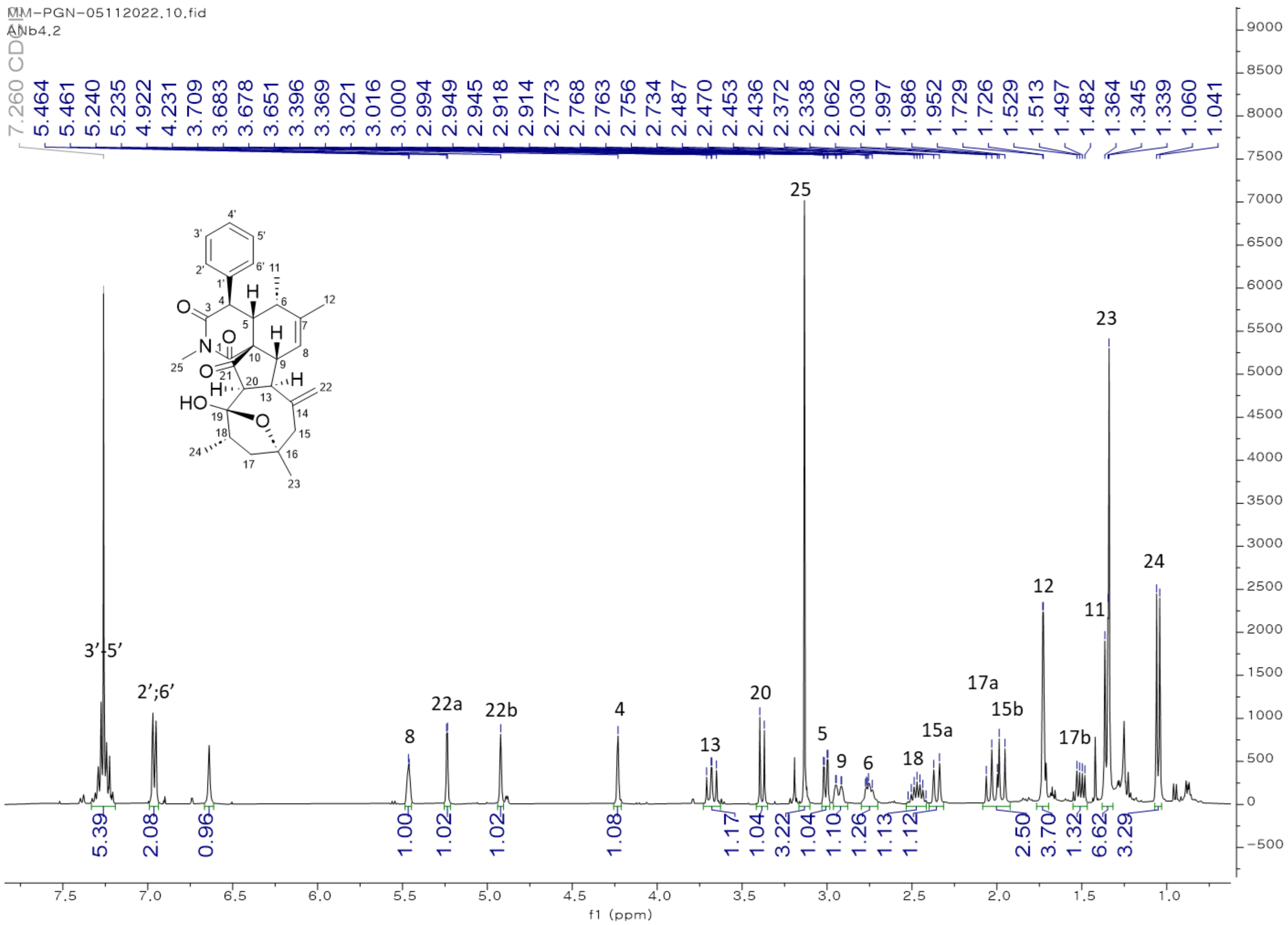

Figure S24. ^{1}H NMR (400 MHz) spectrum of **4** in $CDCl_3$

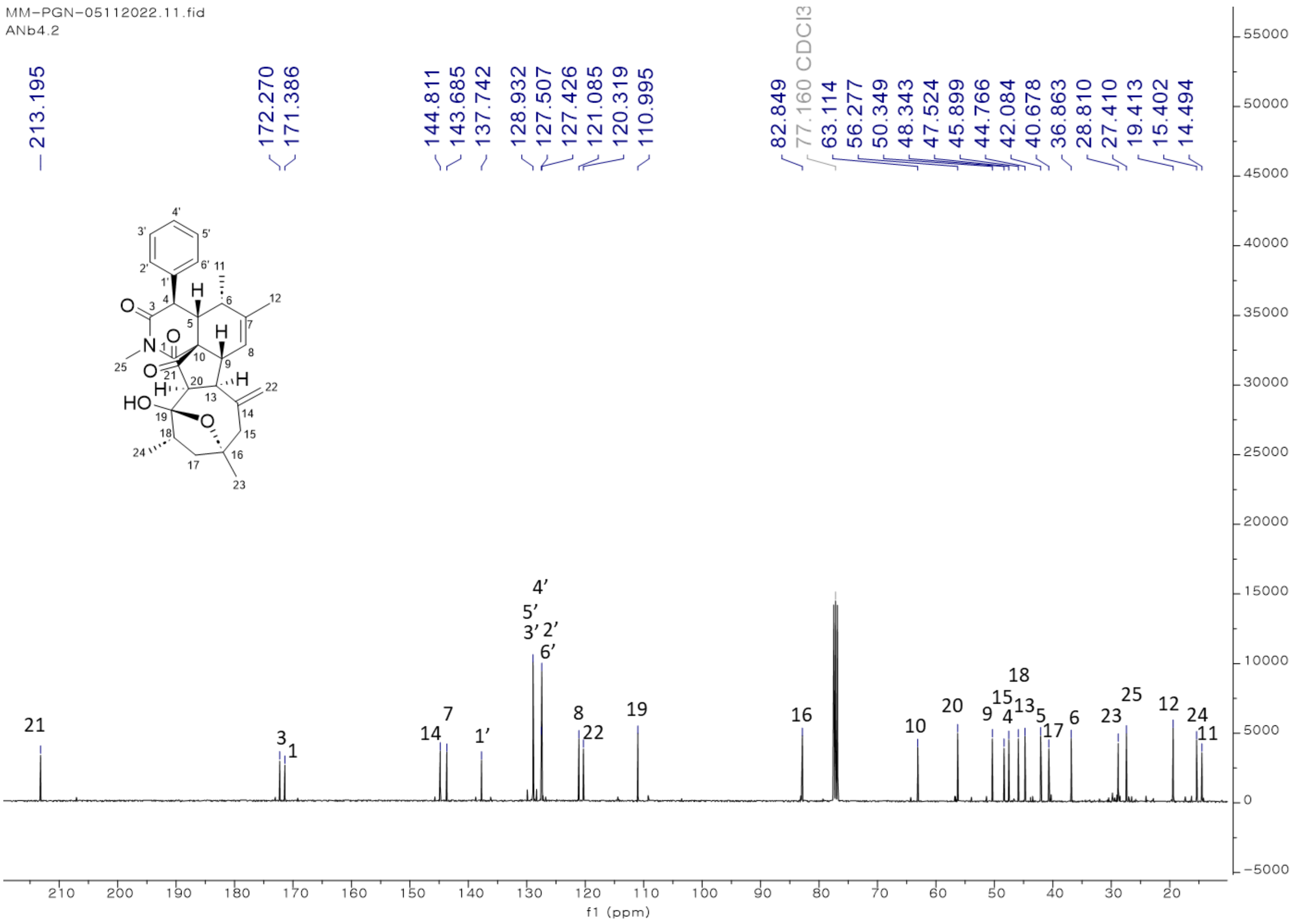

Figure S25. ^{13}C NMR (100 MHz) spectrum of **4** in $CDCl_3$

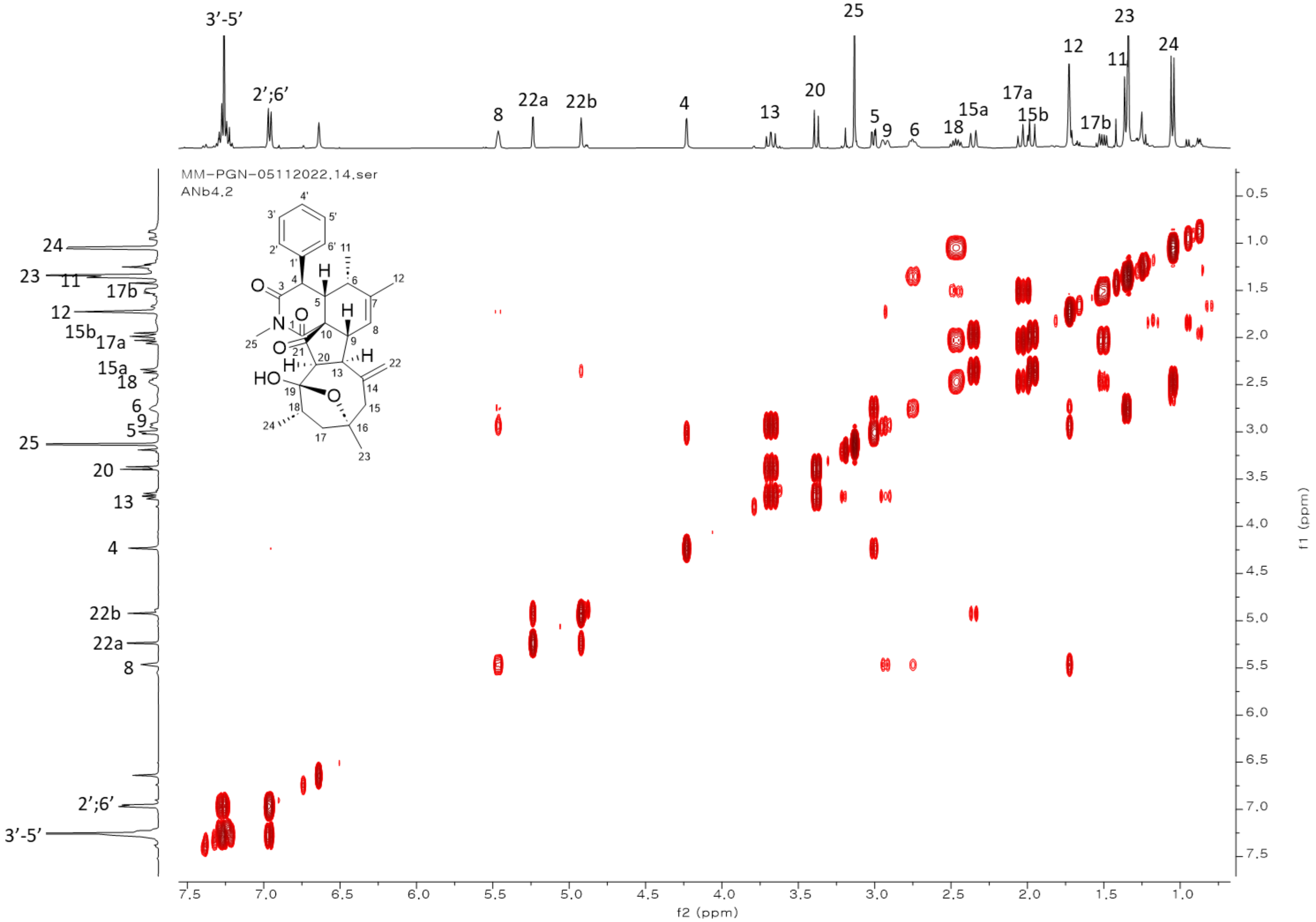

Figure S26. ^{1}H-^{1}H COSY spectrum of **4** in $CDCl_3$

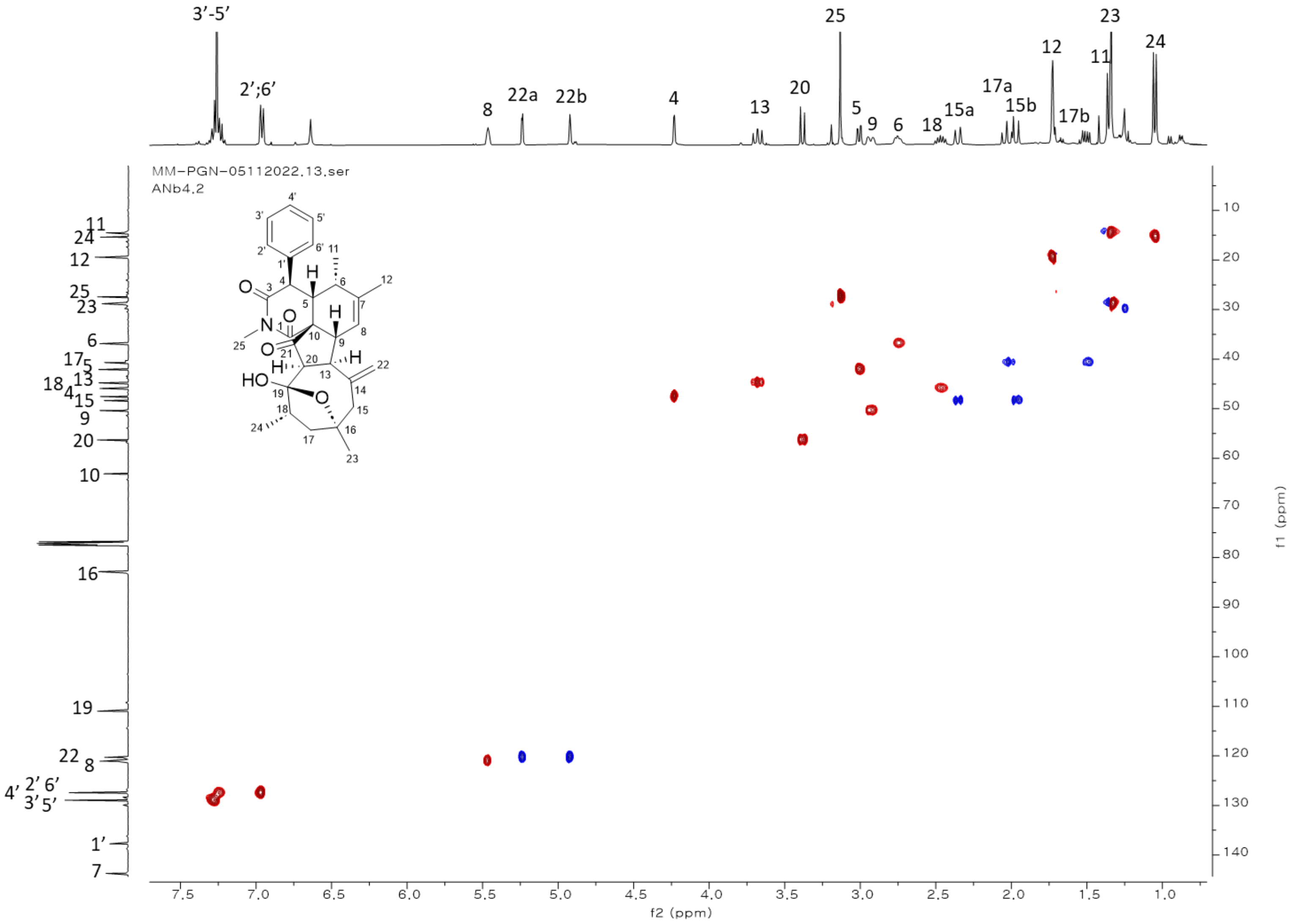

Figure S27. ^{1}H-^{13}C HSQC spectrum of **4** in $CDCl_3$

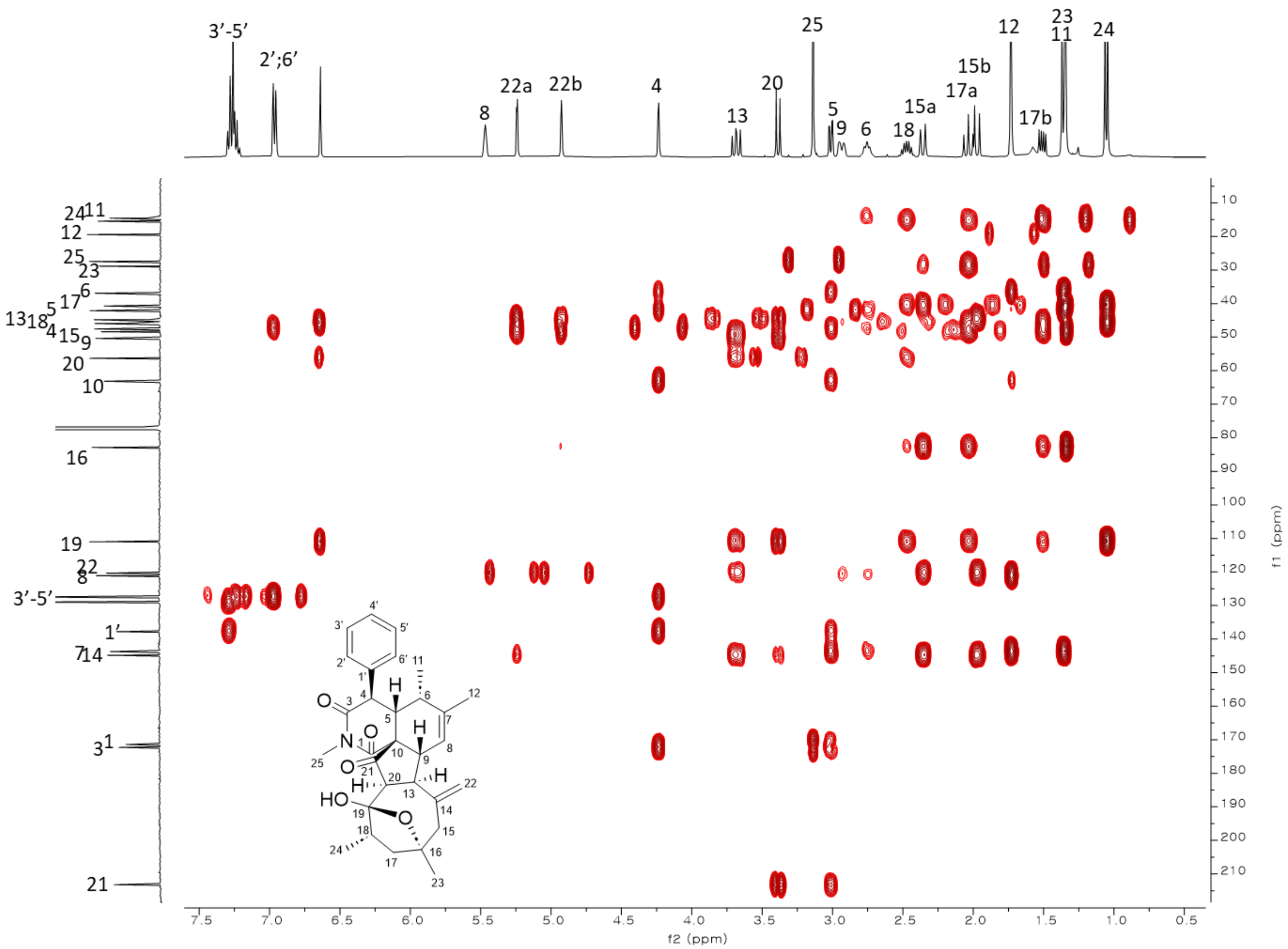

Figure S28. ^{1}H-^{13}C HMBC spectrum of **4** in $CDCl_3$

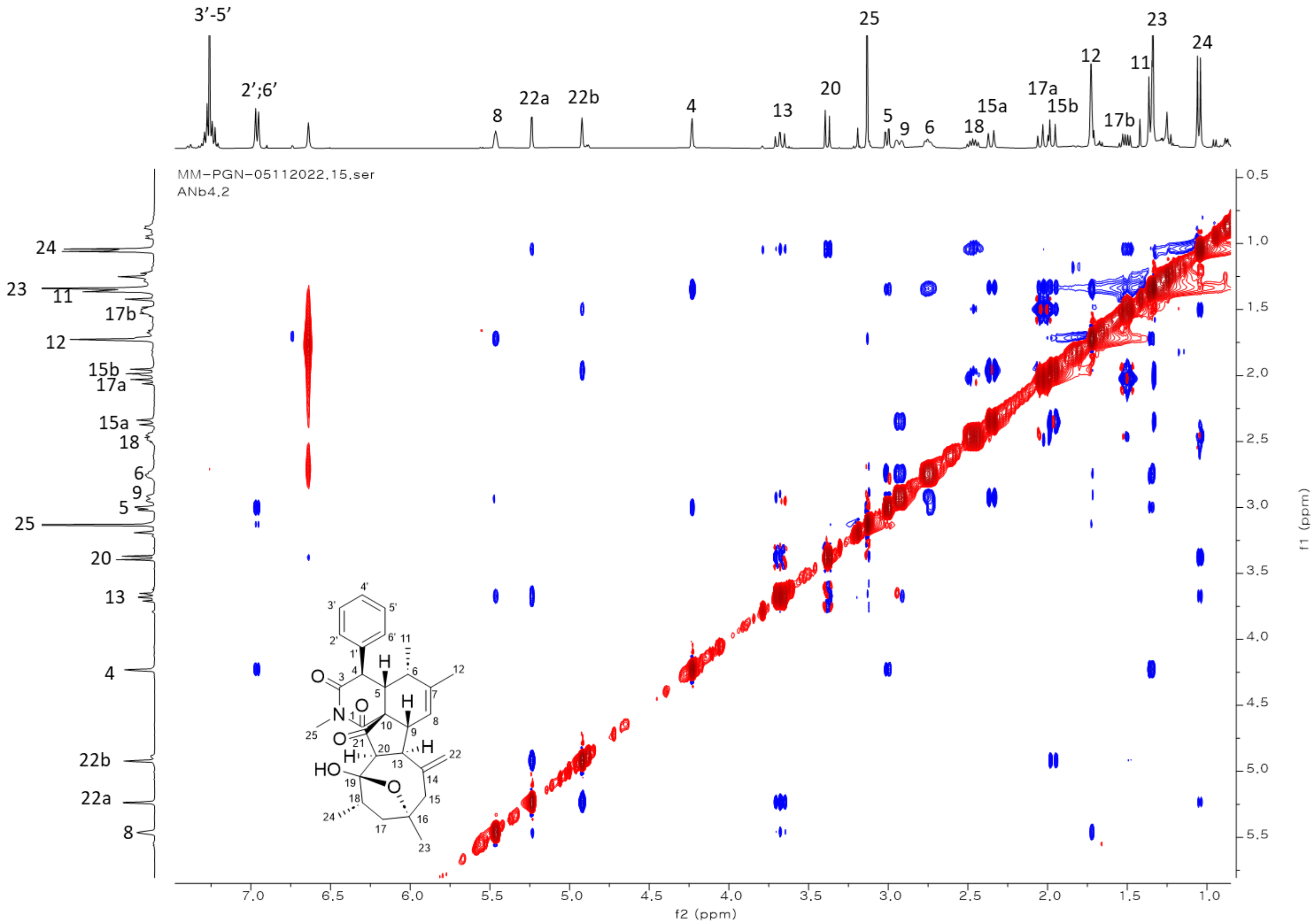

Figure S29. ^{1}H-^{1}H NOESY spectrum of **4** in $CDCl_3$

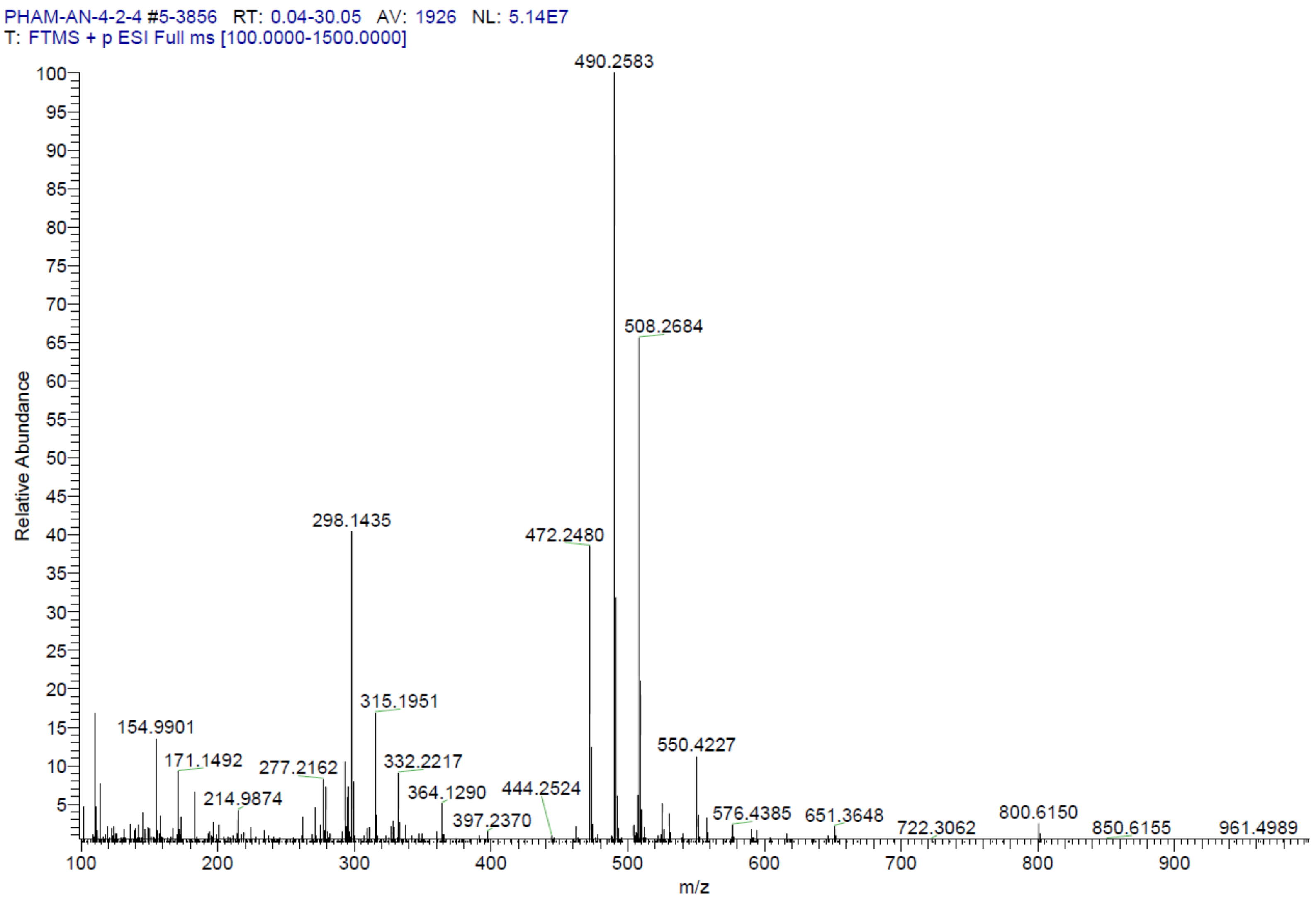

Figure S30. HRESIMS of **5**

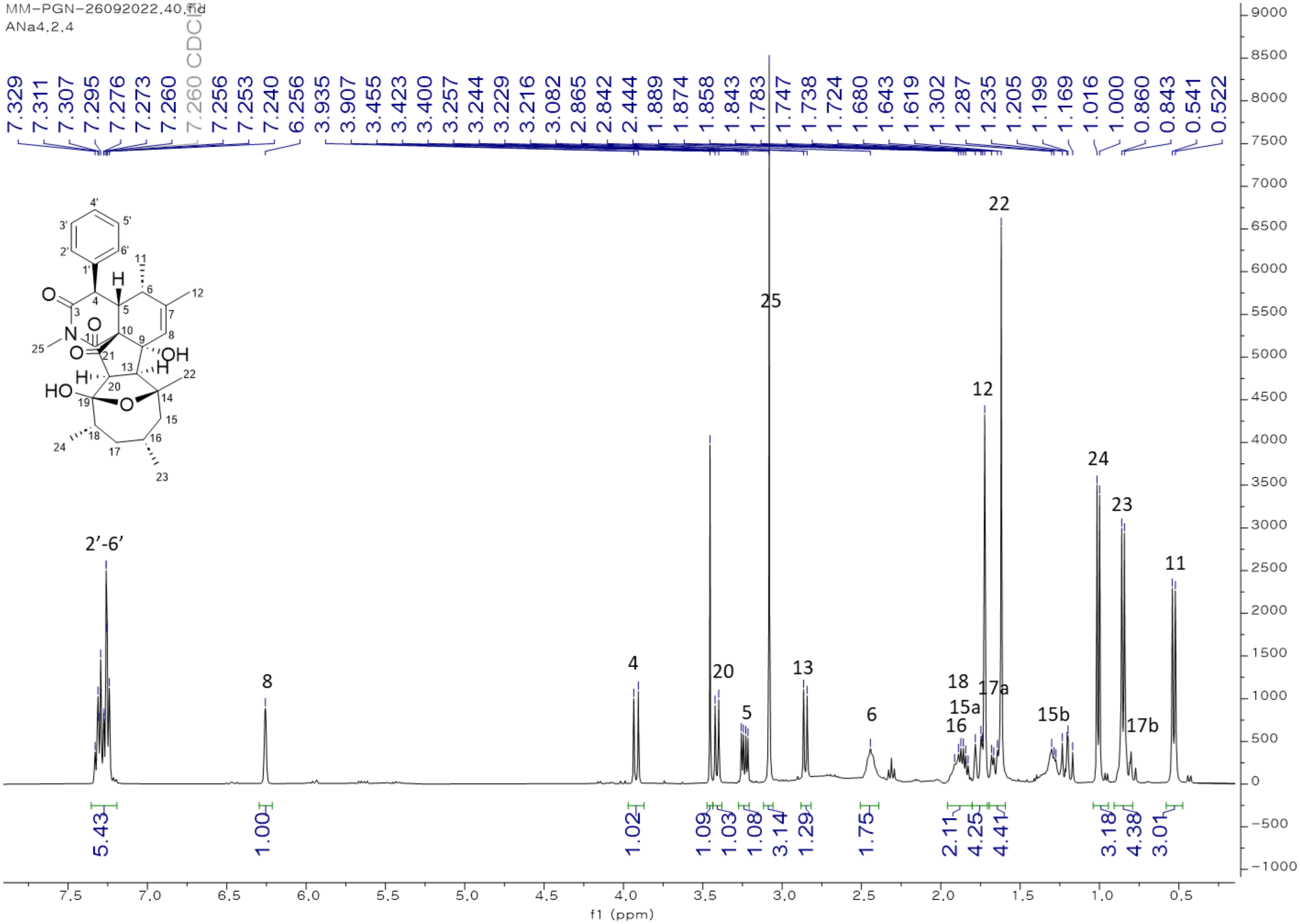

Figure S31. ^{1}H NMR (400 MHz) spectrum of **5** in $CDCl_3$

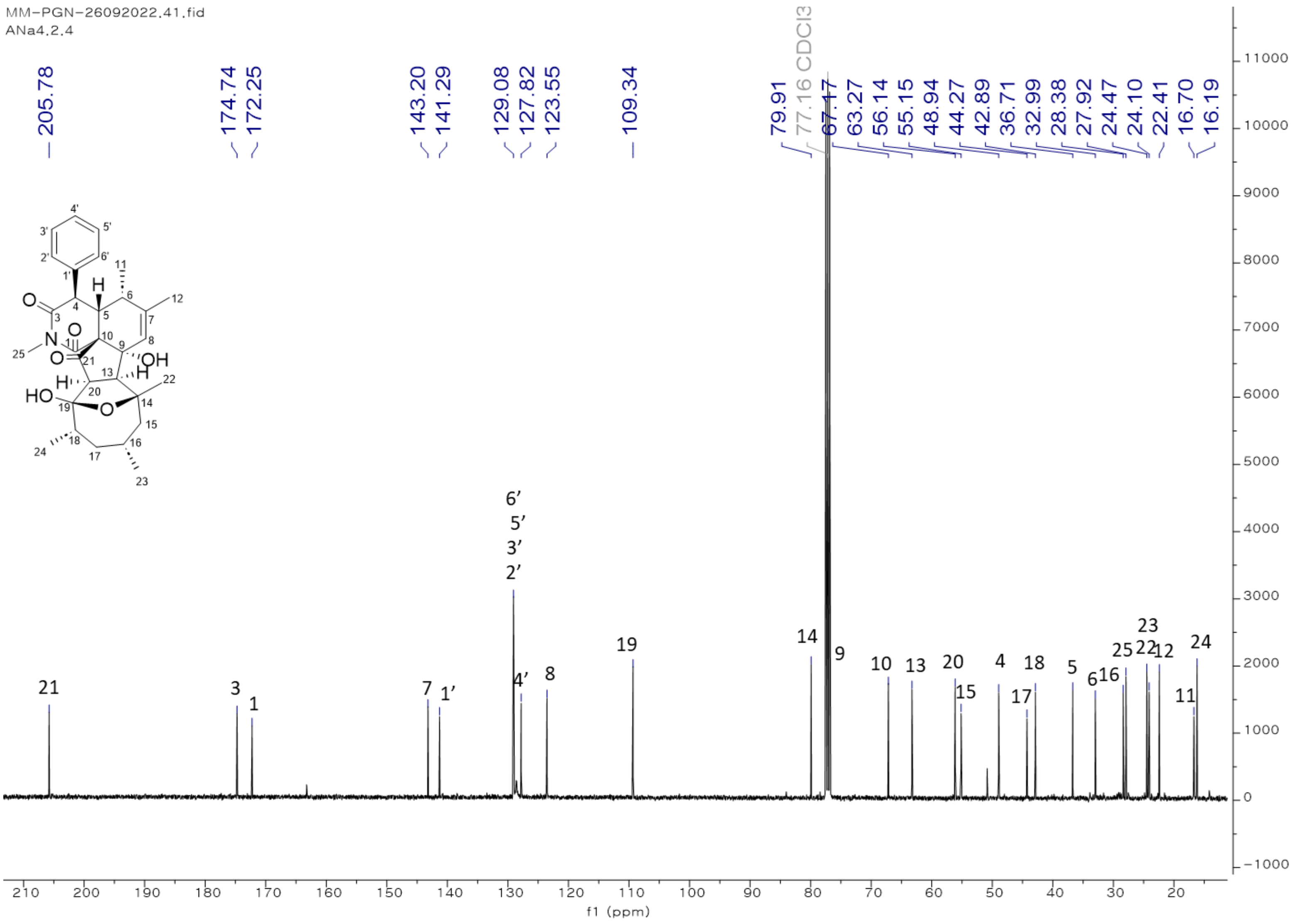

Figure S32. ^{13}C NMR (100 MHz) spectrum of **5** in $CDCl_3$

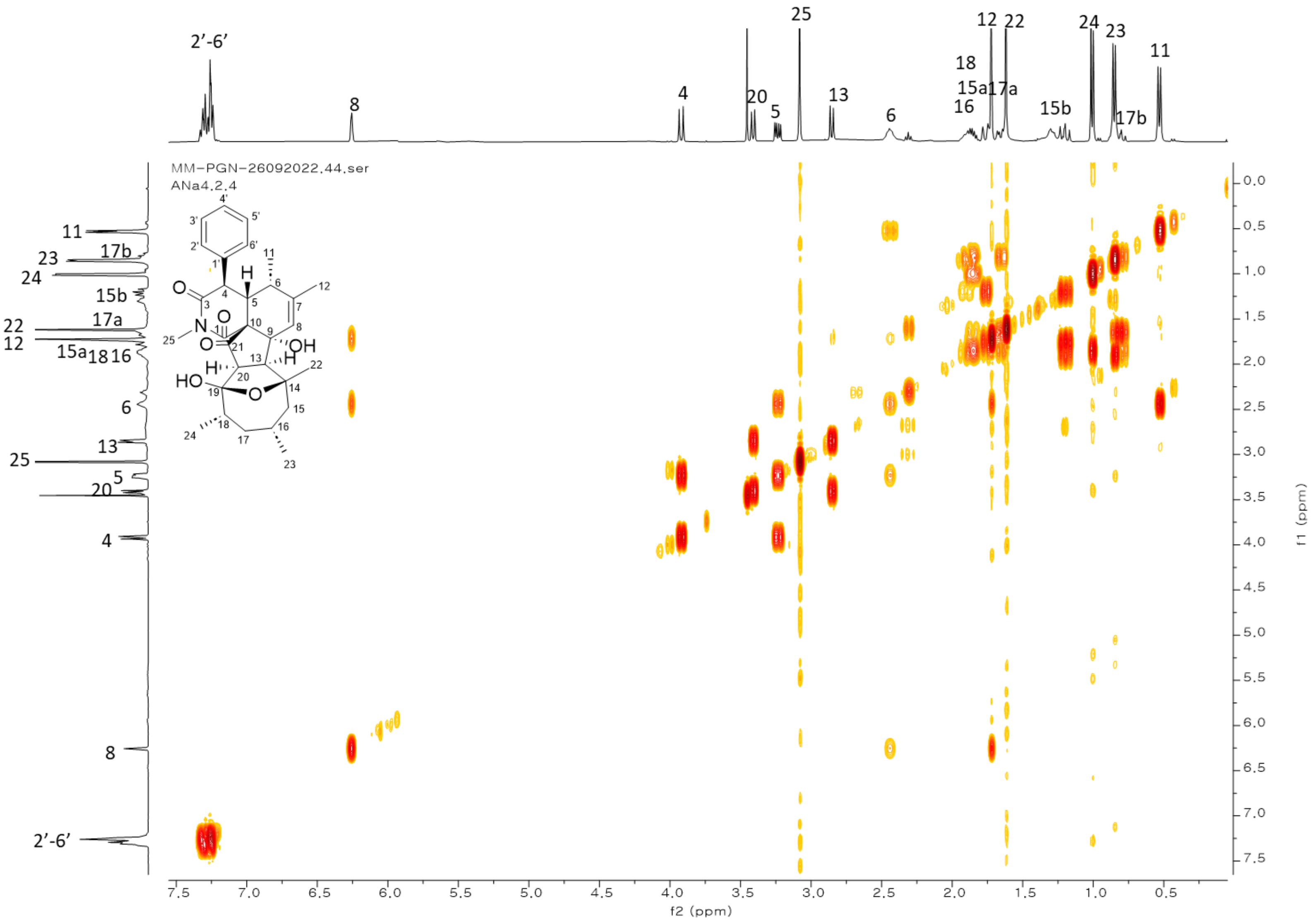

Figure S33. ^{1}H-^{1}H COSY spectrum of **5** in $CDCl_3$

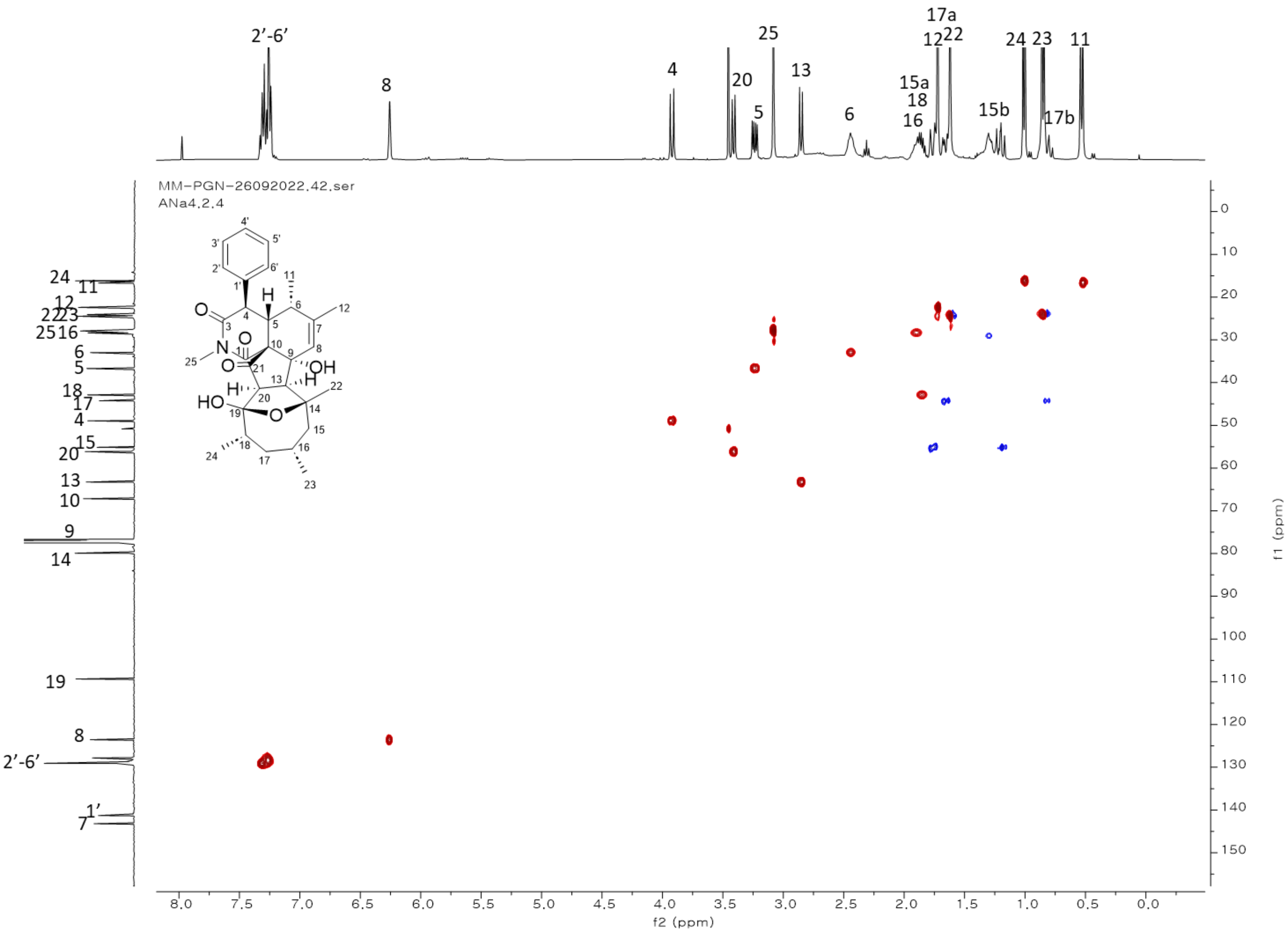

Figure S34. ^{1}H-^{13}C HSQC spectrum of **5** in $CDCl_3$

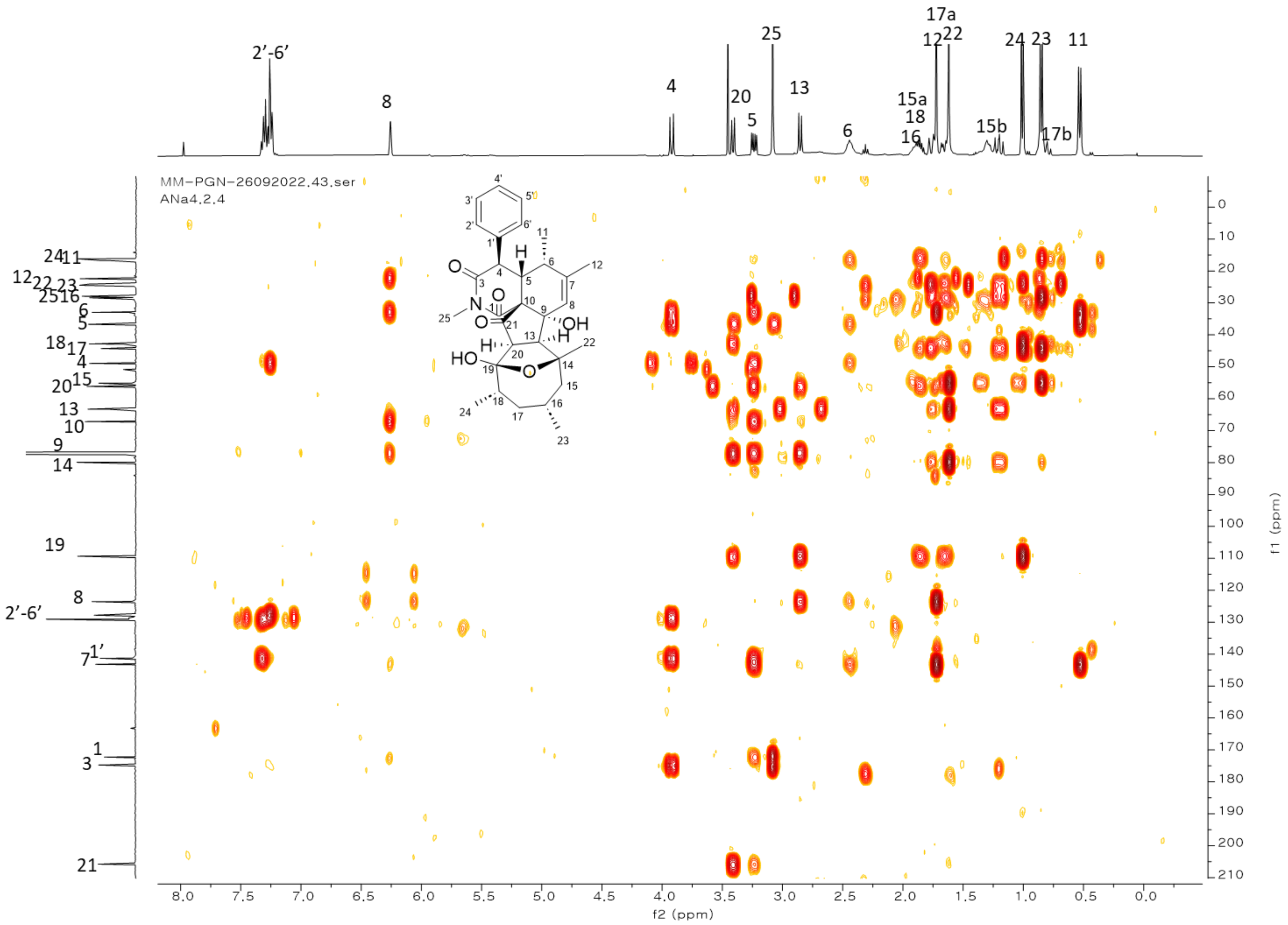

Figure S35. ^{1}H-^{13}C HMBC spectrum of **5** in $CDCl_3$

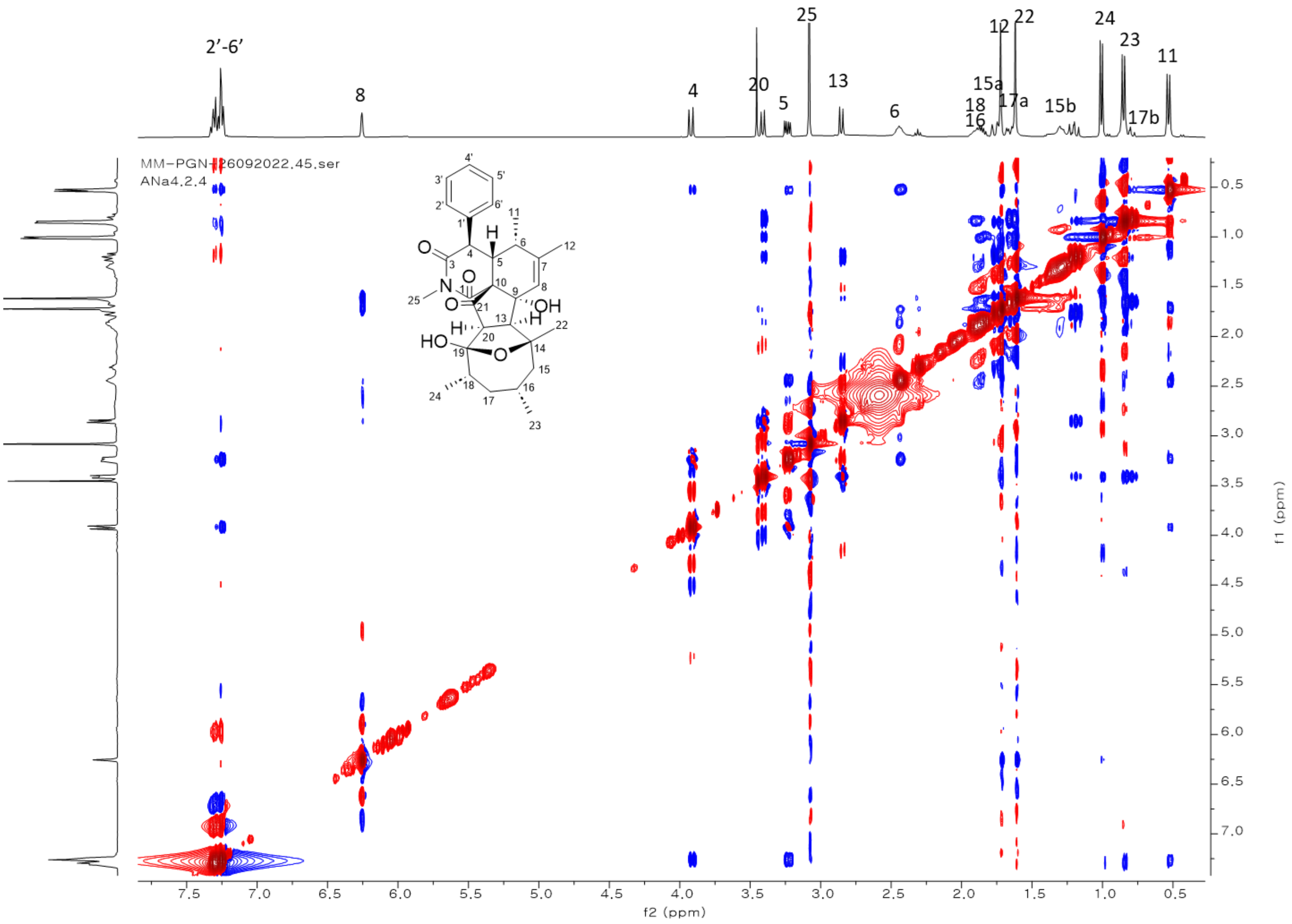

Figure S36. ^1H-^1H NOESY spectrum of **5** in $CDCl_3$

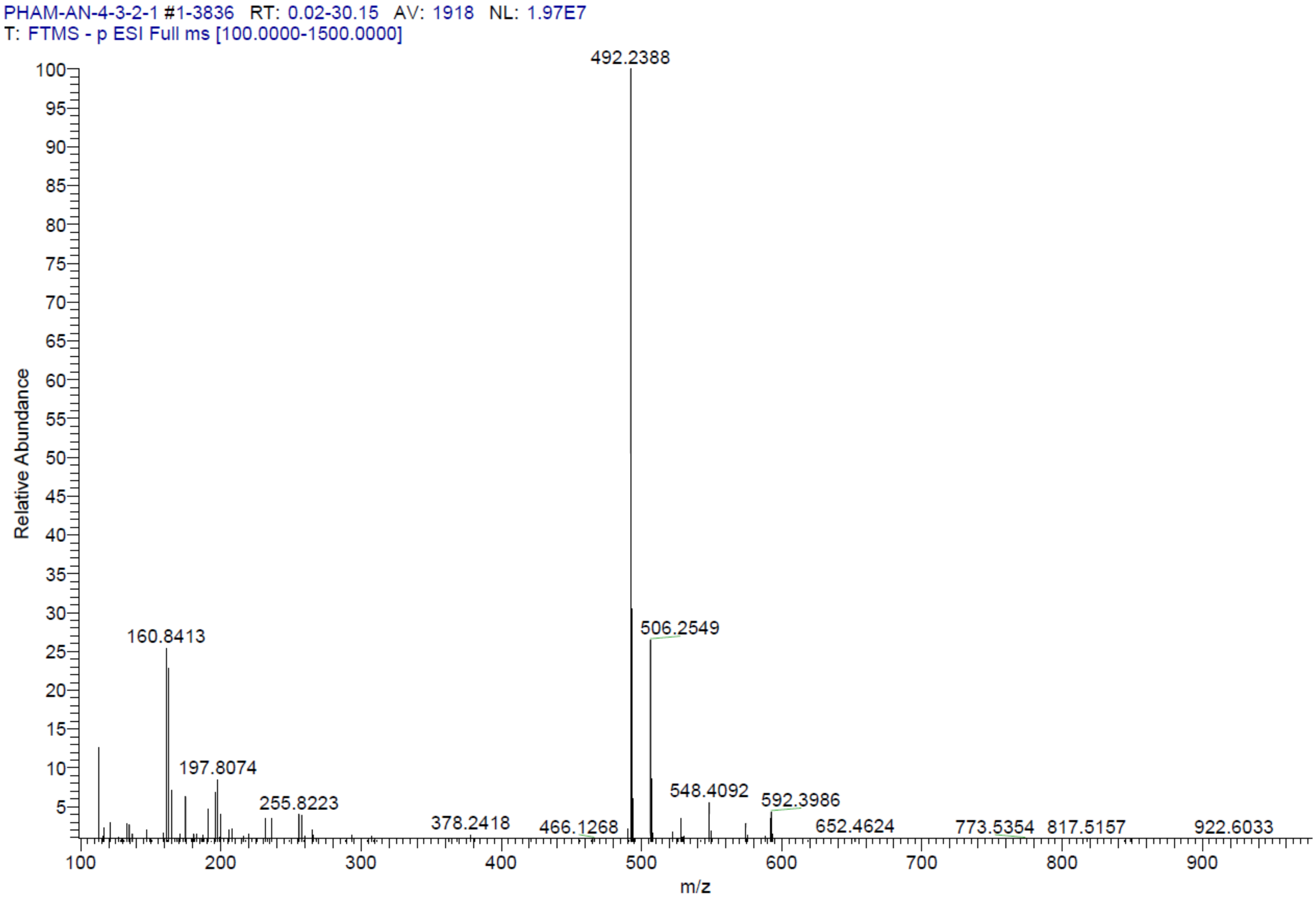

Figure S37. HRESIMS of **6**

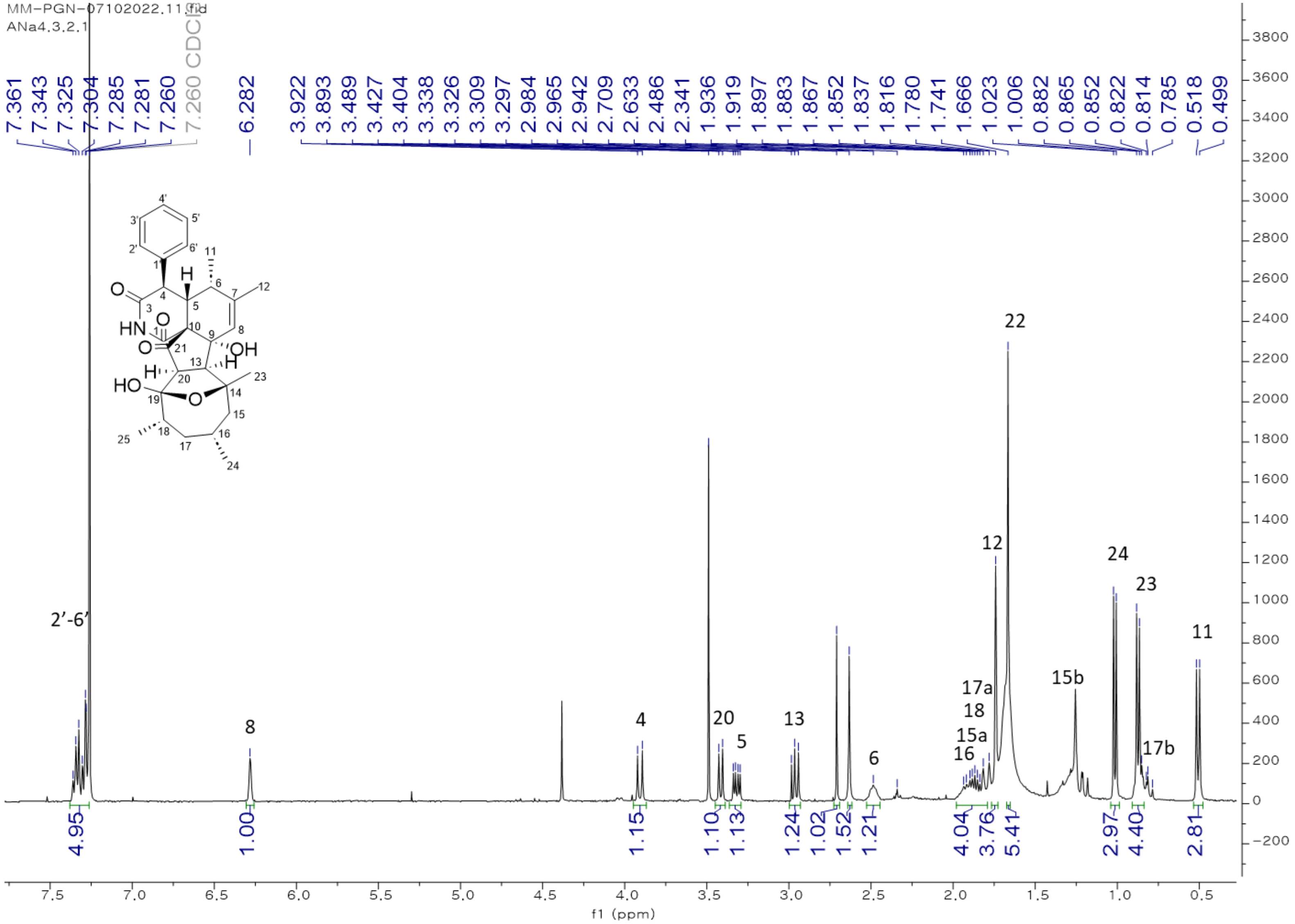

Figure S38. ^{1}H NMR (400 MHz) spectrum of **6** in $CDCl_3$

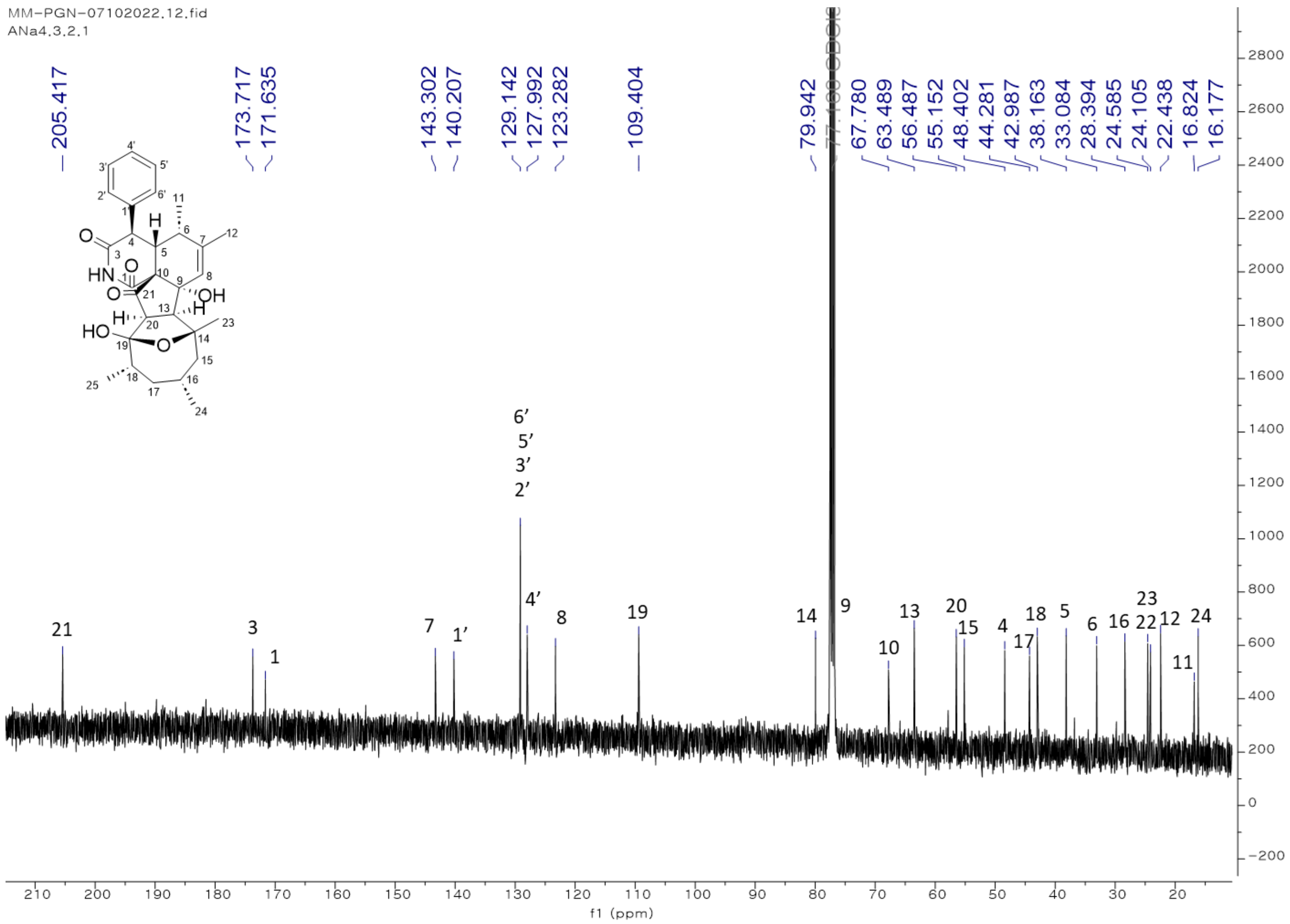

Figure S39. ^{13}C NMR (100 MHz) spectrum of **6** in $CDCl_3$

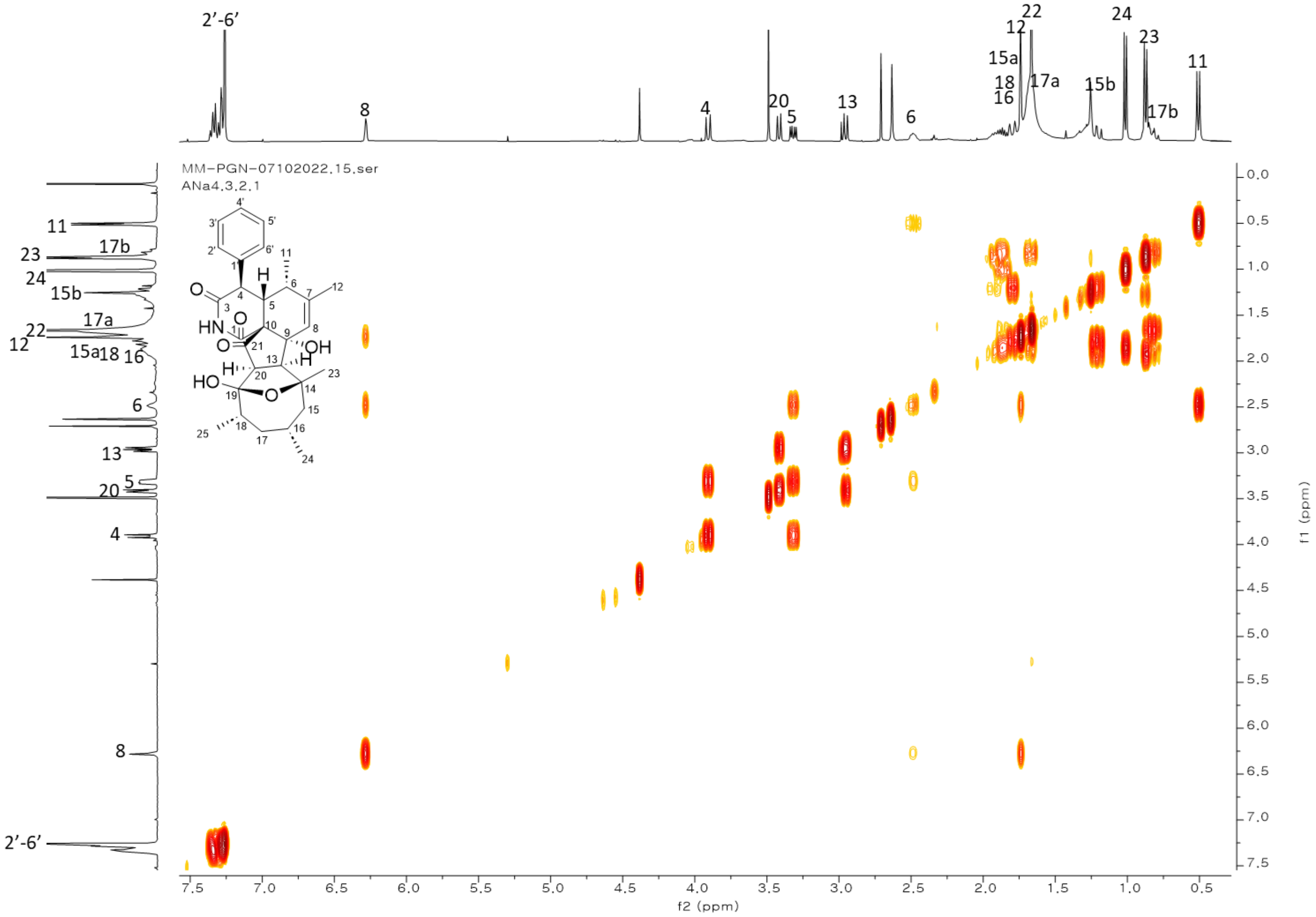

Figure S40. ${}^{1}H$-${}^{1}H$ COSY spectrum of **6** in $CDCl_3$

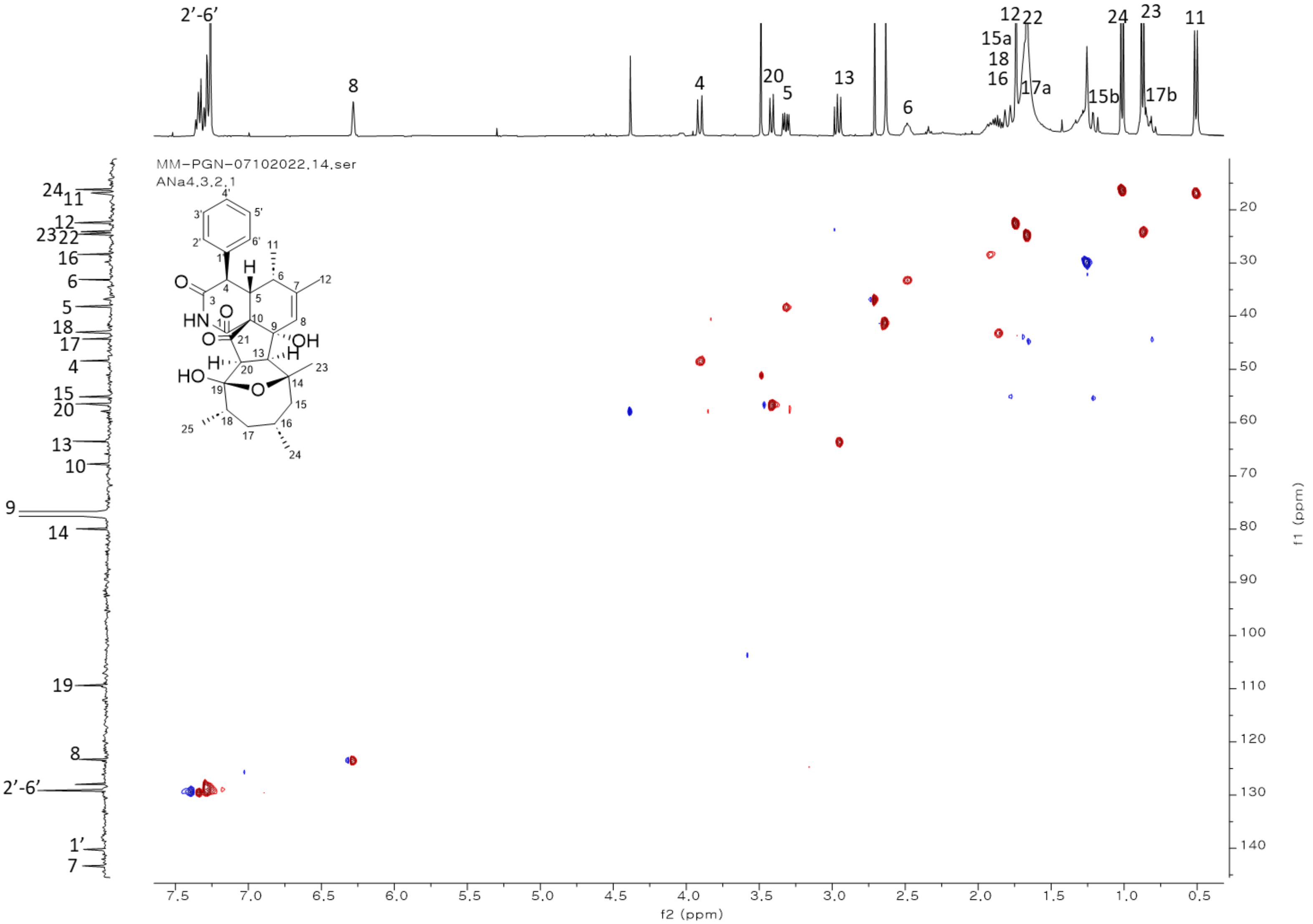

Figure S41. ^{1}H-^{13}C HSQC spectrum of **6** in $CDCl_3$

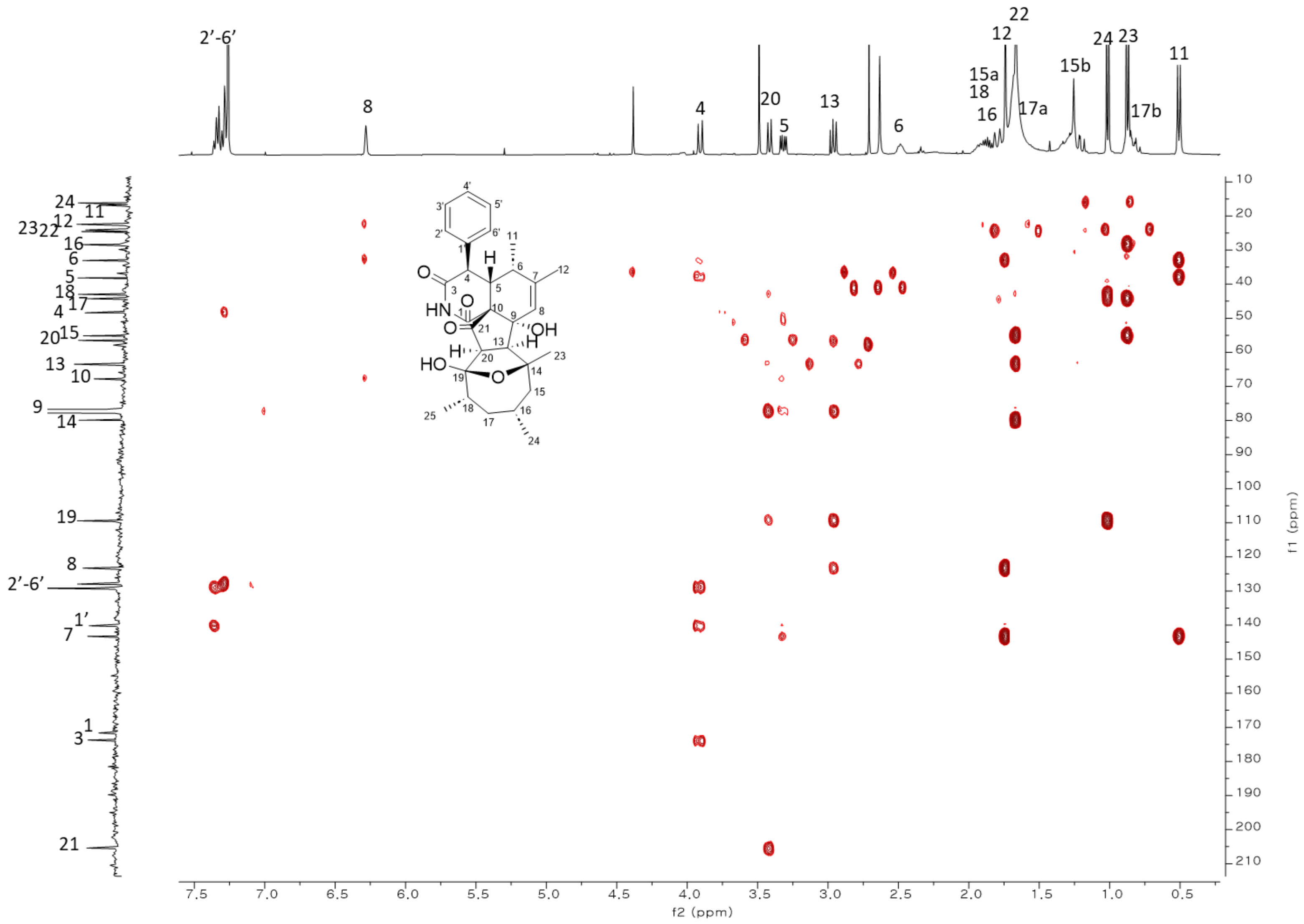

Figure S42. ^{1}H-^{13}C HMBC spectrum of **6** in $CDCl_3$

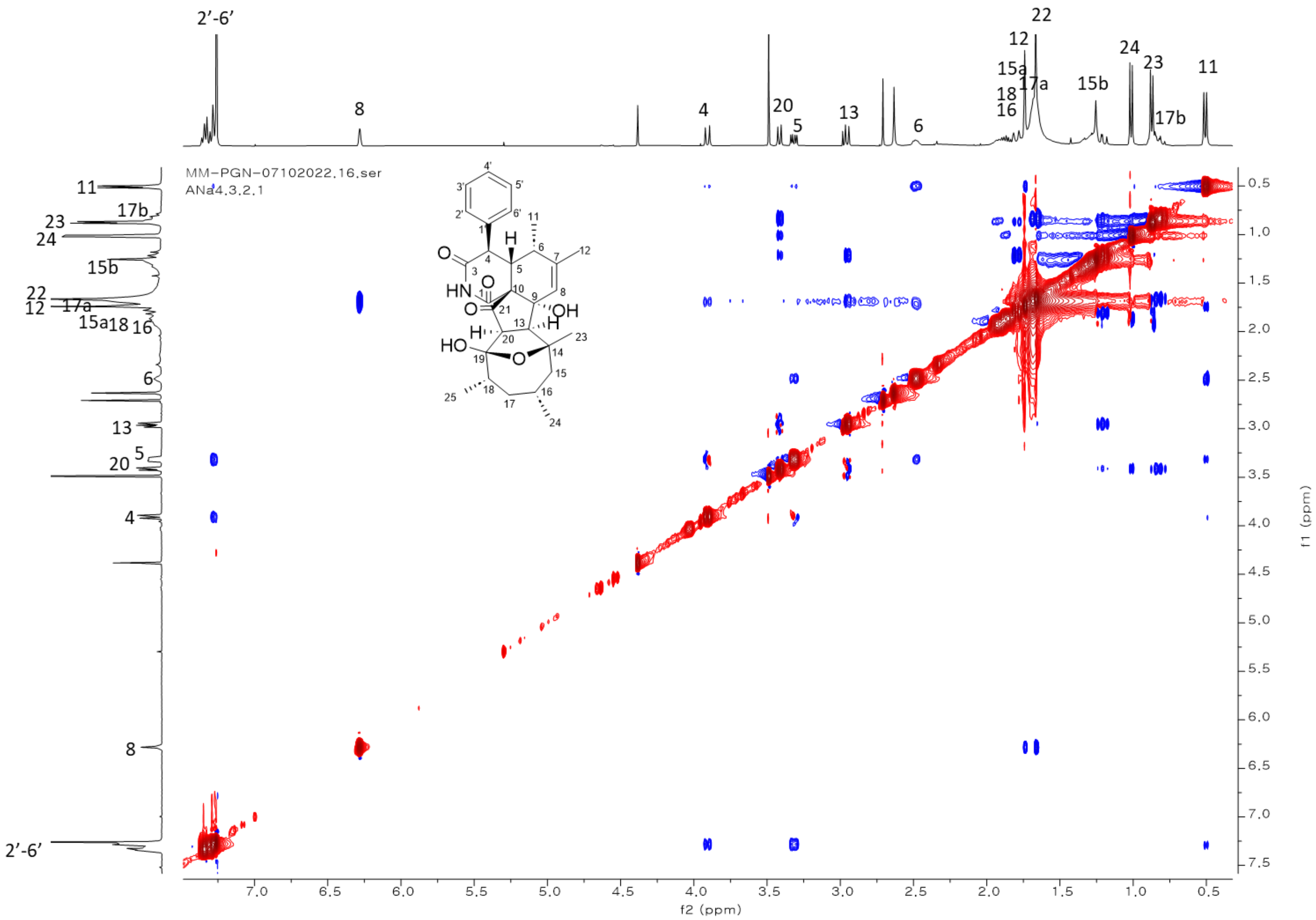

Figure S43. ^{1}H-^{1}H NOESY spectrum of **6** in $CDCl_3$

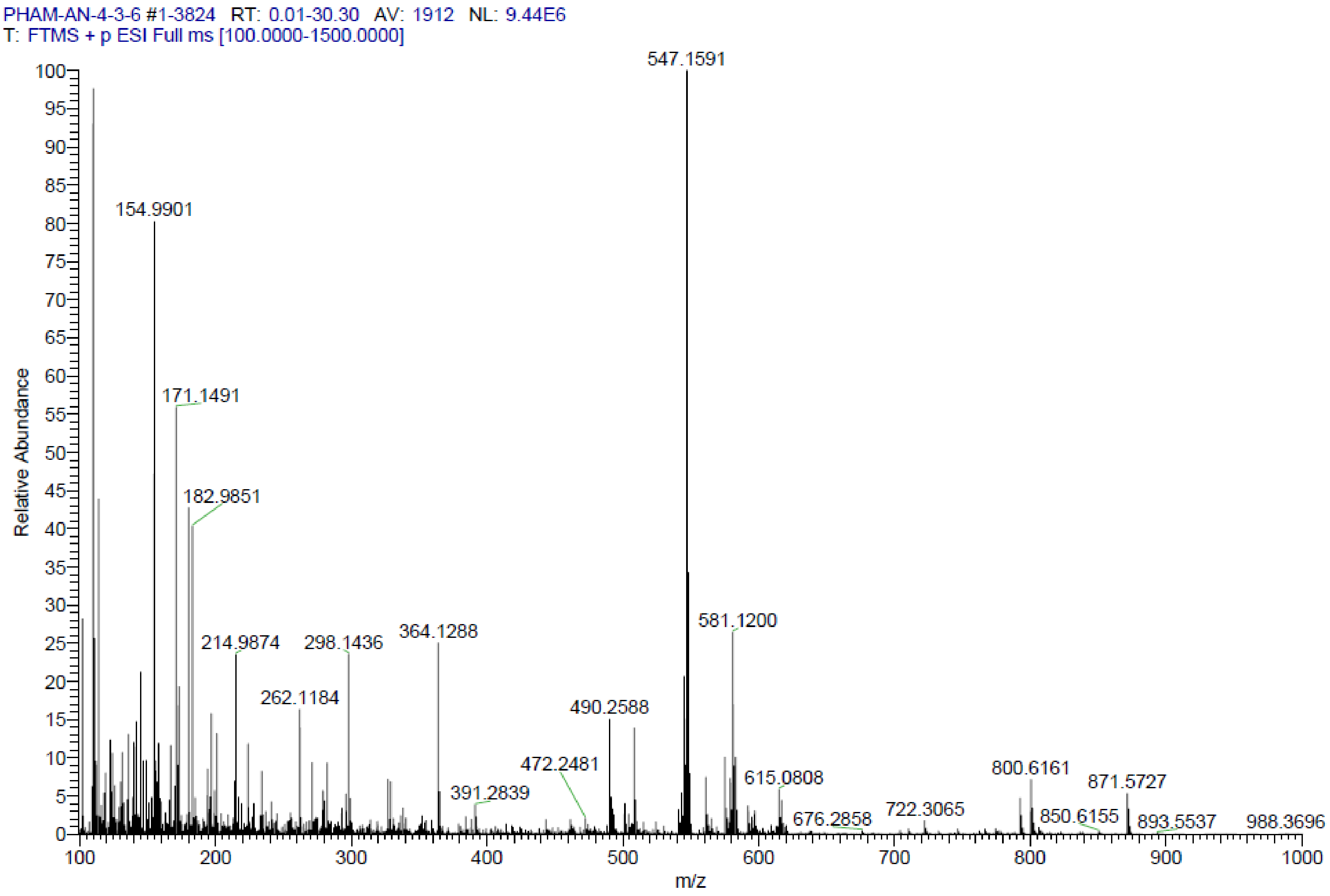

Figure S44. HRESIMS of **7**

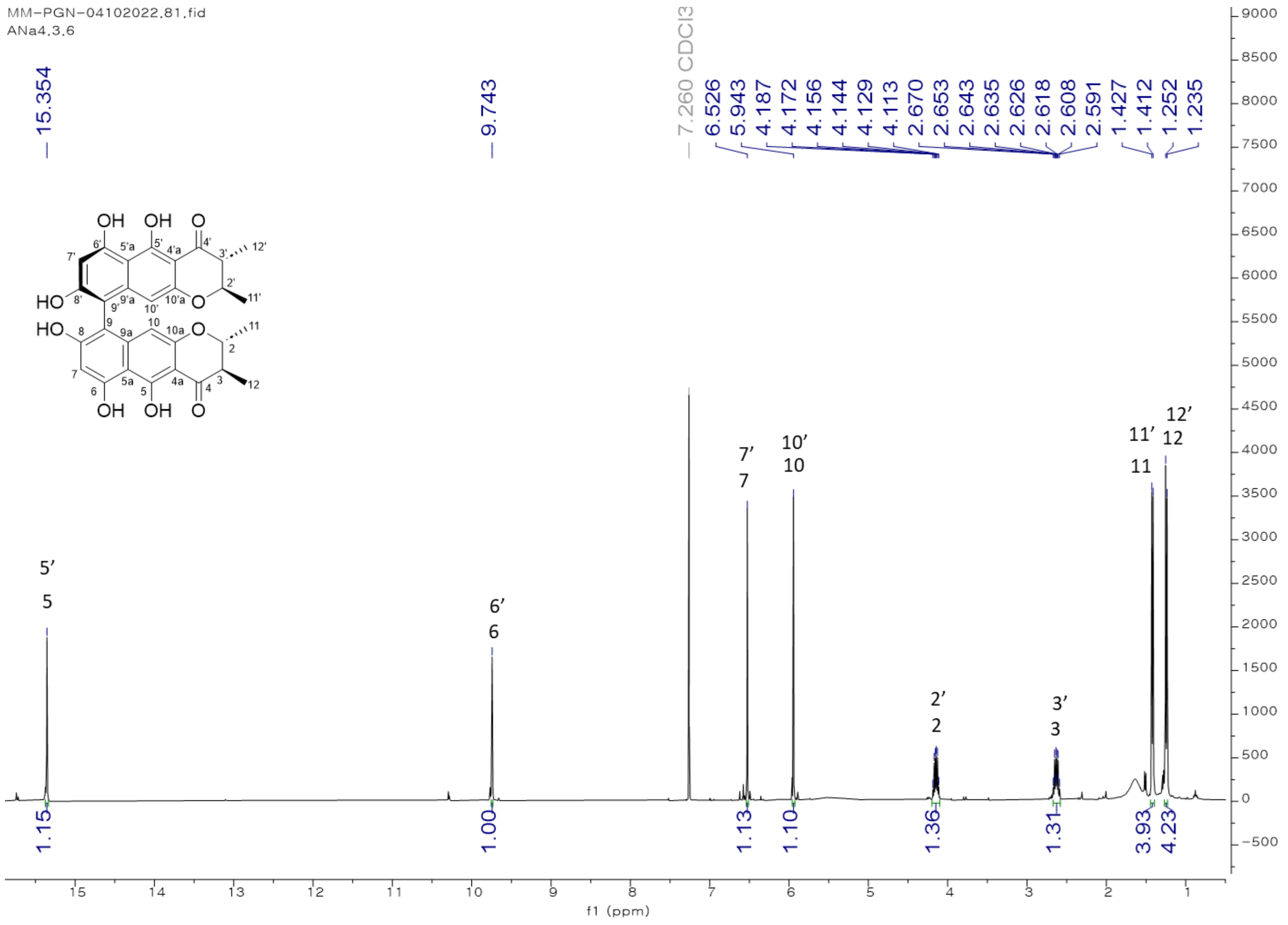

Figure S45. ^{1}H NMR (400 MHz) spectrum of **7** in $CDCl_3$

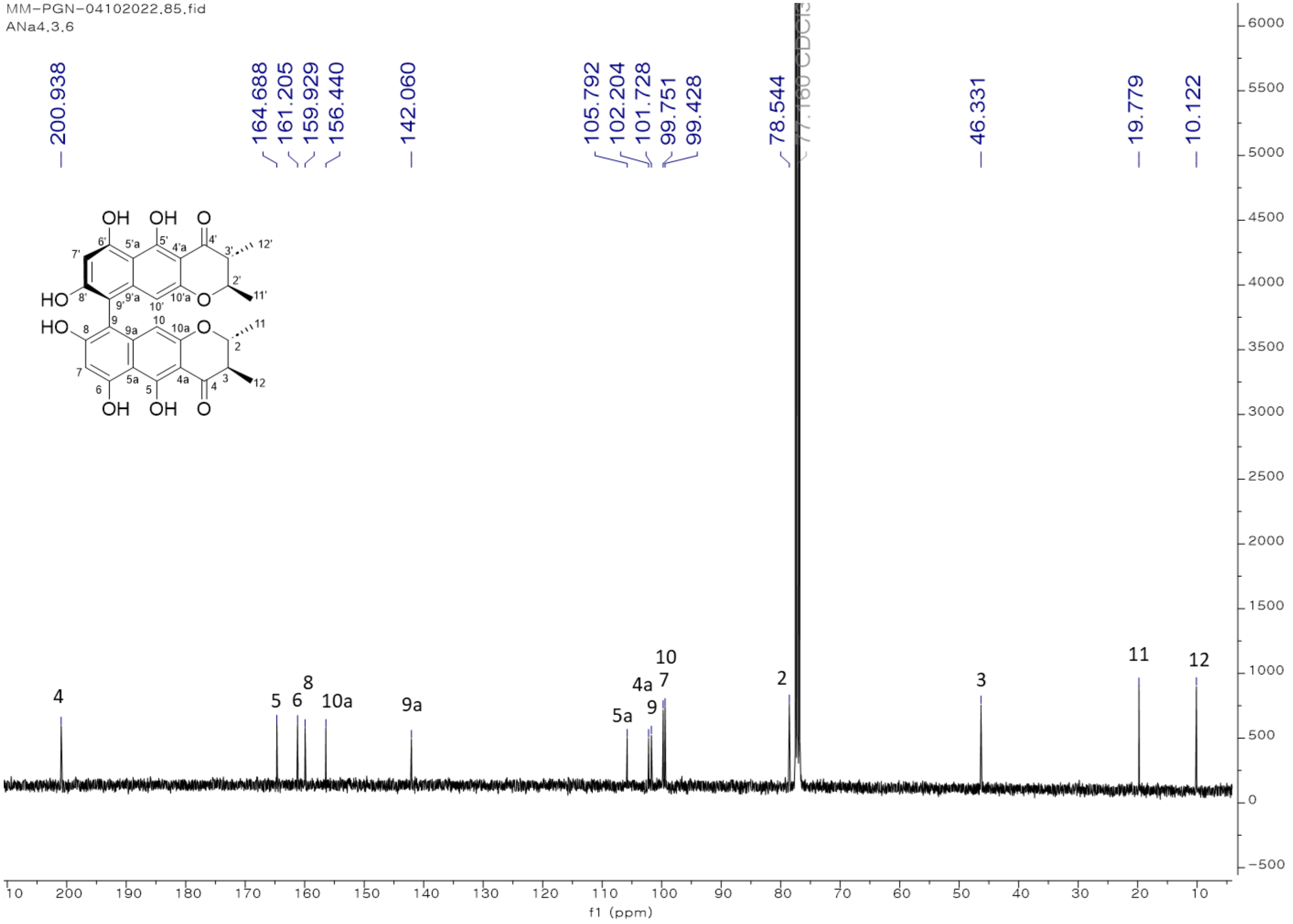

Figure S46. ^{13}C NMR (100 MHz) spectrum of **7** in $CDCl_3$

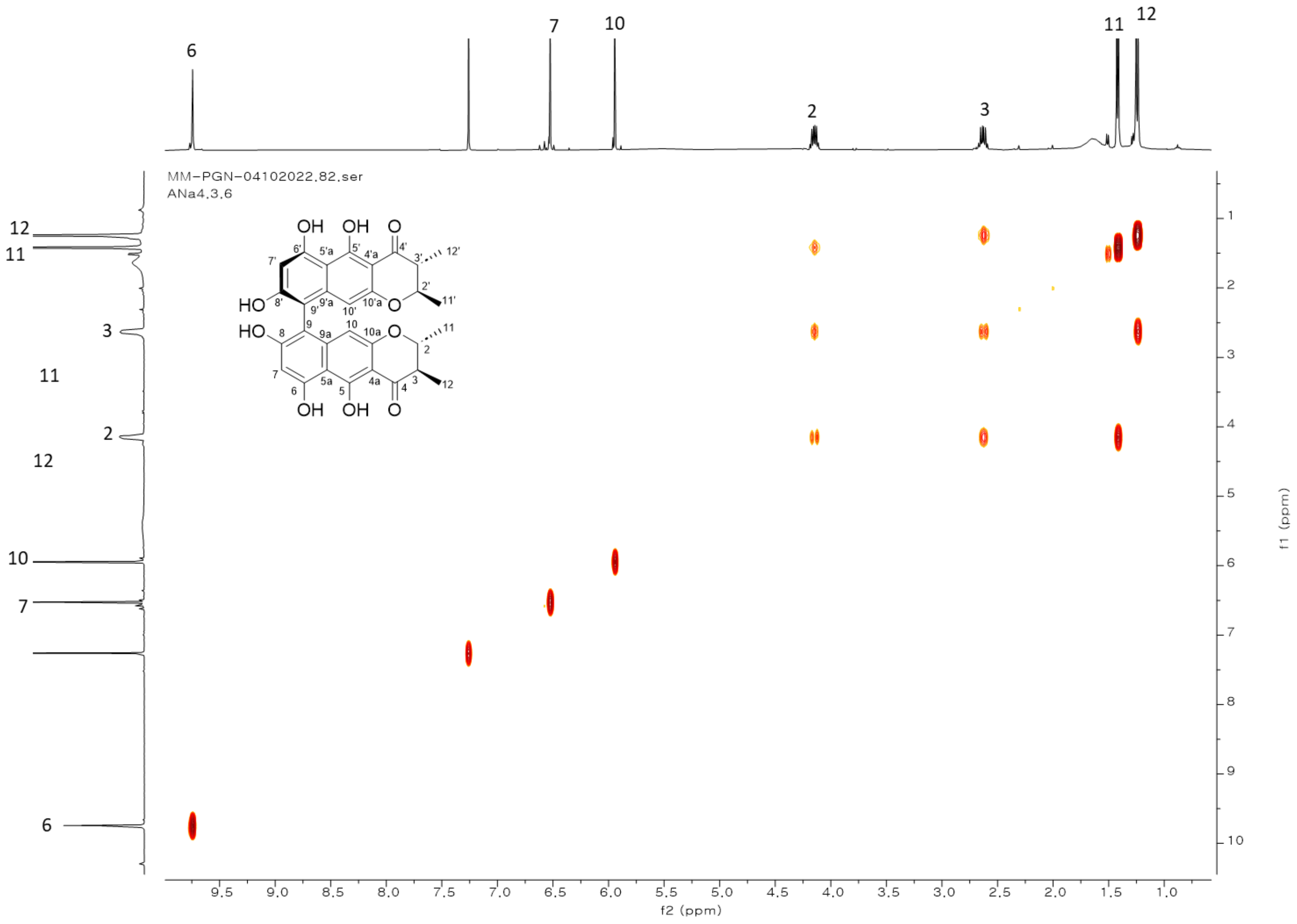

Figure S47. ^{1}H-^{1}H COSY spectrum of **7** in $CDCl_3$

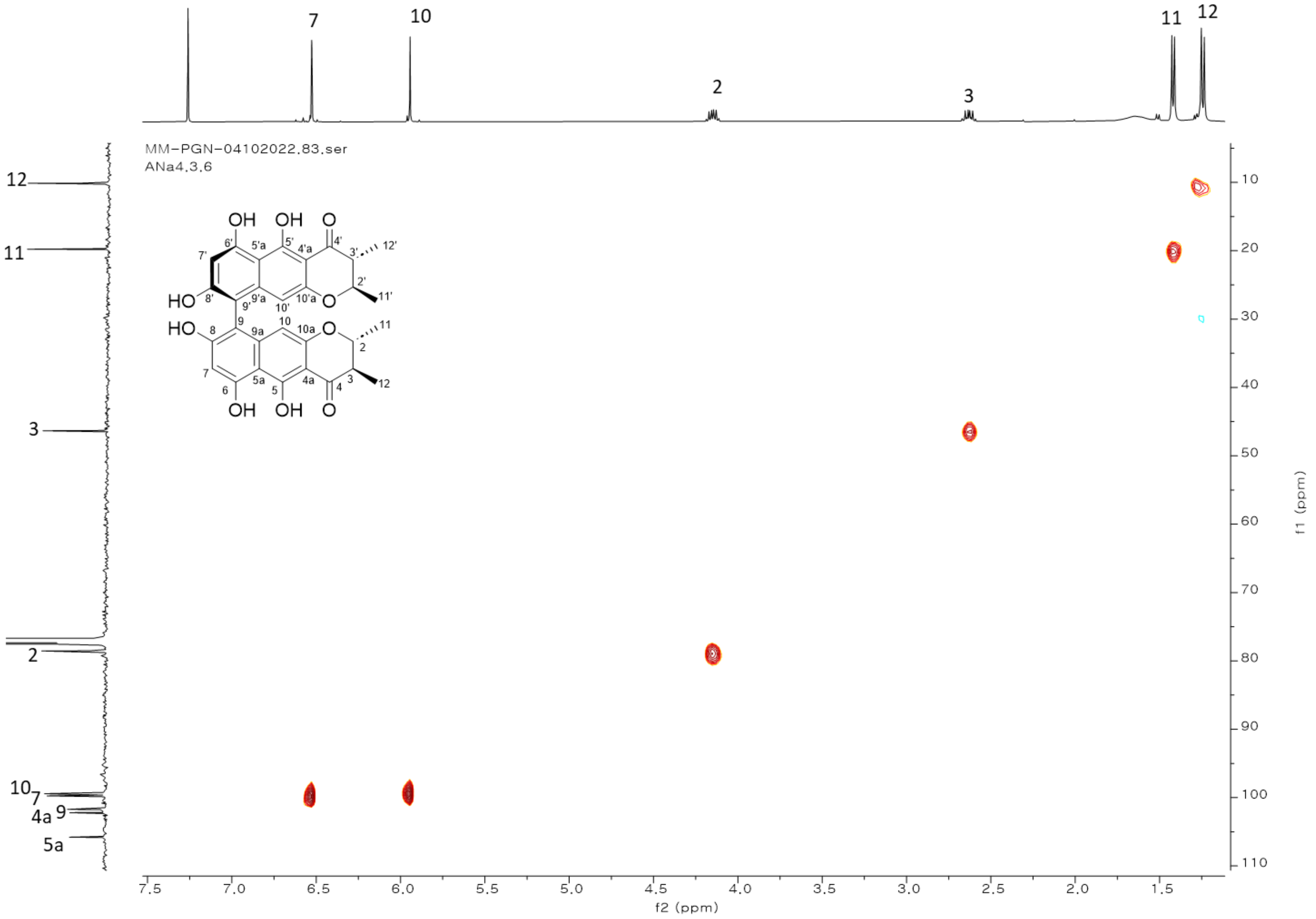

Figure S48. ¹H-¹³C HSQC spectrum of **7** in $CDCl_3$

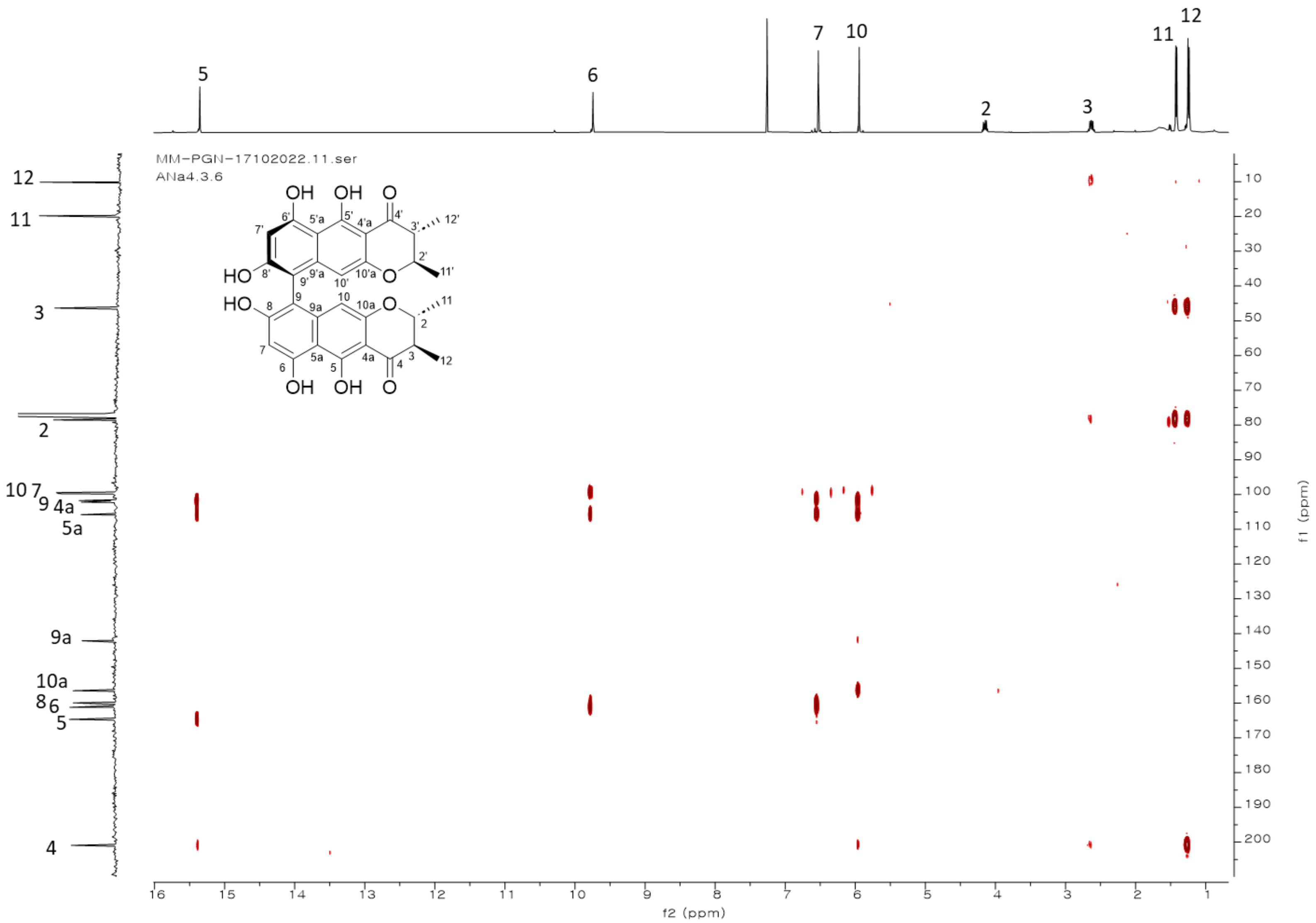

Figure S49. ^{1}H-^{13}C HMBC spectrum of **7** in $CDCl_3$

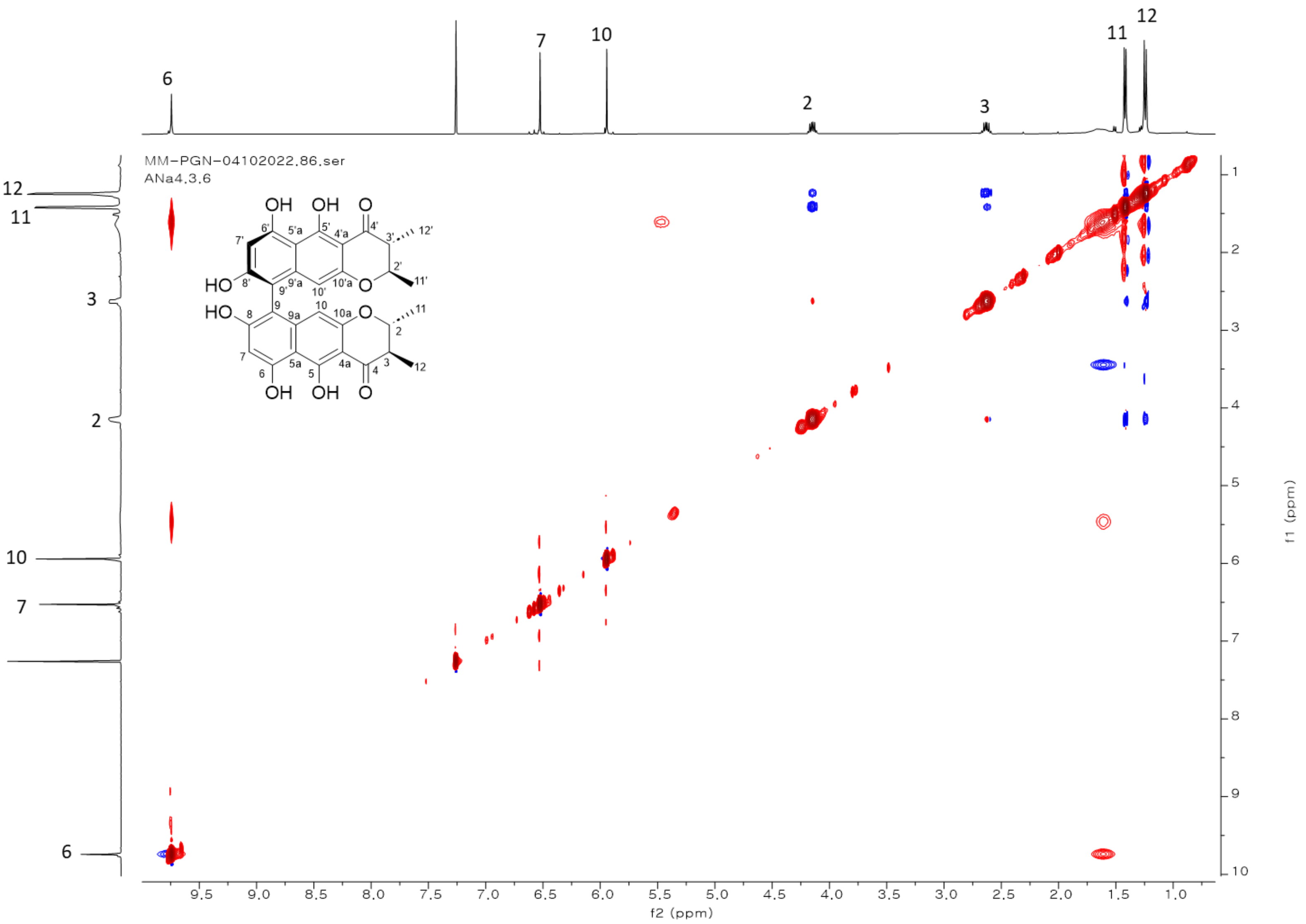

Figure S50. ^{1}H-^{1}H NOESY spectrum of **7** in $CDCl_3$

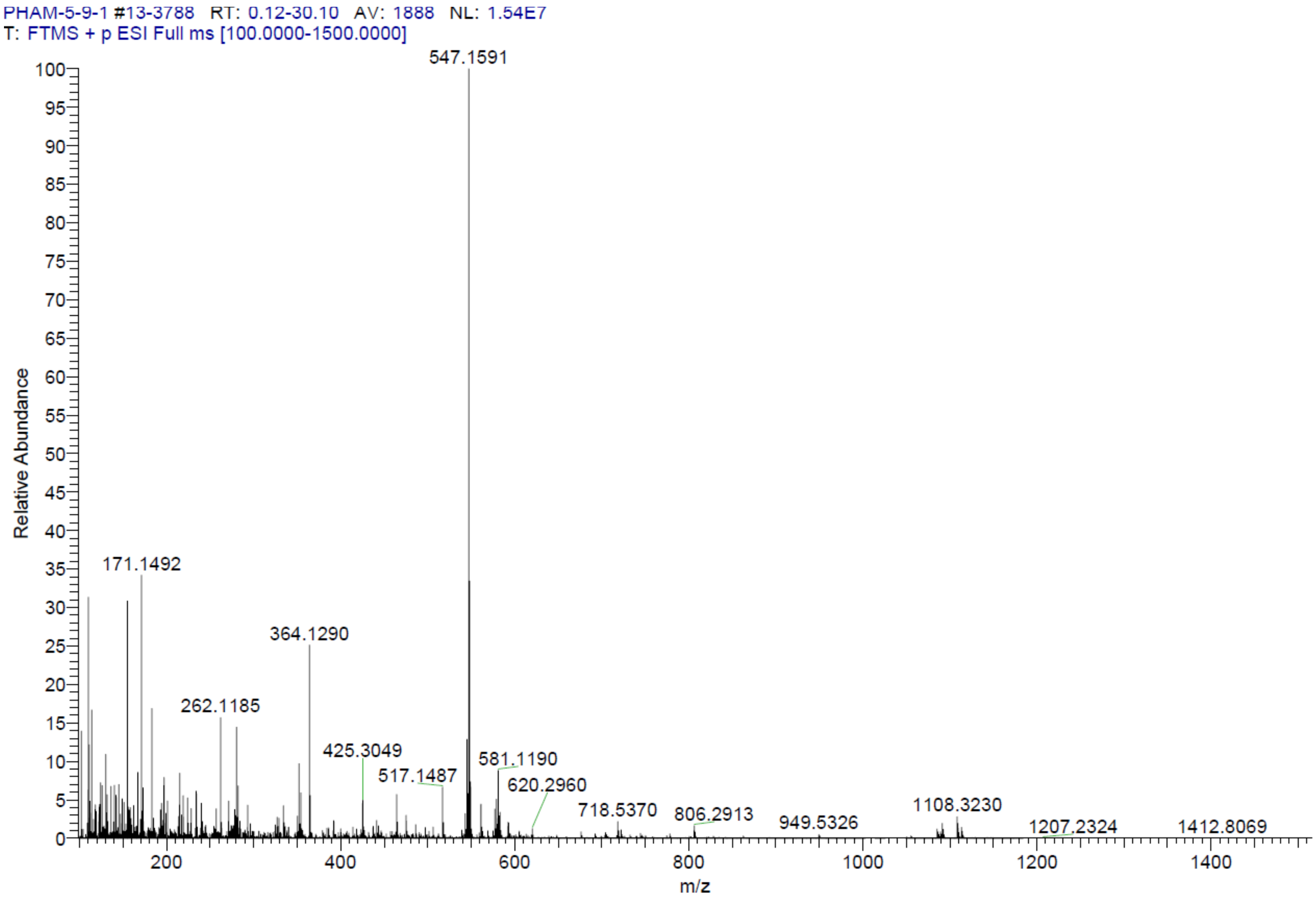

Figure S51. HRESIMS of **8**

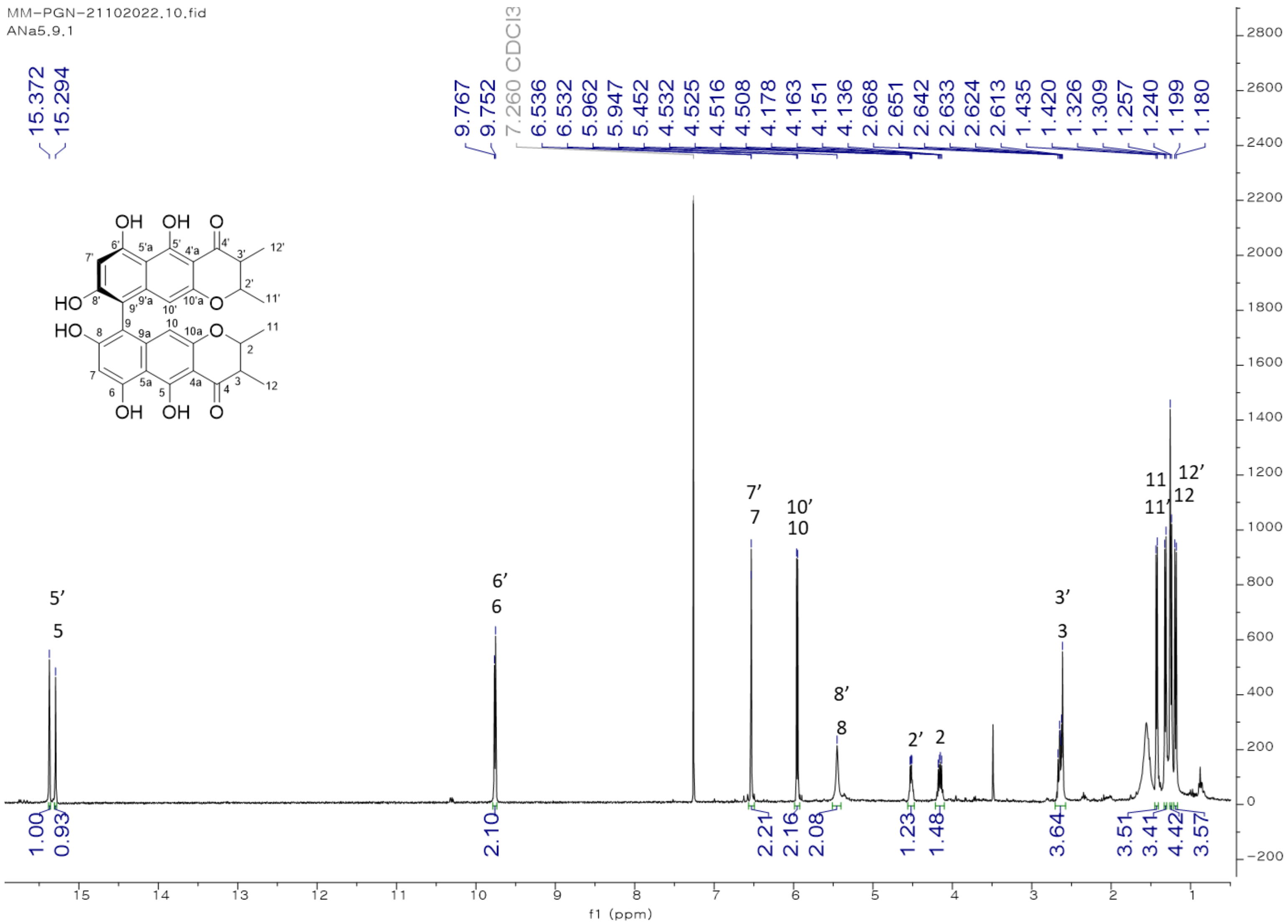

Figure S52. ^{1}H NMR (400 MHz) spectrum of **8** in $CDCl_3$

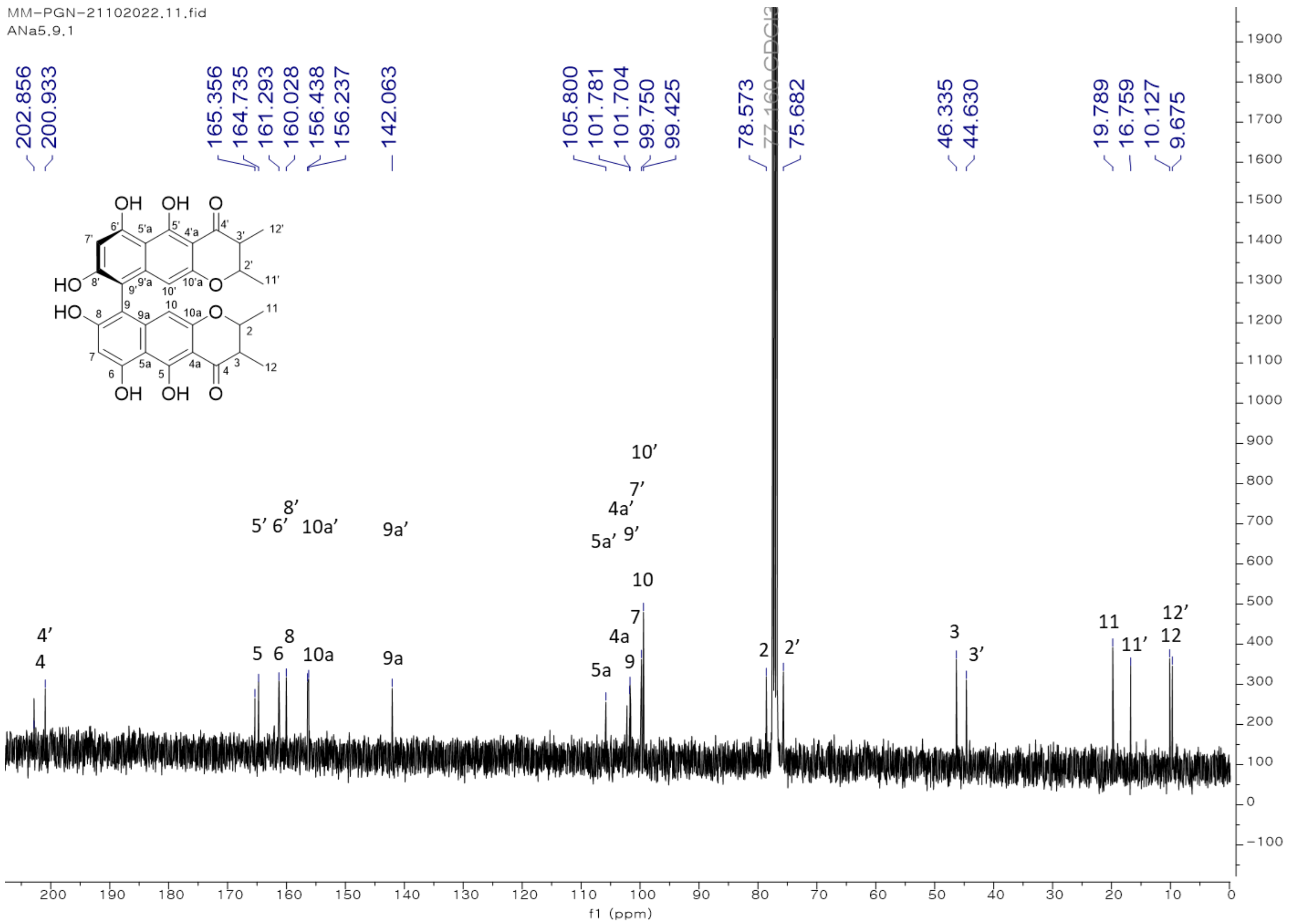

Figure S53. ^{13}C NMR (100 MHz) spectrum of **8** in $CDCl_3$

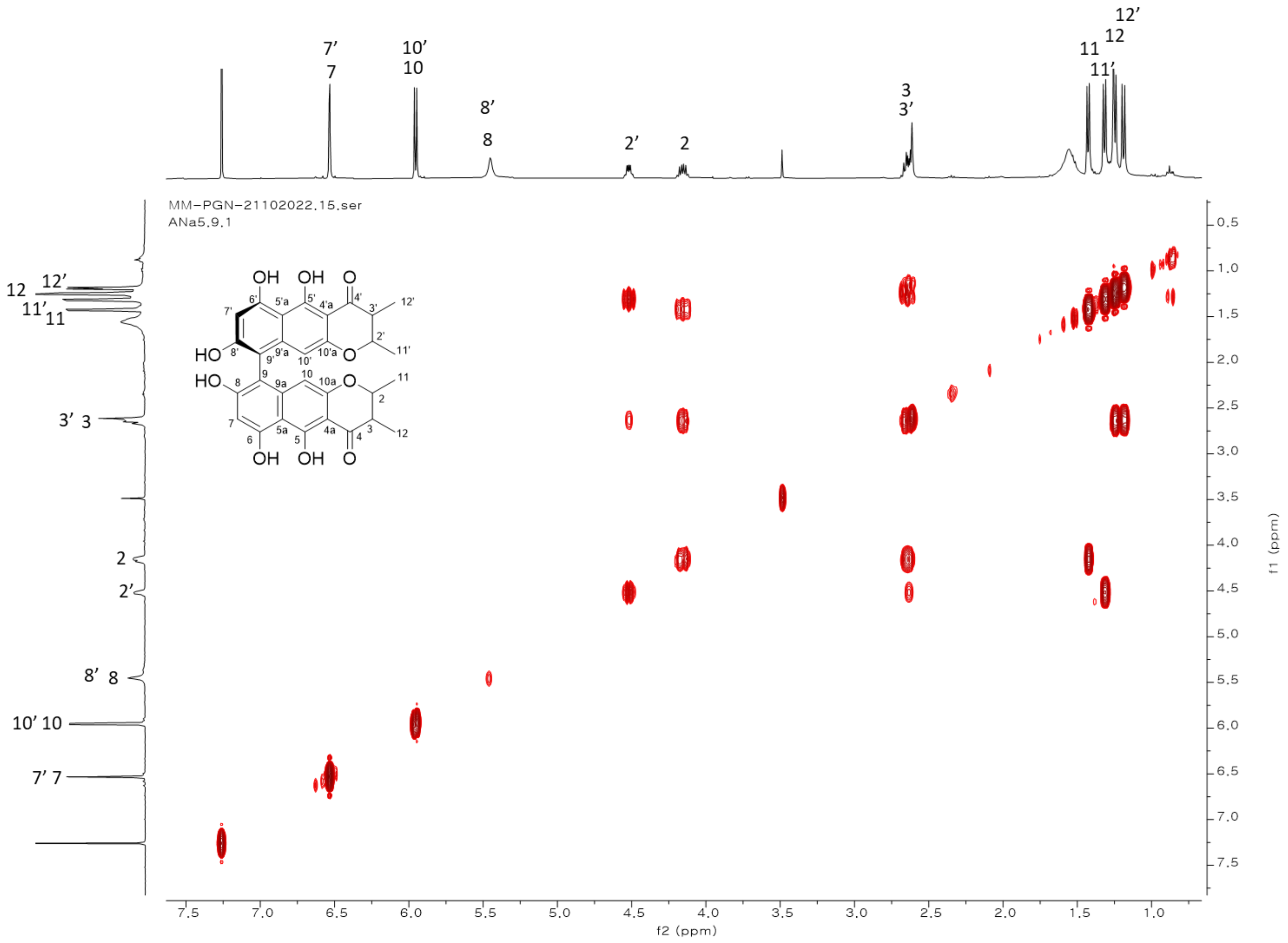

Figure S54. ^{1}H-^{1}H COSY spectrum of **8** in $CDCl_3$

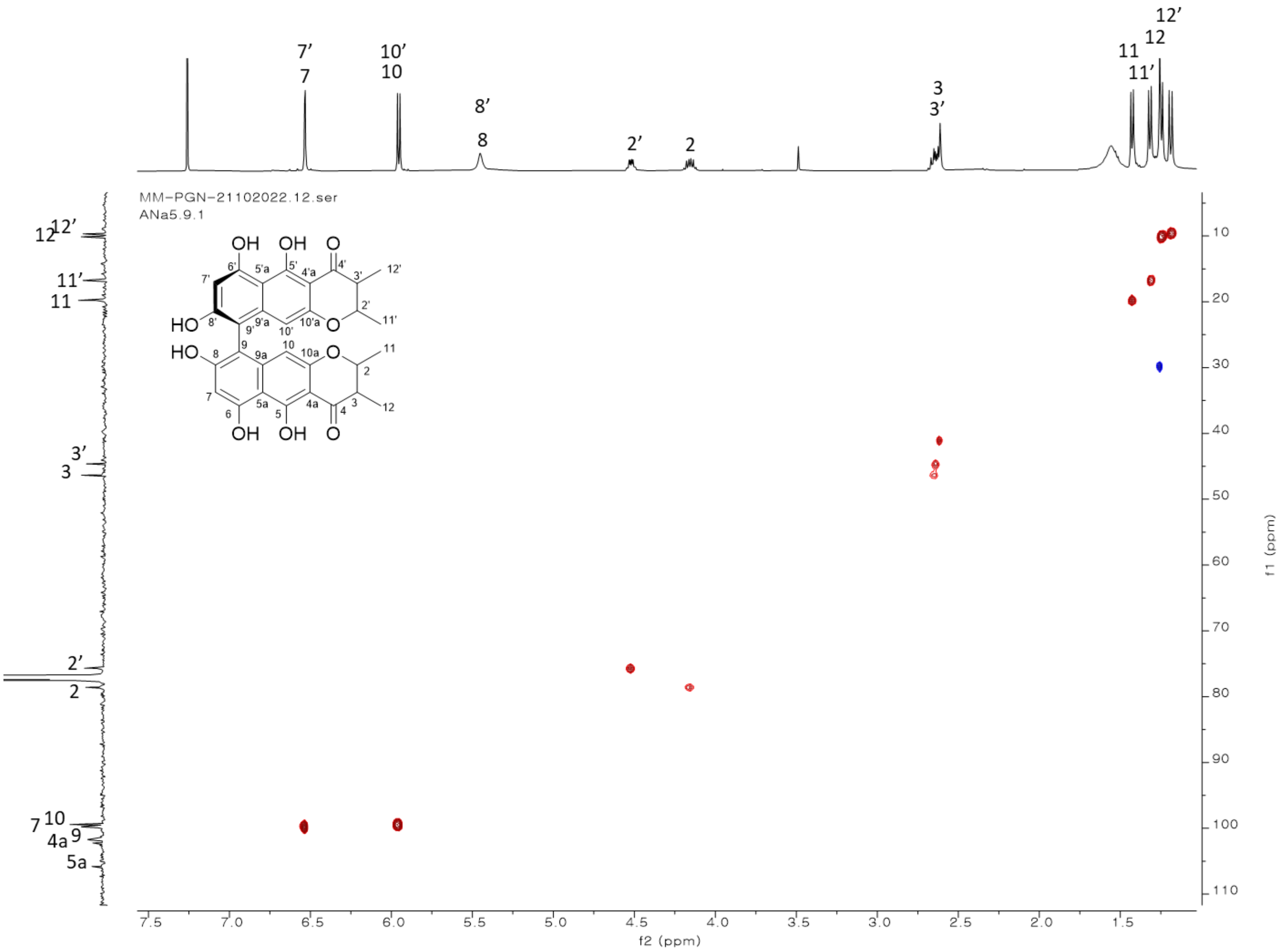

Figure S55. ^{1}H-^{13}C HSQC spectrum of **8** in $CDCl_3$

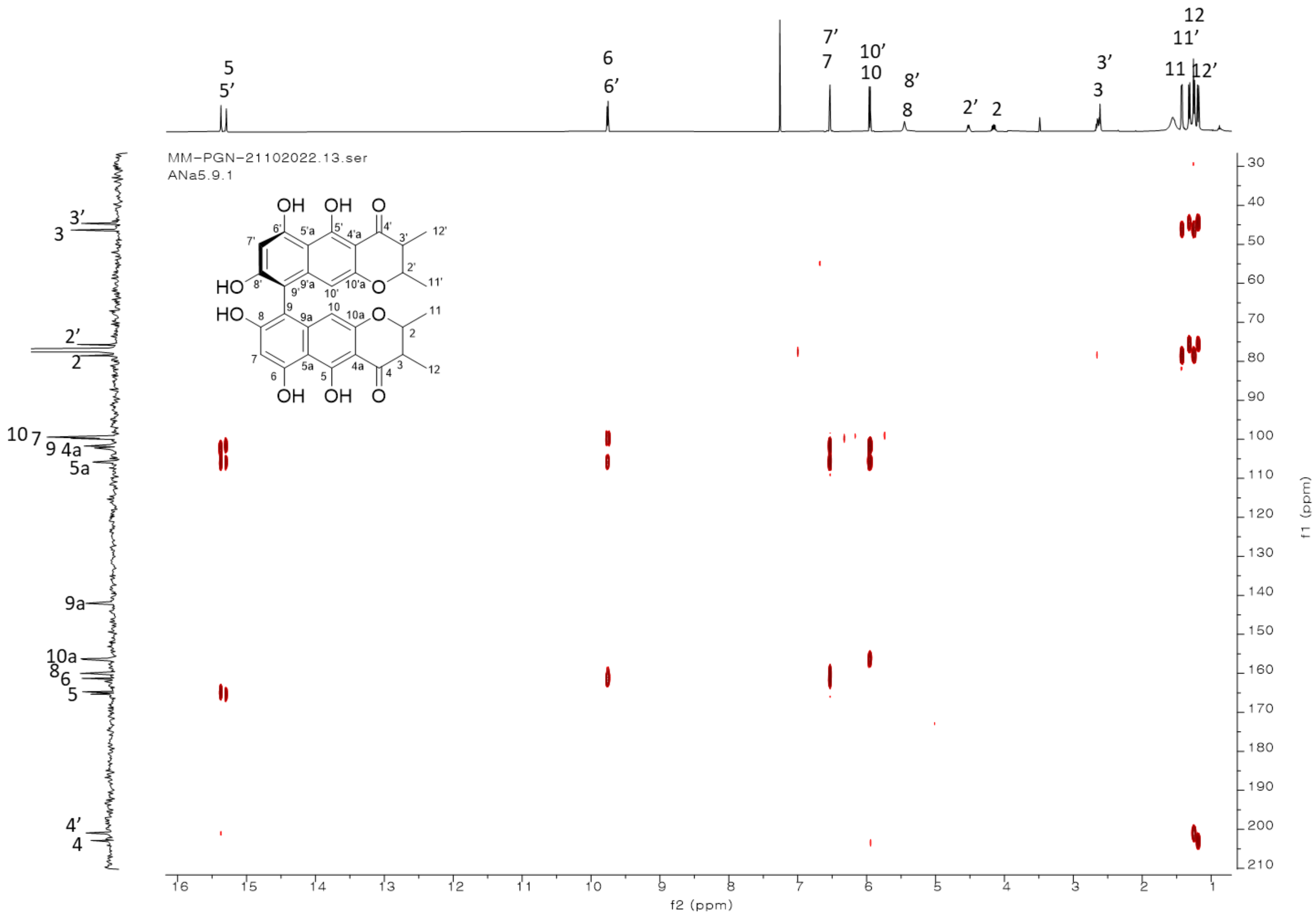

Figure S56. ^1H-^{13}C HMBC spectrum of **8** in $CDCl_3$

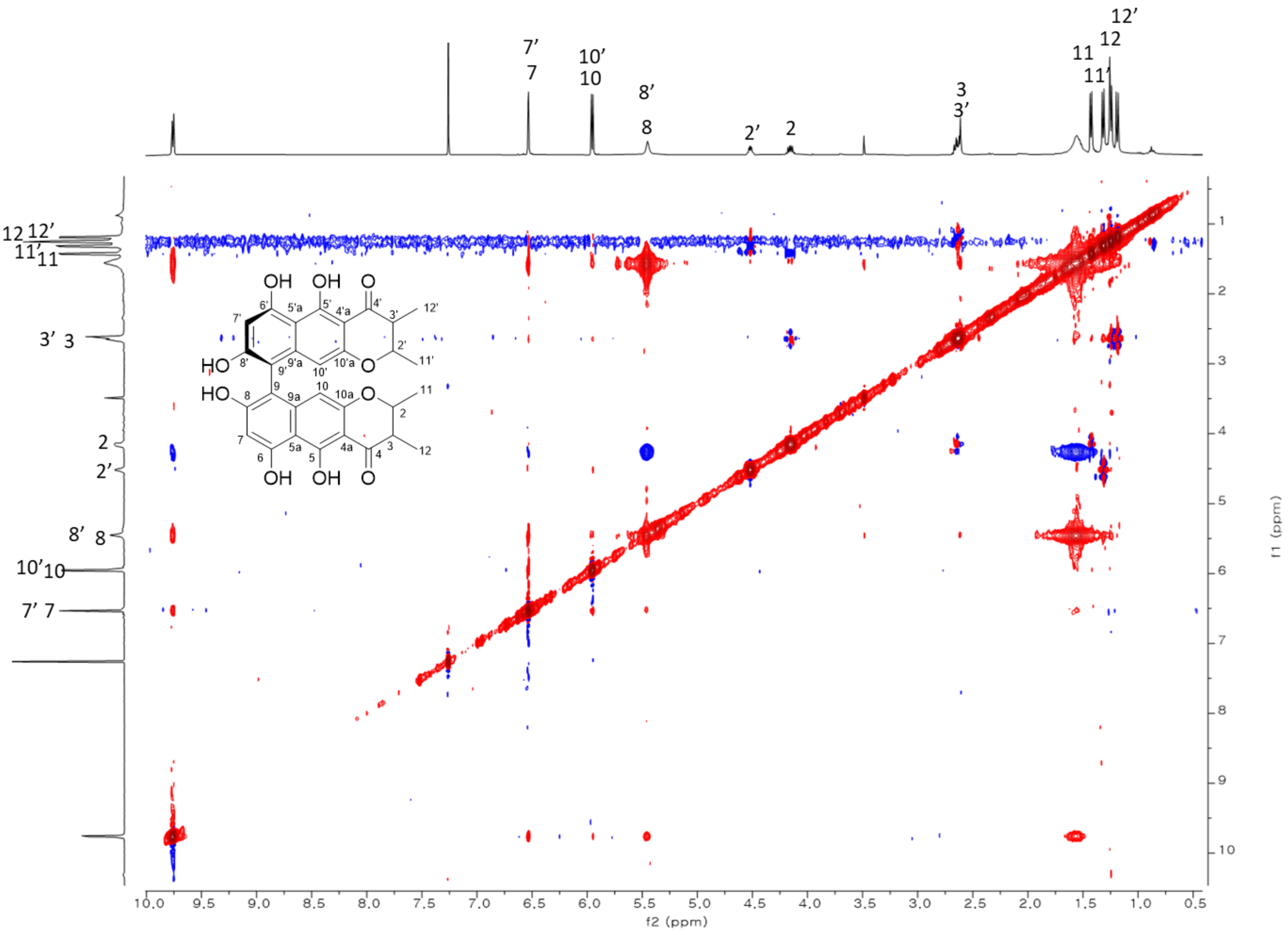

Figure S57. ^{1}H-^{1}H NOESY spectrum of **8** in $CDCl_3$

Table S1. Antimicrobial and cytotoxic activities of compounds **7-8.**

	MIC (μM)				
	Bacteria		Yeasts		Parasites
Compounds	*Escherichia coli* (UTI89)	*Staphylococcus aureus* (S25)	*Candida albicans* (CA01)	*Candida tropicalis* (CK01)	*Leishmania infantum* (LI01)
Chaetochromin A (**7**)	> 100	6.25	> 100	> 100	> 100
Chaetochromin B (**8**)	> 100	6.25	100	> 100	> 100
Ampicillin (reference)	> 100	1.56	-	-	-
Amphothericin B (reference)	-	-	0.20	0.20	0.39
DMSO (solvent)	> 100	> 100	> 100	> 100	> 100

	IC_{50} (μM)		
	**Cell line**	**Primary cells**	
**Compounds**	**THP-1**	**RBC**	**PBMC**
Chaetochromin A (**7**)	33.65	> 200	35.35
Chaetochromin B (**8**)	37.34	> 200	40.01
Ampicillin (reference)	> 200	> 200	> 200
Amphotericin B (reference)	3.13	25	6.25
DMSO (solvent)	> 200	> 200	> 200

Table S2. Gibbs Free Energy and Boltzmann Population of occurring conformers of isomer **1a – 8*R*, 9*R*, 10*R*, 12*S*, 14*S***

Conformer's filenames	Energy (a.u.)	Relative Energy (kcal/mol)	Boltzmann Factor	Equilibrium Mole Fraction	Number of Imaginary Frequencies
1a-6.out	-1272.408653	0	1	0.129296	0
1a-39.out	-1272.408649	0.00251	0.995768	0.128749	0
1a-1.out	-1272.408646	0.004393	0.992605	0.12834	0
1a-2.out	-1272.408589	0.040161	0.934393	0.120813	0
1a-5.out	-1272.408582	0.044553	0.927483	0.11992	0
1a-37.out	-1272.40858	0.045808	0.925518	0.119666	0
1a-17.out	-1272.408231	0.264809	0.639261	0.082654	0
1a-8.out	-1272.407649	0.630019	0.344889	0.044593	0
1a-3.out	-1272.407641	0.63504	0.341976	0.044216	0
1a-7.out	-1272.407428	0.768699	0.272844	0.035278	0
1a-4.out	-1272.40742	0.773719	0.270539	0.03498	0
1a-21.out	-1272.404119	2.845128	0.008169	0.001056	0
1a-22.out	-1272.404104	2.85454	0.008041	0.00104	0
1a-10.out	-1272.4041	2.85705	0.008007	0.001035	0
1a-9.out	-1272.404097	2.858933	0.007981	0.001032	0
1a-20.out	-1272.403832	3.025223	0.006026	0.000779	0
1a-12.out	-1272.403831	3.02585	0.00602	0.000778	0
1a-19.out	-1272.403802	3.044048	0.005837	0.000755	0
1a-11.out	-1272.403801	3.044676	0.005831	0.000754	0
1a-29.out	-1272.403455	3.261794	0.00404	0.000522	0
1a-33.out	-1272.403451	3.264304	0.004023	0.00052	0
1a-28.out	-1272.403208	3.416789	0.00311	0.000402	0
1a-13.out	-1272.402882	3.621357	0.002201	0.000285	0
1a-25.out	-1272.402882	3.621357	0.002201	0.000285	0
1a-15.out	-1272.40288	3.622612	0.002196	0.000284	0
1a-27.out	-1272.402875	3.625749	0.002185	0.000282	0
1a-26.out	-1272.402842	3.646457	0.002109	0.000273	0
1a-18.out	-1272.402827	3.65587	0.002076	0.000268	0

1a-23.out	-1272.402827	3.65587	0.002076	0.000268	0
1a-14.out	-1272.402826	3.656497	0.002074	0.000268	0
1a-32.out	-1272.402363	3.947034	0.001269	0.000164	0
1a-41.out	-1272.402242	4.022963	0.001117	0.000144	0
1a-35.out	-1272.402058	4.138424	0.000919	0.000119	0
1a-42.out	-1272.402026	4.158505	0.000888	0.000115	0
1a-40.out	-1272.400448	5.148715	0.000167	2.15E-05	0
1a-36.out	-1272.400447	5.149342	0.000166	2.15E-05	0
1a-16.out	-1272.400304	5.239076	0.000143	1.85E-05	0
1a-24.out	-1272.39938	5.818895	5.37E-05	6.94E-06	0
1a-31.out	-1272.395906	7.998862	1.35E-06	1.75E-07	0
1a-30.out	-1272.395905	7.99949	1.35E-06	1.74E-07	0
1a-34.out	-1272.394922	8.616331	4.76E-07	6.15E-08	0
1a-38.out	-1272.394919	8.618214	4.74E-07	6.13E-08	0

Table S3. Gibbs Free Energy and Boltzmann Population of occurring conformers of isomer **1b – 8*S*, 9*S*, 10*S*, 12*R*, 14*R***

Conformer's filenames	Energy (a.u.)	Relative Energy (kcal/mol)	Boltzmann Factor	Equilibrium Mole Fraction	Number of Imaginary Frequencies
1b-6.out	-1272.408652	0	1	0.125378	0
1b-1.out	-1272.40864	0.00753	0.987357	0.123793	0
1b-40.out	-1272.40864	0.00753	0.987357	0.123793	0
1b-2.out	-1272.408596	0.035141	0.942352	0.11815	0
1b-5.out	-1272.408584	0.042671	0.930438	0.116656	0
1b-39.out	-1272.408579	0.045808	0.925518	0.116039	0
1b-17.out	-1272.408241	0.257906	0.64676	0.081089	0
1b-8.out	-1272.407649	0.629392	0.345255	0.043287	0
1b-3.out	-1272.407642	0.633784	0.342702	0.042967	0
1b-4.out	-1272.407439	0.761169	0.276338	0.034647	0
1b-7.out	-1272.407424	0.770582	0.271977	0.0341	0
1b-25.out	-1272.407234	0.889808	0.222352	0.027878	0
1b-21.out	-1272.404119	2.8445	0.008178	0.001025	0
1b-10.out	-1272.4041	2.856423	0.008015	0.001005	0
1b-9.out	-1272.404098	2.857678	0.007998	0.001003	0
1b-22.out	-1272.404097	2.858305	0.00799	0.001002	0
1b-36.out	-1272.404067	2.877131	0.007739	0.00097	0
1b-20.out	-1272.403827	3.027733	0.006001	0.000752	0
1b-11.out	-1272.403817	3.034008	0.005937	0.000744	0
1b-12.out	-1272.403811	3.037773	0.0059	0.00074	0
1b-19.out	-1272.403806	3.040911	0.005868	0.000736	0
1b-30.out	-1272.403452	3.263049	0.004032	0.000506	0
1b-34.out	-1272.403449	3.264931	0.004019	0.000504	0
1b-29.out	-1272.40321	3.414906	0.003119	0.000391	0
1b-26.out	-1272.402883	3.620102	0.002205	0.000277	0
1b-28.out	-1272.402883	3.620102	0.002205	0.000277	0
1b-13.out	-1272.40288	3.621984	0.002198	0.000276	0
1b-15.out	-1272.402876	3.624494	0.002189	0.000274	0
1b-27.out	-1272.402843	3.645202	0.002114	0.000265	0
1b-18.out	-1272.402828	3.654615	0.002081	0.000261	0

1b-23.out	-1272.402827	3.655242	0.002078	0.000261	0
1b-14.out	-1272.402826	3.65587	0.002076	0.00026	0
1b-33.out	-1272.40236	3.948289	0.001267	0.000159	0
1b-42.out	-1272.40224	4.02359	0.001115	0.00014	0
1b-37.out	-1272.402029	4.155995	0.000892	0.000112	0
1b-43.out	-1272.402025	4.158505	0.000888	0.000111	0
1b-32.out	-1272.401978	4.187998	0.000845	0.000106	0
1b-41.out	-1272.400449	5.14746	0.000167	2.09E-05	0
1b-38.out	-1272.400447	5.148715	0.000167	2.09E-05	0
1b-16.out	-1272.400305	5.237821	0.000143	1.80E-05	0
1b-24.out	-1272.399381	5.81764	5.38E-05	6.75E-06	0
1b-31.out	-1272.395902	8.000745	1.35E-06	1.69E-07	0
1b-35.out	-1272.394922	8.615704	4.76E-07	5.97E-08	0

Table S4. Coordinates (Ångstroms) for conformer **1a-1**

Atoms	X	Y	Z
C	4.41098	-1.45295	-0.66946
C	4.28471	-2.30891	0.58444
C	2.81339	-2.7684	0.69912
C	1.81294	-1.59882	0.6884
C	1.9987	-0.67543	-0.53337
C	3.48526	-0.23768	-0.73795
C	1.8332	-0.80872	1.9714
C	1.63571	0.52463	2.08053
C	1.3527	1.32908	0.87606
C	1.05293	0.55337	-0.40325
C	3.71043	0.56561	-2.0224
O	5.19485	-1.72392	-1.56674
C	5.26924	-3.47685	0.60617
C	1.32087	2.68866	0.94522
C	1.71169	1.19597	3.43261
C	-0.40832	0.14429	-0.38891
C	-2.64945	-0.31044	-1.29925
C	-1.25416	0.12955	-1.44239
C	-3.55254	-0.04065	-0.31575
C	-3.29727	0.86625	0.86277
C	-4.86329	-0.65873	-0.44431
C	-5.88946	-0.56364	0.42995
C	-7.15905	-1.25207	0.16384
O	-8.06201	-1.03798	1.15425
O	-7.42093	-1.94279	-0.81049
C	1.07696	3.65543	-0.1446
C	1.11542	5.11505	0.27544
O	0.86155	3.35521	-1.32046
C	-0.85584	0.47317	-2.8623
H	4.48806	-1.64334	1.43712
H	2.68818	-3.35859	1.6146
H	2.58592	-3.43882	-0.1395
H	0.81245	-2.0517	0.60754
H	1.69628	-1.22734	-1.43263
H	3.76436	0.38459	0.12525
H	2.01393	-1.37855	2.88238
H	1.21069	1.22106	-1.24426
H	3.18289	1.52154	-1.99332
H	4.77468	0.7698	-2.16147

H	3.36563	0.00817	-2.89953
H	5.16999	-4.04084	1.53856
H	6.30129	-3.12562	0.52438
H	5.08451	-4.16016	-0.22848
H	1.50872	3.15413	1.90742
H	1.86542	0.45528	4.22061
H	2.54034	1.91178	3.48206
H	0.79442	1.75059	3.65946
H	-0.79087	-0.19426	0.57055
H	-3.01506	-0.90987	-2.13441
H	-2.40183	1.47082	0.71576
H	-4.14766	1.53851	1.01759
H	-3.16841	0.29566	1.79045
H	-5.02851	-1.2684	-1.33116
H	-5.8239	0.00668	1.34935
H	-8.86886	-1.52132	0.90719
H	0.36662	5.30867	1.05192
H	2.09185	5.3622	0.70763
H	0.92411	5.7556	-0.58621
H	0.20661	0.69364	-2.96467
H	-1.09438	-0.36087	-3.53319
H	-1.41968	1.3401	-3.22571

Table S5. Coordinates (Ångstroms) for conformer **1a-2**

Atoms	X	Y	Z
C	4.02946	-1.78075	-0.89131
C	3.8174	-2.69596	0.30759
C	2.29661	-2.91936	0.46652
C	1.50216	-1.6066	0.59124
C	1.7879	-0.62982	-0.56876
C	3.31739	-0.42911	-0.8257
C	1.69564	-0.93827	1.92746
C	1.73166	0.39557	2.14626
C	1.56486	1.33166	1.0175
C	1.07594	0.7287	-0.29671
C	3.61852	0.42219	-2.06289
O	4.71662	-2.10767	-1.84726
C	4.59617	-4.00568	0.1994
C	1.80142	2.66194	1.18538
C	1.96045	0.93059	3.54129
C	-0.43171	0.57308	-0.23848
C	-2.76783	0.7492	-0.99191
C	-1.32182	0.93115	-1.19118
C	-3.45627	-0.29976	-0.46203
C	-2.83319	-1.59745	-0.01102
C	-4.8997	-0.15017	-0.35918
C	-5.76667	-1.03854	0.1752
C	-7.20453	-0.74293	0.21483
O	-7.90715	-1.75085	0.7915
O	-7.75066	0.26763	-0.20407
C	1.73047	3.74333	0.18211
C	2.00738	5.13529	0.72385
O	1.48382	3.58559	-1.01554
C	-0.95318	1.59704	-2.50115
H	4.16151	-2.13732	1.19121
H	2.11231	-3.55145	1.34318
H	1.92824	-3.47646	-0.4044
H	0.44011	-1.88988	0.52997
H	1.35654	-1.0476	-1.48747
H	3.73428	0.07079	0.06129
H	1.80272	-1.60354	2.78367
H	1.31985	1.4191	-1.09706
H	3.2612	1.4468	-1.93939
H	4.69499	0.45771	-2.24625

H	3.1469	-0.0024	-2.95518
H	4.44538	-4.61326	1.09683
H	5.66748	-3.82034	0.085
H	4.26594	-4.58709	-0.66697
H	2.09749	3.00463	2.17166
H	1.99638	0.11281	4.2646
H	2.90599	1.48034	3.61327
H	1.16443	1.61989	3.84395
H	-0.82471	0.15679	0.68584
H	-3.37167	1.57957	-1.36112
H	-1.81809	-1.70402	-0.39537
H	-2.78883	-1.66807	1.08242
H	-3.42537	-2.4487	-0.36237
H	-5.32102	0.77825	-0.74139
H	-5.449	-1.98553	0.59604
H	-8.84182	-1.48273	0.78085
H	1.29399	5.3846	1.51761
H	3.00724	5.17891	1.17054
H	1.93362	5.86894	-0.07965
H	-0.99775	0.87745	-3.32782
H	-1.67182	2.38958	-2.73831
H	0.04133	2.04262	-2.48202

Table S6. Coordinates (Ångstroms) for conformer **1a-3**

Atoms	X	Y	Z
C	4.39849	-1.46463	-0.67861
C	4.27872	-2.31099	0.58242
C	2.80725	-2.76638	0.71041
C	1.80935	-1.59465	0.69793
C	1.98869	-0.68073	-0.5319
C	3.47479	-0.24795	-0.75008
C	1.84045	-0.79515	1.97489
C	1.64672	0.53942	2.07552
C	1.3568	1.33558	0.8672
C	1.04674	0.55121	-0.40438
C	3.69275	0.54495	-2.04221
O	5.17599	-1.7438	-1.57891
C	5.26088	-3.48088	0.60634
C	1.32758	2.69569	0.92675
C	1.7338	1.22056	3.42202
C	-0.41537	0.14602	-0.37692
C	-2.6658	-0.30409	-1.26668
C	-1.27018	0.1308	-1.42309
C	-3.56009	-0.02724	-0.27731
C	-3.29197	0.88464	0.89436
C	-4.87487	-0.64277	-0.39293
C	-5.88653	-0.53728	0.49831
C	-7.19286	-1.18291	0.33491
O	-7.31927	-1.90686	-0.8065
O	-8.10666	-1.09257	1.14271
C	1.07753	3.655	-0.16826
C	1.12047	5.11751	0.24122
O	0.85404	3.34674	-1.34054
C	-0.88302	0.4689	-2.8474
H	4.48919	-1.63953	1.42873
H	2.68698	-3.34954	1.63103
H	2.57259	-3.44244	-0.1217
H	0.80733	-2.04586	0.62742
H	1.67878	-1.23857	-1.42495
H	3.76119	0.38033	0.10636
H	2.02635	-1.35867	2.88876
H	1.20044	1.21243	-1.25121
H	3.16786	1.50244	-2.01668
H	4.75649	0.74528	-2.19054

H	3.34018	-0.01827	-2.91254
H	5.1666	-4.03771	1.54354
H	6.29311	-3.13254	0.51512
H	5.06914	-4.16997	-0.22196
H	1.52269	3.16776	1.88427
H	1.89225	0.48546	4.21429
H	2.56389	1.93538	3.46002
H	0.8191	1.77831	3.65159
H	-0.79094	-0.18828	0.58679
H	-3.03964	-0.90615	-2.09638
H	-3.16104	0.31888	1.82471
H	-2.3936	1.48236	0.73808
H	-4.13675	1.56368	1.05038
H	-5.0406	-1.25117	-1.27912
H	-5.79317	0.03838	1.41207
H	-8.21656	-2.28002	-0.79809
H	2.1003	5.36662	0.66451
H	0.92363	5.75221	-0.6235
H	0.37757	5.31746	1.02174
H	0.17952	0.68383	-2.95993
H	-1.13177	-0.3656	-3.514
H	-1.4458	1.33774	-3.20789

Table S7. Coordinates (Ångstroms) for conformer **1a-4**

Atoms	X	Y	Z
C	4.04112	-1.75421	-0.89694
C	3.84456	-2.66739	0.30612
C	2.32697	-2.90587	0.47356
C	1.51959	-1.60092	0.59764
C	1.78935	-0.62539	-0.5672
C	3.31536	-0.40981	-0.83252
C	1.71279	-0.92585	1.9305
C	1.73605	0.40908	2.14441
C	1.5539	1.33933	1.01321
C	1.06475	0.72665	-0.29636
C	3.6015	0.44008	-2.07423
O	4.72693	-2.0773	-1.85517
C	4.63621	-3.96943	0.19833
C	1.77781	2.67256	1.17513
C	1.96607	0.95143	3.5364
C	-0.44096	0.55581	-0.23035
C	-2.78222	0.70411	-0.97466
C	-1.33886	0.89993	-1.18079
C	-3.45824	-0.35009	-0.4396
C	-2.82012	-1.6404	0.01106
C	-4.90409	-0.21359	-0.3315
C	-5.75239	-1.11545	0.21246
C	-7.20356	-0.92524	0.30178
O	-7.6461	0.24839	-0.21753
O	-7.9711	-1.73854	0.79689
C	1.69087	3.74953	0.1684
C	1.95974	5.14579	0.7031
O	1.4373	3.58532	-1.02695
C	-0.98279	1.56313	-2.49559
H	4.18741	-2.10222	1.18607
H	2.15361	-3.53662	1.35341
H	1.96	-3.46989	-0.39348
H	0.46023	-1.89534	0.54276
H	1.35775	-1.05082	-1.48226
H	3.73149	0.09759	0.05056
H	1.83098	-1.58691	2.78851
H	1.29759	1.41666	-1.10036
H	3.23475	1.46153	-1.95237
H	4.67657	0.48552	-2.26347

H	3.12935	0.00772	-2.96251
H	4.49607	-4.57556	1.09845
H	5.70499	-3.77352	0.07803
H	4.30771	-4.55701	-0.66453
H	2.07577	3.02171	2.15857
H	2.01377	0.13667	4.26244
H	2.90629	1.51102	3.60193
H	1.16451	1.63368	3.84043
H	-0.82556	0.13992	0.6977
H	-3.39538	1.5279	-1.34338
H	-1.80363	-1.73416	-0.37276
H	-2.77562	-1.71147	1.10446
H	-3.40132	-2.49871	-0.34151
H	-5.32611	0.71156	-0.71742
H	-5.4066	-2.05352	0.6314
H	-8.61063	0.25609	-0.09879
H	1.24936	5.39226	1.50044
H	2.96201	5.19936	1.14325
H	1.8748	5.87574	-0.10266
H	0.00718	2.01895	-2.483
H	-1.02351	0.83906	-3.31854
H	-1.71056	2.34708	-2.73348

Table S8. Coordinates (Ångstroms) for conformer **1a-5**

Atoms	X	Y	Z
C	4.02925	-1.78088	-0.89148
C	3.81744	-2.69598	0.30754
C	2.29666	-2.91931	0.46685
C	1.5023	-1.60649	0.59158
C	1.78786	-0.62987	-0.5686
C	3.31732	-0.42918	-0.82578
C	1.69605	-0.938	1.92767
C	1.73202	0.39587	2.14632
C	1.56494	1.33181	1.01749
C	1.07595	0.72869	-0.29662
C	3.61825	0.4221	-2.06303
O	4.71609	-2.10797	-1.84762
C	4.59609	-4.00577	0.19926
C	1.8014	2.66214	1.1852
C	1.96103	0.93106	3.54124
C	-0.4317	0.57311	-0.2383
C	-2.76779	0.74901	-0.99186
C	-1.32177	0.93085	-1.19114
C	-3.45626	-0.29978	-0.46168
C	-2.83315	-1.59726	-0.01009
C	-4.89971	-0.15021	-0.35907
C	-5.76675	-1.03845	0.17542
C	-7.20463	-0.74287	0.21469
O	-7.90733	-1.75063	0.79154
O	-7.75069	0.26761	-0.2045
C	1.73033	3.7434	0.18181
C	2.00748	5.1354	0.72337
O	1.4835	3.58555	-1.01578
C	-0.95308	1.59616	-2.5014
H	4.16179	-2.1373	1.19103
H	2.11255	-3.55127	1.34365
H	1.92809	-3.4765	-0.40391
H	0.44022	-1.88971	0.53055
H	1.35638	-1.04777	-1.48719
H	3.73434	0.07073	0.06114
H	1.80337	-1.60317	2.78394
H	1.31981	1.41899	-1.09707
H	3.2611	1.44676	-1.93941
H	4.69467	0.45745	-2.24669

H	3.14632	-0.00239	-2.9552
H	4.4454	-4.61332	1.09673
H	5.66741	-3.82053	0.0847
H	4.26569	-4.58717	-0.66704
H	2.09753	3.00496	2.17142
H	1.99728	0.11337	4.26463
H	2.90648	1.48101	3.61298
H	1.16493	1.62022	3.844
H	-0.82473	0.15716	0.68616
H	-3.37159	1.57927	-1.36136
H	-1.81803	-1.70396	-0.39439
H	-2.78878	-1.66736	1.08338
H	-3.42526	-2.4487	-0.36107
H	-5.321	0.77808	-0.74162
H	-5.44914	-1.98531	0.59661
H	-8.842	-1.48249	0.78068
H	3.00734	5.17889	1.17009
H	1.93382	5.86897	-0.0802
H	1.29412	5.38488	1.5171
H	-0.99703	0.87604	-3.32765
H	-1.67203	2.3882	-2.73925
H	0.04122	2.04221	-2.48225

Table S9. Coordinates (Ångstroms) for conformer **1a-6**

Atoms	X	Y	Z
C	4.41112	-1.45282	-0.66934
C	4.28491	-2.30867	0.58466
C	2.81361	-2.7682	0.69939
C	1.81311	-1.59867	0.68856
C	1.99883	-0.67539	-0.5333
C	3.48537	-0.23757	-0.73789
C	1.83338	-0.80842	1.97147
C	1.63584	0.52493	2.08046
C	1.35266	1.32925	0.87594
C	1.05299	0.55335	-0.40332
C	3.71056	0.56564	-2.02238
O	5.19494	-1.72387	-1.56664
C	5.26949	-3.47656	0.60647
C	1.32067	2.68883	0.94497
C	1.71192	1.19643	3.43246
C	-0.40824	0.14425	-0.38895
C	-2.64937	-0.31078	-1.29922
C	-1.2541	0.12923	-1.44242
C	-3.5525	-0.04081	-0.31581
C	-3.29738	0.86643	0.86249
C	-4.86322	-0.65899	-0.44426
C	-5.8894	-0.56371	0.42995
C	-7.15897	-1.25225	0.164
O	-8.06195	-1.0379	1.15433
O	-7.42076	-1.94335	-0.81008
C	1.07642	3.6555	-0.14489
C	1.11392	5.11512	0.27523
O	0.8614	3.35521	-1.32079
C	-0.85575	0.47218	-2.86249
H	4.48824	-1.64301	1.43727
H	2.68842	-3.3583	1.61493
H	2.58615	-3.43872	-0.13916
H	0.81264	-2.05159	0.60775
H	1.69646	-1.2274	-1.43252
H	3.76444	0.38473	0.12529
H	2.01416	-1.37813	2.88252
H	1.21076	1.22097	-1.24439
H	3.18298	1.52156	-1.99338
H	4.77481	0.76987	-2.16142

H	3.36581	0.00813	-2.89949
H	5.17036	-4.04041	1.53896
H	6.3015	-3.12528	0.52451
H	5.0847	-4.16002	-0.22805
H	1.50848	3.15441	1.90712
H	1.86598	0.45587	4.22051
H	2.5404	1.91244	3.48166
H	0.79456	1.75088	3.65941
H	-0.7908	-0.19409	0.57058
H	-3.01497	-0.91036	-2.13428
H	-2.4018	1.47079	0.71553
H	-4.14773	1.53886	1.01682
H	-3.16891	0.29613	1.7904
H	-5.02838	-1.2689	-1.33096
H	-5.82389	0.00688	1.34918
H	-8.86876	-1.5214	0.90743
H	0.36421	5.30843	1.05092
H	2.08977	5.36267	0.70845
H	0.92319	5.75559	-0.58662
H	0.20613	0.69559	-2.96432
H	-1.09135	-0.36347	-3.53243
H	-1.42185	1.33695	-3.22749

Table S10. Coordinates (Ångstroms) for conformer **1a-7**

Atoms	X	Y	Z
C	4.04064	-1.75476	-0.89701
C	3.84418	-2.66773	0.30622
C	2.32656	-2.90596	0.47391
C	1.5194	-1.60087	0.59791
C	1.78919	-0.62551	-0.56708
C	3.31523	-0.4102	-0.83257
C	1.71281	-0.92566	1.93066
C	1.73627	0.40928	2.14442
C	1.55419	1.33942	1.01312
C	1.06481	0.72667	-0.29632
C	3.60137	0.43956	-2.07437
O	4.72609	-2.07817	-1.8554
C	4.6356	-3.96991	0.19848
C	1.77836	2.67263	1.17487
C	1.96646	0.95178	3.53633
C	-0.44091	0.55603	-0.23013
C	-2.78224	0.70468	-0.97405
C	-1.33888	0.90035	-1.18042
C	-3.45829	-0.34977	-0.43956
C	-2.8202	-1.64042	0.0102
C	-4.9041	-0.21322	-0.33115
C	-5.75244	-1.11545	0.21213
C	-7.20359	-0.92518	0.30173
O	-7.64605	0.24896	-0.21647
O	-7.97117	-1.73886	0.79617
C	1.69157	3.7495	0.16801
C	1.96055	5.14579	0.70262
O	1.43807	3.5852	-1.02733
C	-0.98296	1.56363	-2.49523
H	4.18723	-2.10249	1.18604
H	2.15324	-3.53656	1.35388
H	1.95939	-3.47006	-0.39299
H	0.45998	-1.89513	0.54316
H	1.35745	-1.05099	-1.48205
H	3.73153	0.09717	0.05044
H	1.83097	-1.58664	2.78874
H	1.29766	1.41653	-1.10044
H	3.23484	1.46109	-1.95253
H	4.67642	0.48478	-2.26376

H	3.12902	0.00724	-2.96257
H	4.49544	-4.57594	1.09867
H	5.70441	-3.7742	0.07808
H	4.30693	-4.55752	-0.66429
H	2.07639	3.02185	2.15826
H	2.01417	0.13711	4.26247
H	2.90673	1.51131	3.60172
H	1.16497	1.63412	3.84035
H	-0.82547	0.14008	0.69792
H	-3.39533	1.52884	-1.3421
H	-1.80367	-1.7339	-0.37358
H	-2.77582	-1.71225	1.10355
H	-3.40135	-2.49849	-0.34305
H	-5.32607	0.71229	-0.71625
H	-5.40669	-2.0539	0.63025
H	-8.61058	0.25665	-0.09764
H	2.96271	5.1992	1.14306
H	1.87598	5.87565	-0.10325
H	1.24999	5.39246	1.49974
H	-1.02376	0.83957	-3.31819
H	-1.7108	2.34755	-2.733
H	0.00699	2.01949	-2.48271

Table S11. Coordinates (Ångstroms) for conformer **1a-8**

Atoms	X	Y	Z
C	4.39843	-1.46473	-0.67857
C	4.27863	-2.31104	0.5825
C	2.80712	-2.76639	0.71039
C	1.80927	-1.59463	0.69787
C	1.98869	-0.68069	-0.53194
C	3.47484	-0.24797	-0.75005
C	1.84033	-0.79515	1.97485
C	1.64668	0.53943	2.0755
C	1.35683	1.33564	0.86718
C	1.04678	0.55127	-0.40444
C	3.69294	0.5449	-2.04217
O	5.17581	-1.74404	-1.57893
C	5.26076	-3.48095	0.60649
C	1.32772	2.69574	0.92673
C	1.73373	1.22054	3.42201
C	-0.41535	0.14618	-0.377
C	-2.66582	-0.30385	-1.26675
C	-1.27019	0.13096	-1.42315
C	-3.56006	-0.02718	-0.27728
C	-3.29192	0.88449	0.89455
C	-4.87486	-0.64266	-0.39297
C	-5.88645	-0.5374	0.49838
C	-7.1928	-1.18295	0.33491
O	-7.31927	-1.90667	-0.80663
O	-8.10656	-1.09274	1.14278
C	1.07763	3.65507	-0.16827
C	1.1204	5.11755	0.2413
O	0.85417	3.34684	-1.34055
C	-0.88299	0.46872	-2.84753
H	4.48904	-1.63956	1.4288
H	2.68677	-3.34956	1.63099
H	2.57249	-3.44243	-0.12175
H	0.80724	-2.04581	0.6273
H	1.6788	-1.23849	-1.42501
H	3.76123	0.38027	0.10642
H	2.02614	-1.35869	2.88872
H	1.20054	1.21249	-1.25126
H	3.16806	1.50239	-2.01672
H	4.7567	0.74523	-2.19037

H	3.34048	-0.01835	-2.91252
H	5.16651	-4.03767	1.54375
H	6.29298	-3.13262	0.51519
H	5.06896	-4.17012	-0.22173
H	1.52287	3.1678	1.88424
H	1.89209	0.48541	4.21428
H	2.56388	1.9353	3.46006
H	0.81907	1.77836	3.65154
H	-0.79091	-0.18807	0.58672
H	-3.03977	-0.90564	-2.09659
H	-2.39356	1.48224	0.73836
H	-4.13672	1.56349	1.05067
H	-3.16101	0.3186	1.82482
H	-5.04068	-1.25081	-1.27931
H	-5.79301	0.03802	1.41229
H	-8.21658	-2.27977	-0.79829
H	2.09997	5.36669	0.66516
H	0.92396	5.75231	-0.62348
H	0.37705	5.31745	1.02141
H	-1.12912	-0.36713	-3.51344
H	-1.44779	1.3357	-3.20927
H	0.17907	0.6863	-2.95953

Table S12. Coordinates (Ångstroms) for conformer **1a-17**

Atoms	X	Y	Z
C	4.2037	-1.70282	-0.93963
C	4.20958	-2.51858	0.34693
C	2.7423	-2.83757	0.71299
C	1.8601	-1.58129	0.82518
C	1.92415	-0.7009	-0.4402
C	3.38904	-0.4088	-0.90058
C	2.15692	-0.77536	2.06347
C	2.1043	0.57242	2.16088
C	1.71335	1.38085	0.98994
C	1.13086	0.6185	-0.19648
C	3.46776	0.34179	-2.23342
O	4.80495	-2.06132	-1.94097
C	5.07714	-3.77232	0.24972
C	1.83715	2.73645	1.01251
C	2.45644	1.25549	3.46235
C	-0.33315	0.34357	0.06711
C	-2.67888	0.06137	-0.2806
C	-1.36687	0.43706	-0.80931
C	-3.89754	-0.15157	-0.86134
C	-4.22988	-0.1116	-2.33419
C	-4.9848	-0.46345	0.05285
C	-6.28109	-0.6773	-0.27068
C	-7.26682	-0.97401	0.77423
O	-8.50953	-1.152	0.25592
O	-7.05172	-1.06214	1.97532
C	1.5299	3.70386	-0.0605
C	1.77725	5.15833	0.30121
O	1.1129	3.40884	-1.18264
C	-1.18683	0.92173	-2.2309
H	4.60772	-1.85823	1.13235
H	2.71715	-3.39604	1.65609
H	2.32205	-3.49983	-0.05465
H	0.82473	-1.94372	0.92083
H	1.42645	-1.23518	-1.25952
H	3.86173	0.20323	-0.11806
H	2.42573	-1.34565	2.95212
H	1.21459	1.24726	-1.07658
H	3.03966	1.34342	-2.1539
H	4.50791	0.44177	-2.55218

H	2.93326	-0.19976	-3.02076
H	5.08003	-4.30782	1.2039
H	6.10946	-3.52004	-0.00661
H	4.70002	-4.45015	-0.52228
H	2.22414	3.19672	1.9159
H	2.65911	0.5166	4.24087
H	3.34837	1.88433	3.35983
H	1.64446	1.90403	3.8095
H	-0.56774	0.01234	1.07758
H	-2.66505	-0.0789	0.79965
H	-3.35234	-0.05293	-2.9709
H	-4.78206	-1.01491	-2.61568
H	-4.87935	0.74069	-2.56866
H	-4.73203	-0.52087	1.11025
H	-6.64522	-0.63657	-1.29061
H	-9.09968	-1.3427	1.00481
H	1.52492	5.80037	-0.54343
H	1.17608	5.44214	1.17251
H	2.82711	5.30961	0.57718
H	-0.25204	1.47058	-2.34469
H	-1.1791	0.09186	-2.94669
H	-1.99735	1.59626	-2.51835

Table S13. Coordinates (Ångstroms) for conformer **1a-37**

Atoms	X	Y	Z
C	4.02959	-1.78061	-0.8913
C	3.81751	-2.69591	0.30752
C	2.29673	-2.91932	0.46653
C	1.50227	-1.60657	0.5913
C	1.78794	-0.62981	-0.56873
C	3.31739	-0.42902	-0.8257
C	1.69578	-0.93823	1.9275
C	1.73168	0.39561	2.14629
C	1.56475	1.33168	1.01753
C	1.07591	0.72867	-0.29669
C	3.61846	0.42231	-2.06289
O	4.71691	-2.1074	-1.84719
C	4.59625	-4.00564	0.19921
C	1.80122	2.66198	1.1854
C	1.96042	0.93068	3.54131
C	-0.43173	0.57298	-0.2385
C	-2.76781	0.74889	-0.99213
C	-1.32181	0.93084	-1.1913
C	-3.45629	-0.29988	-0.46191
C	-2.83322	-1.59737	-0.0103
C	-4.89974	-0.15029	-0.35929
C	-5.76675	-1.03844	0.1754
C	-7.20462	-0.74284	0.21473
O	-7.90729	-1.75054	0.79175
O	-7.75072	0.26754	-0.20465
C	1.7303	3.74335	0.18211
C	2.00761	5.13526	0.72379
O	1.48341	3.58565	-1.01549
C	-0.95309	1.59644	-2.50141
H	4.16169	-2.13734	1.19115
H	2.11248	-3.55141	1.3432
H	1.92832	-3.47641	-0.40437
H	0.44023	-1.88987	0.53009
H	1.35658	-1.04764	-1.48741
H	3.73429	0.07091	0.06128
H	1.80298	-1.60349	2.7837
H	1.3198	1.41907	-1.09705
H	3.26129	1.44696	-1.93929
H	4.6949	0.45767	-2.24643

H	3.14663	-0.00217	-2.95513
H	4.4455	-4.61326	1.09662
H	5.66755	-3.82033	0.08474
H	4.26594	-4.58701	-0.66717
H	2.09727	3.0047	2.17168
H	1.99648	0.11291	4.26463
H	2.90587	1.48058	3.61329
H	1.16427	1.61984	3.84397
H	-0.82476	0.15686	0.68589
H	-3.37165	1.57908	-1.36173
H	-1.81811	-1.70411	-0.39458
H	-2.78889	-1.66751	1.08317
H	-3.42537	-2.44878	-0.36131
H	-5.32104	0.77794	-0.74196
H	-5.44911	-1.98523	0.59671
H	-8.84195	-1.48242	0.7809
H	3.00737	5.17855	1.17075
H	1.93427	5.86888	-0.07976
H	1.2941	5.38486	1.51736
H	-1.67188	2.38871	-2.73896
H	0.04131	2.04227	-2.48223
H	-0.99728	0.87657	-3.32786

Table S14. Coordinates (Ångstroms) for conformer **1a-39**

Atoms	X	Y	Z
C	4.41125	-1.45259	-0.66946
C	4.2852	-2.30852	0.58451
C	2.81394	-2.76816	0.69934
C	1.81331	-1.59872	0.68853
C	1.9989	-0.67538	-0.5333
C	3.48543	-0.23741	-0.73794
C	1.83346	-0.80853	1.97147
C	1.63575	0.5248	2.08052
C	1.35256	1.32913	0.87603
C	1.05301	0.55329	-0.40327
C	3.71046	0.56585	-2.02244
O	5.19498	-1.72361	-1.56685
C	5.26983	-3.47637	0.60616
C	1.32032	2.68871	0.94517
C	1.71167	1.19622	3.43257
C	-0.40821	0.14406	-0.38895
C	-2.64936	-0.31072	-1.2992
C	-1.2541	0.12938	-1.44239
C	-3.55244	-0.04098	-0.31569
C	-3.2972	0.8659	0.86286
C	-4.86319	-0.65907	-0.44425
C	-5.88938	-0.56395	0.42997
C	-7.15897	-1.25235	0.16382
O	-8.06198	-1.03817	1.15417
O	-7.4208	-1.94314	-0.81047
C	1.07616	3.65538	-0.14467
C	1.11344	5.11503	0.27541
O	0.86146	3.35509	-1.32065
C	-0.85591	0.47303	-2.86232
H	4.48856	-1.64291	1.43715
H	2.68886	-3.35824	1.61489
H	2.58648	-3.43871	-0.13919
H	0.8129	-2.05179	0.60772
H	1.69656	-1.2274	-1.43253
H	3.76443	0.38494	0.12523
H	2.0143	-1.37826	2.88248
H	1.21075	1.22096	-1.24431
H	3.18305	1.52184	-1.99324
H	4.77472	0.76989	-2.16169

H	3.36542	0.00847	-2.89952
H	5.17067	-4.04039	1.53854
H	6.30184	-3.12505	0.52434
H	5.08512	-4.15968	-0.2285
H	1.50789	3.15424	1.90739
H	1.8658	0.45562	4.22057
H	2.54004	1.91235	3.48187
H	0.79421	1.75049	3.65953
H	-0.79071	-0.19458	0.57049
H	-3.01497	-0.91015	-2.13438
H	-2.40167	1.47036	0.71596
H	-4.14754	1.53824	1.01759
H	-3.1685	0.29527	1.79055
H	-5.02839	-1.26874	-1.3311
H	-5.82384	0.00641	1.34935
H	-8.86882	-1.52152	0.90708
H	0.9216	5.75538	-0.58628
H	0.36456	5.30812	1.05193
H	2.08973	5.36297	0.70745
H	-1.42082	1.33906	-3.2262
H	0.20627	0.69487	-2.96449
H	-1.09317	-0.36159	-3.53293

Table S15. Coordinates (Ångstroms) for conformer **1b-1**

Atoms	X	Y	Z
C	-2.81361	-2.76811	0.69969
C	-1.81313	-1.59856	0.68882
C	-4.2849	-2.30863	0.58472
C	-1.83354	-0.80818	1.97165
C	-1.99874	-0.67542	-0.53317
C	-1.05293	0.55336	-0.40321
H	-1.69626	-1.22754	-1.43228
C	-3.48528	-0.23767	-0.738
H	-4.48834	-1.64288	1.43724
C	-4.41103	-1.45291	-0.66937
C	-5.26946	-3.47654	0.60657
C	-3.7103	0.56537	-2.02262
H	-3.76446	0.38476	0.12506
H	-1.21058	1.22091	-1.24436
C	-1.35271	1.32935	0.87592
C	0.40831	0.1442	-0.38876
H	-0.81264	-2.05146	0.60816
H	-2.68852	-3.3581	1.61531
H	-2.58604	-3.43873	-0.13876
C	-1.636	0.52519	2.08051
H	-4.77454	0.76952	-2.16187
H	-3.18278	1.52132	-1.99364
H	-3.36536	0.00778	-2.89961
H	-6.30149	-3.12531	0.52461
H	-5.08465	-4.16001	-0.22794
H	-5.17031	-4.04037	1.53907
H	-2.01443	-1.37779	2.88273
C	-1.71225	1.19684	3.43242
C	-1.32071	2.68894	0.94483
O	-5.19477	-1.72407	-1.5667
H	-1.86619	0.45633	4.22055
H	-2.54089	1.91268	3.48151
H	-0.79502	1.75149	3.65937
C	1.25404	0.1288	-1.44232
C	2.64933	-0.31116	-1.29909
C	0.85556	0.47163	-2.86238
H	0.79094	-0.19378	0.57087
H	1.09371	-0.36291	-3.53278
H	-0.20685	0.69234	-2.96467

H	1.41958	1.33815	-3.22646
C	3.55254	-0.04085	-0.31584
H	3.01484	-0.91108	-2.13394
C	4.86324	-0.6591	-0.44415
C	3.29749	0.86679	0.86217
C	5.88949	-0.56351	0.42995
H	4.14785	1.53926	1.01626
H	2.40192	1.47113	0.71505
H	3.16903	0.29678	1.79027
H	5.02833	-1.26932	-1.33064
C	7.15902	-1.25217	0.16413
H	5.82406	0.00741	1.34898
O	7.42075	-1.94353	-0.80978
O	8.06209	-1.03748	1.15431
H	8.86889	-1.52103	0.90748
H	-1.50868	3.15461	1.9069
C	-1.07639	3.65546	-0.14513
O	-0.86077	3.35495	-1.32088
C	-1.11464	5.11519	0.27458
H	-2.09103	5.36267	0.70664
H	-0.36579	5.30891	1.05099
H	-0.92315	5.75546	-0.58725

Table S16. Coordinates (Ångstroms) for conformer **1b-2**

Atoms	X	Y	Z
C	-2.29642	-2.91942	0.46654
C	-1.50205	-1.60661	0.59127
C	-3.81723	-2.6961	0.30758
C	-1.69559	-0.93829	1.92748
C	-1.78782	-0.62987	-0.56875
C	-1.07593	0.72869	-0.2967
H	-1.35644	-1.04765	-1.48745
C	-3.31731	-0.42923	-0.82571
H	-4.16139	-2.13749	1.19119
C	-4.02931	-1.78091	-0.89131
C	-4.5959	-4.00588	0.19937
C	-3.61847	0.42206	-2.0629
H	-3.73424	0.07066	0.06127
H	-1.31987	1.41905	-1.09707
C	-1.56491	1.33163	1.01749
C	0.43172	0.57313	-0.23845
H	-0.43999	-1.88983	0.53004
H	-2.11211	-3.5515	1.34321
H	-1.92801	-3.4765	-0.40437
C	-1.73166	0.39556	2.14626
H	-4.69492	0.45744	-2.24634
H	-3.26127	1.44671	-1.93935
H	-3.14673	-0.00246	-2.95517
H	-5.66722	-3.82063	0.08495
H	-4.26561	-4.58727	-0.66699
H	-4.44509	-4.61345	1.09681
H	-1.80265	-1.60355	2.78369
C	-1.96047	0.93059	3.54128
C	-1.80161	2.6619	1.18534
O	-4.71643	-2.10788	-1.84729
H	-1.99633	0.11283	4.26462
H	-2.90603	1.4803	3.61328
H	-1.16446	1.61993	3.84391
C	1.32184	0.9313	-1.19109
C	2.76784	0.74938	-0.99181
C	0.95321	1.59724	-2.50105
H	0.82472	0.1568	0.68586
H	1.67199	2.38962	-2.7383
H	0.99753	0.87761	-3.32769

H	-0.04121	2.04302	-2.48183
C	3.4563	-0.29962	-0.46202
H	3.37166	1.57981	-1.36091
C	4.89973	-0.15001	-0.35912
C	2.83325	-1.59737	-0.01116
C	5.76671	-1.03843	0.17516
H	3.42538	-2.44857	-0.36273
H	2.789	-1.66817	1.08227
H	1.81811	-1.70388	-0.39543
H	5.32104	0.77847	-0.74121
C	7.20456	-0.74281	0.21484
H	5.44905	-1.98549	0.59586
O	7.75067	0.26785	-0.20386
O	7.90719	-1.75082	0.79134
H	8.84185	-1.48267	0.78075
H	-2.0977	3.00458	2.17162
C	-1.73084	3.74328	0.18205
O	-1.48419	3.58559	-1.0156
C	-2.00802	5.13518	0.7238
H	-3.00778	5.17852	1.17076
H	-1.29451	5.38468	1.5174
H	-1.93463	5.86885	-0.07972

Table S17. Coordinates (Ångstroms) for conformer **1b-3**

Atoms	X	Y	Z
C	-2.80723	-2.76641	0.71043
C	-1.80932	-1.59469	0.69795
C	-4.2787	-2.311	0.58241
C	-1.84041	-0.7952	1.97492
C	-1.98863	-0.68076	-0.5319
C	-1.04672	0.55121	-0.40436
H	-1.67868	-1.2386	-1.42493
C	-3.47474	-0.24803	-0.75016
H	-4.48916	-1.63949	1.42867
C	-4.3984	-1.46473	-0.67868
C	-5.26088	-3.48088	0.60637
C	-3.69267	0.54481	-2.04233
H	-3.76118	0.38029	0.10625
H	-1.20043	1.21244	-1.25118
C	-1.35681	1.33555	0.86723
C	0.41541	0.14606	-0.37693
H	-0.80731	-2.04591	0.62743
H	-2.68698	-3.34955	1.63107
H	-2.57257	-3.44249	-0.12166
C	-1.64671	0.53937	2.07556
H	-4.7564	0.74516	-2.19068
H	-3.16776	1.5023	-2.01683
H	-3.34009	-0.01845	-2.91262
H	-6.2931	-3.13253	0.51509
H	-5.06912	-4.17003	-0.22186
H	-5.16664	-4.03763	1.54362
H	-2.02627	-1.35873	2.88878
C	-1.73383	1.22052	3.42205
C	-1.32775	2.69565	0.92678
O	-5.17577	-1.74402	-1.57906
H	-1.89215	0.48539	4.21434
H	-2.56403	1.93521	3.46007
H	-0.81921	1.77841	3.65158
C	1.27023	0.13094	-1.42309
C	2.66584	-0.30398	-1.26669
C	0.8831	0.46917	-2.84738
H	0.79099	-0.18828	0.58676
H	1.1317	-0.36536	-3.51401
H	-0.17941	0.68427	-2.95987

H	1.446	1.33793	-3.20784
C	3.56012	-0.02715	-0.2773
H	3.03968	-0.90603	-2.09638
C	4.87489	-0.64272	-0.3929
C	3.29202	0.88471	0.89438
C	5.88657	-0.53723	0.49832
H	3.16124	0.31895	1.82474
H	4.13675	1.56383	1.05031
H	2.39358	1.48234	0.73817
H	5.04061	-1.25114	-1.27908
C	7.19289	-1.18287	0.33491
H	5.79324	0.03846	1.41206
O	8.10675	-1.09241	1.14263
O	7.3192	-1.90697	-0.80642
H	8.2165	-2.2801	-0.79805
H	-1.523	3.16772	1.88427
C	-1.07772	3.65498	-0.16824
O	-0.85382	3.34673	-1.34043
C	-1.12116	5.11748	0.24121
H	-0.3787	5.31756	1.02212
H	-0.92398	5.75225	-0.62338
H	-2.10125	5.36639	0.66401

Table S18. Coordinates (Ångstroms) for conformer **1b-4**

Atoms	X	Y	Z
C	-2.32671	-2.90599	0.47359
C	-1.51941	-1.60099	0.59766
C	-3.84431	-2.66761	0.30611
C	-1.71267	-0.92591	1.93051
C	-1.78922	-0.62549	-0.5672
C	-1.06472	0.72662	-0.29635
H	-1.35755	-1.0509	-1.48224
C	-3.31524	-0.41003	-0.83257
H	-4.18721	-2.10244	1.18604
C	-4.04089	-1.75448	-0.89698
C	-4.63587	-3.96971	0.19834
C	-3.6014	0.4398	-2.07433
H	-3.73143	0.09735	0.05048
H	-1.29758	1.41658	-1.10038
C	-1.554	1.33926	1.01319
C	0.44099	0.55587	-0.23026
H	-0.46003	-1.89535	0.5428
H	-2.15332	-3.53672	1.35345
H	-1.95968	-3.47001	-0.39343
C	-1.73604	0.40901	2.14441
H	-4.67647	0.4851	-2.26363
H	-3.23478	1.4613	-1.95246
H	-3.12915	0.00748	-2.96257
H	-5.70466	-3.77388	0.07801
H	-4.3073	-4.55731	-0.66448
H	-4.49571	-4.5758	1.09849
H	-1.83079	-1.58697	2.78853
C	-1.96609	0.95137	3.53639
C	-1.77817	2.67246	1.17505
O	-4.72665	-2.07766	-1.85522
H	-2.01366	0.13663	4.26246
H	-2.90639	1.51083	3.60194
H	-1.16461	1.63373	3.84038
C	1.33894	0.90042	-1.18049
C	2.78229	0.70457	-0.97432
C	0.9829	1.56415	-2.49505
H	0.82553	0.13966	0.69767
H	1.71106	2.34773	-2.73294
H	1.02299	0.84025	-3.31818

H	-0.0068	2.02053	-2.48205
C	3.4583	-0.34985	-0.43968
H	3.39545	1.52855	-1.34262
C	4.90413	-0.21336	-0.33141
C	2.82017	-1.64038	0.01034
C	5.75242	-1.11545	0.2122
H	3.4014	-2.49852	-0.34262
H	2.77563	-1.71196	1.1037
H	1.80371	-1.73397	-0.37357
H	5.32616	0.71198	-0.71686
C	7.20357	-0.92524	0.30172
H	5.40661	-2.05371	0.63068
O	7.9711	-1.73875	0.7965
O	7.64612	0.24868	-0.21695
H	8.61064	0.25635	-0.09813
H	-2.07617	3.02158	2.1585
C	-1.69147	3.74943	0.16831
O	-1.43809	3.58528	-1.02708
C	-1.9604	5.14565	0.70311
H	-1.25005	5.3921	1.50049
H	-1.87557	5.87566	-0.1026
H	-2.96267	5.19906	1.14331

Table S19. Coordinates (Ångstroms) for conformer **1b-5**

Atoms	X	Y	Z
C	-2.2966	-2.91933	0.46681
C	-1.50225	-1.60651	0.59154
C	-3.81738	-2.69602	0.30754
C	-1.69597	-0.93805	1.92765
C	-1.78784	-0.62987	-0.56862
C	-1.07595	0.72869	-0.29663
H	-1.35637	-1.04775	-1.48722
C	-3.3173	-0.42921	-0.82577
H	-4.16172	-2.13736	1.19105
C	-4.02923	-1.78091	-0.89146
C	-4.59602	-4.00582	0.19927
C	-3.61826	0.42208	-2.063
H	-3.73432	0.0707	0.06116
H	-1.31983	1.419	-1.09707
C	-1.56495	1.33178	1.01749
C	0.4317	0.57313	-0.23832
H	-0.44017	-1.88972	0.53048
H	-2.11246	-3.55131	1.34358
H	-1.92803	-3.4765	-0.40398
C	-1.73197	0.39582	2.14631
H	-4.69469	0.45743	-2.24664
H	-3.26112	1.44674	-1.93938
H	-3.14635	-0.0024	-2.95519
H	-5.66734	-3.82059	0.08471
H	-4.26562	-4.58721	-0.66705
H	-4.44531	-4.61337	1.09673
H	-1.80326	-1.60323	2.7839
C	-1.96096	0.93098	3.54125
C	-1.80146	2.6621	1.18522
O	-4.71609	-2.10799	-1.84759
H	-1.99713	0.11328	4.26463
H	-2.90643	1.48088	3.61303
H	-1.16487	1.62018	3.844
C	1.32178	0.93093	-1.19113
C	2.7678	0.74908	-0.99184
C	0.9531	1.59633	-2.50135
H	0.82472	0.15712	0.68612
H	-0.04122	2.04233	-2.4822
H	1.67202	2.38843	-2.7391

H	0.99714	0.87628	-3.32765
C	3.45627	-0.29973	-0.46172
H	3.3716	1.57936	-1.36129
C	4.89972	-0.15017	-0.35907
C	2.83316	-1.59725	-0.01022
C	5.76675	-1.03842	0.1754
H	2.78879	-1.66744	1.08324
H	1.81805	-1.70393	-0.39454
H	3.42528	-2.44866	-0.36126
H	5.32101	0.77814	-0.74157
C	7.20462	-0.74284	0.21471
H	5.44913	-1.98531	0.59652
O	7.75068	0.26766	-0.20442
O	7.90732	-1.75064	0.7915
H	8.84198	-1.48251	0.78066
H	-2.09759	3.0049	2.17145
C	-1.73046	3.74338	0.18184
O	-1.48367	3.58555	-1.01576
C	-2.00763	5.13536	0.72342
H	-1.934	5.86894	-0.08014
H	-3.00748	5.17883	1.17016
H	-1.29426	5.38485	1.51715

Table S20. Coordinates (Ångstroms) for conformer **1b-6**

Atoms	X	Y	Z
C	-2.81358	-2.76821	0.69939
C	-1.81309	-1.59867	0.68856
C	-4.28488	-2.3087	0.58465
C	-1.83337	-0.80843	1.97148
C	-1.99881	-0.67539	-0.5333
C	-1.05298	0.55336	-0.40331
H	-1.69643	-1.2274	-1.43251
C	-3.48536	-0.23759	-0.7379
H	-4.48822	-1.64304	1.43726
C	-4.4111	-1.45285	-0.66935
C	-5.26945	-3.4766	0.60647
C	-3.71054	0.56561	-2.0224
H	-3.76443	0.38472	0.12528
H	-1.21076	1.22097	-1.24439
C	-1.35267	1.32925	0.87594
C	0.40825	0.14427	-0.38894
H	-0.81261	-2.05159	0.60776
H	-2.68838	-3.35831	1.61493
H	-2.58611	-3.43873	-0.13916
C	-1.63584	0.52493	2.08046
H	-4.7748	0.76984	-2.16143
H	-3.18297	1.52153	-1.99339
H	-3.36578	0.0081	-2.89949
H	-6.30147	-3.12533	0.52451
H	-5.08466	-4.16005	-0.22806
H	-5.17031	-4.04045	1.53895
H	-2.01414	-1.37814	2.88252
C	-1.71193	1.19642	3.43247
C	-1.3207	2.68883	0.94498
O	-5.19491	-1.7239	-1.56664
H	-1.86598	0.45586	4.22051
H	-2.54043	1.91242	3.48167
H	-0.79459	1.75089	3.65942
C	1.25411	0.12924	-1.44242
C	2.64938	-0.31075	-1.29922
C	0.85574	0.47217	-2.86248
H	0.79082	-0.19405	0.57059
H	1.09142	-0.36345	-3.53243
H	-0.20616	0.69549	-2.96433

H	1.42177	1.33699	-3.22747
C	3.55251	-0.04079	-0.3158
H	3.01499	-0.91031	-2.13429
C	4.86323	-0.65896	-0.44426
C	3.29737	0.86642	0.86252
C	5.8894	-0.56371	0.42997
H	4.14773	1.53882	1.0169
H	2.40181	1.4708	0.71555
H	3.16885	0.29608	1.79041
H	5.02841	-1.26883	-1.33097
C	7.15898	-1.25223	0.164
H	5.82388	0.00685	1.34922
O	7.42079	-1.94327	-0.81012
O	8.06196	-1.03788	1.15433
H	8.86878	-1.52134	0.90741
H	-1.50854	3.15441	1.90712
C	-1.07648	3.6555	-0.14489
O	-0.8614	3.3552	-1.32078
C	-1.11409	5.11512	0.2752
H	-2.09002	5.36263	0.7083
H	-0.36449	5.30849	1.05098
H	-0.92331	5.75559	-0.58664

Table S21. Coordinates (Ångstroms) for conformer **1b-7**

Atoms	X	Y	Z
C	-2.32664	-2.90592	0.47401
C	-1.51947	-1.60083	0.598
C	-3.84425	-2.66768	0.3062
C	-1.71294	-0.92558	1.93073
C	-1.7892	-0.62551	-0.56703
C	-1.06481	0.72667	-0.29629
H	-1.35743	-1.05102	-1.48197
C	-3.31522	-0.41016	-0.83258
H	-4.18735	-2.10242	1.18598
C	-4.04063	-1.75473	-0.89706
C	-4.63567	-3.96985	0.19843
C	-3.60128	0.4396	-2.07439
H	-3.73155	0.09721	0.05041
H	-1.29765	1.41652	-1.10042
C	-1.55418	1.33947	1.01314
C	0.44092	0.55604	-0.23009
H	-0.46005	-1.8951	0.54331
H	-2.1534	-3.53648	1.35402
H	-1.95942	-3.47007	-0.39284
C	-1.73637	0.40937	2.14445
H	-4.67633	0.48486	-2.26382
H	-3.23471	1.46112	-1.95254
H	-3.12892	0.00727	-2.96258
H	-5.70447	-3.77414	0.07796
H	-4.30695	-4.55748	-0.66431
H	-4.49558	-4.57588	1.09864
H	-1.83117	-1.58653	2.78882
C	-1.96664	0.95192	3.53632
C	-1.77829	2.6727	1.17484
O	-4.72599	-2.07817	-1.8555
H	-2.01451	0.13727	4.26247
H	-2.90685	1.51155	3.60161
H	-1.1651	1.63417	3.8404
C	1.33885	0.90014	-1.18051
C	2.78222	0.7045	-0.97417
C	0.98286	1.56311	-2.49546
H	0.82551	0.14028	0.69802
H	1.71072	2.34693	-2.73348
H	1.02354	0.83885	-3.31824

H	-0.00706	2.01903	-2.48296
C	3.45828	-0.34983	-0.43945
H	3.3953	1.52857	-1.34244
C	4.9041	-0.21327	-0.33116
C	2.8202	-1.64036	0.01064
C	5.75246	-1.11539	0.21227
H	3.40133	-2.49853	-0.3424
H	2.77586	-1.71192	1.10402
H	1.80366	-1.73393	-0.37307
H	5.32606	0.71215	-0.7165
C	7.20362	-0.92512	0.30174
H	5.40672	-2.05374	0.63063
O	7.97122	-1.7387	0.79632
O	7.64608	0.2489	-0.21675
H	8.61061	0.25659	-0.09798
H	-2.07637	3.02195	2.15821
C	-1.69143	3.74953	0.16796
O	-1.43783	3.5852	-1.02735
C	-1.96048	5.14584	0.7025
H	-1.25011	5.3925	1.49979
H	-1.8757	5.8757	-0.10336
H	-2.96275	5.19928	1.14267

Table S22. Coordinates (Ångstroms) for conformer **1b-8**

Atoms	X	Y	Z
C	-2.80712	-2.76639	0.71038
C	-1.80927	-1.59463	0.69787
C	-4.27862	-2.31104	0.5825
C	-1.84033	-0.79515	1.97484
C	-1.98869	-0.68069	-0.53194
C	-1.04678	0.55128	-0.40444
H	-1.6788	-1.23849	-1.42501
C	-3.47484	-0.24797	-0.75005
H	-4.48904	-1.63956	1.4288
C	-4.39843	-1.46473	-0.67857
C	-5.26075	-3.48095	0.60649
C	-3.69295	0.5449	-2.04217
H	-3.76122	0.38027	0.10642
H	-1.20054	1.21249	-1.25126
C	-1.35683	1.33564	0.86718
C	0.41535	0.14618	-0.377
H	-0.80724	-2.0458	0.62729
H	-2.68677	-3.34956	1.63099
H	-2.57249	-3.44243	-0.12176
C	-1.64668	0.53943	2.0755
H	-4.75671	0.74523	-2.19036
H	-3.16806	1.5024	-2.01672
H	-3.34048	-0.01834	-2.91252
H	-6.29298	-3.13262	0.51519
H	-5.06896	-4.17012	-0.22173
H	-5.1665	-4.03767	1.54375
H	-2.02614	-1.35869	2.88871
C	-1.73373	1.22054	3.42201
C	-1.32772	2.69574	0.92673
O	-5.17581	-1.74404	-1.57893
H	-1.89209	0.4854	4.21428
H	-2.56387	1.9353	3.46006
H	-0.81907	1.77836	3.65154
C	1.27019	0.13096	-1.42315
C	2.66582	-0.30385	-1.26676
C	0.88298	0.46872	-2.84753
H	0.79091	-0.18807	0.58672
H	1.44777	1.33572	-3.20927
H	1.12914	-0.36712	-3.51345

H	-0.17907	0.68627	-2.95954
C	3.56006	-0.02717	-0.27728
H	3.03978	-0.90564	-2.09659
C	4.87486	-0.64266	-0.39297
C	3.29192	0.88449	0.89455
C	5.88644	-0.5374	0.49838
H	3.161	0.3186	1.82482
H	4.13672	1.56349	1.05067
H	2.39356	1.48224	0.73835
H	5.04068	-1.2508	-1.27932
C	7.1928	-1.18295	0.33491
H	5.79301	0.03802	1.41229
O	8.10655	-1.09274	1.14279
O	7.31927	-1.90666	-0.80663
H	8.21658	-2.27977	-0.79829
H	-1.52287	3.1678	1.88424
C	-1.07763	3.65507	-0.16827
O	-0.85415	3.34684	-1.34054
C	-1.12041	5.11756	0.2413
H	-0.37707	5.31745	1.02143
H	-0.92395	5.75231	-0.62348
H	-2.09999	5.36669	0.66514

Table S23. Coordinates (Ångstroms) for conformer **1b-17**

Atoms	X	Y	Z
C	-2.74291	-2.83745	0.71289
C	-1.86036	-1.58139	0.82507
C	-4.21016	-2.51803	0.34707
C	-2.15684	-0.77548	2.06346
C	-1.92431	-0.70094	-0.44025
C	-1.13075	0.61831	-0.19656
H	-1.4268	-1.23526	-1.25964
C	-3.38916	-0.40845	-0.90049
H	-4.60799	-1.85761	1.13258
C	-4.20421	-1.70221	-0.93947
C	-5.0781	-3.77151	0.24994
C	-3.46774	0.34221	-2.23331
H	-3.8616	0.20372	-0.11792
H	-1.21447	1.24712	-1.07663
C	-1.71305	1.3807	0.98994
C	0.33322	0.34319	0.06696
H	-0.82508	-1.94411	0.92062
H	-2.71775	-3.39602	1.65593
H	-2.32293	-3.49973	-0.05487
C	-2.10396	0.57228	2.16092
H	-4.50787	0.44239	-2.55206
H	-3.03945	1.34376	-2.15374
H	-2.93332	-0.19941	-3.02066
H	-6.11037	-3.51891	-0.00626
H	-4.70128	-4.44944	-0.52212
H	-5.08104	-4.30703	1.20411
H	-2.42562	-1.34577	2.95211
C	-2.45584	1.25539	3.46244
C	-1.83692	2.7363	1.01248
O	-4.80572	-2.0605	-1.94074
H	-2.65816	0.51651	4.24108
H	-3.34793	1.88405	3.36013
H	-1.64389	1.90409	3.8093
C	1.36696	0.43648	-0.80945
C	2.67899	0.06099	-0.28062
C	1.18703	0.92088	-2.23113
H	0.5678	0.01207	1.07748
H	0.25193	1.4691	-2.34534
H	1.99729	1.59579	-2.51845

H	1.17997	0.09088	-2.94678
C	3.89766	-0.15196	-0.86134
H	2.66518	-0.07895	0.79967
C	4.98498	-0.46348	0.05288
C	4.22985	-0.1122	-2.33422
C	6.28127	-0.6773	-0.27067
H	4.78249	-1.01528	-2.61552
H	3.35221	-0.05419	-2.97086
H	4.87885	0.74038	-2.56898
H	4.73227	-0.52061	1.11031
C	7.26709	-0.97372	0.77424
H	6.64535	-0.63678	-1.29063
O	7.05209	-1.06148	1.97537
O	8.50971	-1.15202	0.25583
H	9.09992	-1.34251	1.00473
H	-2.22396	3.19658	1.91584
C	-1.52968	3.70369	-0.06057
O	-1.1126	3.40862	-1.18265
C	-1.77708	5.15815	0.30113
H	-2.82696	5.30943	0.57702
H	-1.17598	5.44192	1.1725
H	-1.52464	5.80023	-0.54346

Table S24. Coordinates (Ångstroms) for conformer **1b-25**

Atoms	X	Y	Z
C	-2.73459	-2.83739	0.70224
C	-1.84916	-1.58352	0.81634
C	-4.20305	-2.51349	0.34533
C	-2.13783	-0.78339	2.06032
C	-1.91788	-0.69649	-0.44412
C	-1.1206	0.62001	-0.19773
H	-1.42556	-1.22752	-1.26877
C	-3.38452	-0.39894	-0.89532
H	-4.59579	-1.85638	1.13617
C	-4.20199	-1.69107	-0.93702
C	-5.07375	-3.76489	0.24613
C	-3.46858	0.35899	-2.22368
H	-3.85185	0.20986	-0.10707
H	-1.20759	1.25341	-1.07418
C	-1.69564	1.37742	0.99548
C	0.3442	0.34091	0.05715
H	-0.81408	-1.94862	0.90477
H	-2.70576	-3.40077	1.64231
H	-2.31963	-3.49653	-0.07094
C	-2.08191	0.56375	2.16439
H	-4.51015	0.46258	-2.53658
H	-3.0382	1.35939	-2.14092
H	-2.93908	-0.17929	-3.01661
H	-6.10682	-3.50911	-0.00364
H	-4.70203	-4.43959	-0.5312
H	-5.07297	-4.30523	1.19759
H	-2.40325	-1.3577	2.94739
C	-2.42593	1.24083	3.47116
C	-1.81725	2.73307	1.02543
O	-4.80892	-2.0432	-1.9372
H	-2.62549	0.49835	4.24708
H	-3.31746	1.87149	3.37664
H	-1.61111	1.88643	3.81711
C	1.37383	0.43709	-0.82372
C	2.688	0.05672	-0.30326
C	1.18767	0.9296	-2.24181
H	0.58305	0.00401	1.06475
H	1.17546	0.10362	-2.96204
H	0.25299	1.48009	-2.34828

H	1.99766	1.60469	-2.52947
C	3.9033	-0.15625	-0.89065
H	2.67887	-0.0875	0.77657
C	4.99549	-0.47347	0.01827
C	4.22691	-0.11181	-2.36512
C	6.28833	-0.68566	-0.32352
H	4.88031	0.7373	-2.60021
H	4.77112	-1.01746	-2.65458
H	3.34562	-0.04459	-2.99563
H	4.73833	-0.5325	1.07331
C	7.34873	-0.99228	0.63915
H	6.63021	-0.63743	-1.35091
O	8.51859	-1.18104	0.33423
O	6.93202	-1.05493	1.93074
H	7.71834	-1.26217	2.46277
H	-2.19872	3.18946	1.93313
C	-1.51428	3.70538	-0.04439
O	-1.10469	3.4153	-1.17053
C	-1.75633	5.15844	0.32641
H	-1.15002	5.43618	1.1961
H	-1.50698	5.80449	-0.51606
H	-2.80441	5.31055	0.60868

Table S25. Coordinates (Ångstroms) for conformer **1b-39**

Atoms	X	Y	Z
C	-2.29663	-2.91933	0.46659
C	-1.50222	-1.60654	0.59135
C	-3.81741	-2.69596	0.30755
C	-1.69582	-0.93817	1.92752
C	-1.78791	-0.62982	-0.56871
C	-1.07594	0.7287	-0.29669
H	-1.35654	-1.04765	-1.48739
C	-3.31739	-0.4291	-0.82569
H	-4.16162	-2.13735	1.19116
C	-4.02945	-1.78074	-0.89133
C	-4.59611	-4.00572	0.1993
C	-3.61848	0.42223	-2.06287
H	-3.7343	0.07081	0.06129
H	-1.31983	1.41907	-1.09707
C	-1.56483	1.33172	1.0175
C	0.4317	0.57305	-0.23846
H	-0.44016	-1.88978	0.53017
H	-2.11238	-3.55139	1.34329
H	-1.92819	-3.47643	-0.40429
C	-1.73177	0.39568	2.14628
H	-4.69493	0.45757	-2.2464
H	-3.26134	1.44689	-1.93925
H	-3.14665	-0.00222	-2.95512
H	-5.66742	-3.82044	0.08475
H	-4.26574	-4.58716	-0.667
H	-4.44541	-4.61326	1.09678
H	-1.80302	-1.60341	2.78374
C	-1.9606	0.93077	3.54127
C	-1.80131	2.66203	1.18533
O	-4.71658	-2.10766	-1.84731
H	-1.99665	0.11302	4.26461
H	-2.90608	1.48063	3.6132
H	-1.16451	1.61998	3.84395
C	1.32178	0.93089	-1.19127
C	2.7678	0.74898	-0.99204
C	0.95309	1.59643	-2.50141
H	0.82473	0.15698	0.68594
H	0.9975	0.87656	-3.32785
H	-0.04138	2.04209	-2.48234

H	1.67178	2.38882	-2.73888
C	3.45627	-0.29983	-0.46189
H	3.37162	1.57922	-1.36153
C	4.89971	-0.15023	-0.3592
C	2.83319	-1.59736	-0.0104
C	5.76672	-1.03844	0.17538
H	3.42532	-2.44874	-0.36151
H	2.78889	-1.66761	1.08306
H	1.81807	-1.70405	-0.39467
H	5.32101	0.77806	-0.74174
C	7.20458	-0.74284	0.21477
H	5.44908	-1.9853	0.59654
O	7.75068	0.26761	-0.20446
O	7.90725	-1.7506	0.79166
H	8.84192	-1.48248	0.78085
H	-2.0974	3.00476	2.1716
C	-1.73034	3.74338	0.18203
O	-1.48352	3.58563	-1.01558
C	-2.00754	5.13531	0.72369
H	-1.29417	5.38481	1.51742
H	-1.93397	5.86895	-0.07983
H	-3.00739	5.17872	1.17044
